# Deconvolution of expression microarray data reveals ^131^I-induced responses otherwise undetected in thyroid tissue

**DOI:** 10.1371/journal.pone.0197911

**Published:** 2018-07-12

**Authors:** Britta Langen, Nils Rudqvist, Johan Spetz, Khalil Helou, Eva Forssell-Aronsson

**Affiliations:** 1 Department of Radiation Physics, Institute of Clinical Sciences, Sahlgrenska Cancer Center, Sahlgrenska Academy at the University of Gothenburg, Sahlgrenska University Hospital, Gothenburg, Sweden; 2 Department of Applied Physics, Chalmers University of Technology, Gothenburg, Sweden; 3 Department of Oncology, Institute of Clinical Sciences, Sahlgrenska Cancer Center, Sahlgrenska Academy at the University of Gothenburg, Sahlgrenska University Hospital, Gothenburg, Sweden; 4 Department of Medical Physics and Biomedical Engineering, Sahlgrenska University Hospital, Gothenburg, Sweden; University of Louisville, UNITED STATES

## Abstract

High-throughput gene expression analysis is increasingly used in radiation research for discovery of damage-related or absorbed dose-dependent biomarkers. In tissue samples, cell type-specific responses can be masked in expression data due to mixed cell populations which can preclude biomarker discovery. In this study, we deconvolved microarray data from thyroid tissue in order to assess possible bias from mixed cell type data. Transcript expression data [GSE66303] from mouse thyroid that received 5.9 Gy from ^131^I over 24 h (or 0 Gy from mock treatment) were deconvolved by cell frequency of follicular cells and C-cells using csSAM and R and processed with Nexus Expression. Literature-based signature genes were used to assess the relative impact from ionizing radiation (IR) or thyroid hormones (TH). Regulation of cellular functions was inferred by enriched biological processes according to Gene Ontology terms. We found that deconvolution increased the detection rate of significantly regulated transcripts including the biomarker candidate family of kallikrein transcripts. Detection of IR-associated and TH-responding signature genes was also increased in deconvolved data, while the dominating trend of TH-responding genes was reproduced. Importantly, responses in biological processes for DNA integrity, gene expression integrity, and cellular stress were not detected in convoluted data–which was in disagreement with expected dose-response relationships–but upon deconvolution in follicular cells and C-cells. In conclusion, previously reported trends of ^131^I-induced transcriptional responses in thyroid were reproduced with deconvolved data and usually with a higher detection rate. Deconvolution also resolved an issue with detecting damage and stress responses in enriched data, and may reduce false negatives in other contexts as well. These findings indicate that deconvolution can optimize microarray data analysis of heterogeneous sample material for biomarker screening or other clinical applications.

## Background

Gene expression profiles are specific for every cell type and determine not only cellular function but also cellular responses to diverse or specific stressors. In *in vivo* research, studies are often performed with heterogeneous tissue samples, since cell type-specific separation of sample material usually deteriorates sample integrity impeding subsequent analysis. mRNA is used in high-throughput expression microarrays for analysis of genome-wide transcriptional regulation. The single-stranded nucleic acids, however, are exposed to natural degradation in tissue samples. Therefore, extraction and purification of mRNA must be performed expeditiously to avoid further degradation. Analysis of single cell types *in vitro* would prevent convolution of data, yet also abrogate the *in vivo* context. At present, this dilemma cannot readily be solved experimentally. However, computational deconvolution methods can be used to extract cell type-specific information from gene expression data obtained from heterogeneous tissue samples [1]. For biomarker discovery, accuracy of observed transcriptional regulation in response to a given stressor is essential. In statistics, false positives (type I error) are considered more severe for experimental research, since allegedly positive instances are reported and committed to the knowledge base creating misleading information [2]. In biomarker discovery, false negatives (type II error) can be regarded as similarly severe, since potential biomarkers would remain undiscovered, which may preclude subsequent (successful) trial studies.

The thyroid gland is a risk organ in radionuclide therapy using ^131^I and ^211^At, since their halogenic properties result in high uptake in thyroid tissue [3–9]. In our group, we have performed several expression microarray studies using mouse and rat as model systems for discovery of biomarkers for ionizing radiation (IR) exposure. We have studied differential transcript expression in thyroid tissue in response to i.v. administered ^211^At and ^131^I [10–13]. Furthermore, we have studied the impact of systemic effects *in vivo* from the irradiated thyroid on transcriptional regulation in the kidneys, liver, lungs, and spleen [14–19]. We also performed expression microarray studies for biomarker discovery in cortical and medullary kidney tissues after i.v. administration of ^177^Lu and ^177^Lu-octreotate [20,21]. The biomarker candidate genes proposed in these studies, however, were obtained from tissue expression data and other significant cell type-specific gene regulation may have been missed due to convoluted expression signals.

Regarding cell composition of the thyroid in particular, the gland is composed of follicular cells, also called thyroid epithelial cells, which line the follicular lumen and secrete the thyroid hormones (TH) triiodothyronine and thyroxine. Parafollicular cells, so called C-cells, are occasionally interspersed between follicles and secrete another hormone named calcitonin. Transcript regulation indicative of absorbed dose level or induced cellular damage in either cell type may not be detected in convoluted data. In recent studies, kallikrein transcripts were proposed as biomarkers of ^131^I and ^211^At in thyroid tissue showing different regulation patterns in relation to absorbed dose and radiation quality [22] as well as diurnal robustness regarding significant detection [16]. Accordingly, the effect of convolution on detection rate of these biomarker candidates should be investigated. Signature genes that are associated with IR- or TH-induced regulation have been used previously to evaluate the extent of regulation induced by ionizing radiation as the initial stressor or as a response to differentially regulated thyroid hormones: the analysis indicated IR-induced changes of TH regulation in thyroid tissue, as well as changes in TH-responding genes in non-thyroid tissues, i.e. that non-thyroid tissues were subject to systemic effects in parallel to immediate radiation exposure [18]. For further details on signature gene analysis, please refer to previous work [18].

Recently, biomarker screening *in vivo* has gained importance in radiation research and a host of whole-tissue omics expression data have been committed to the knowledge base. A computational method is desired to increase the potential biomarker gain from these experimental efforts and reduce the rate of false negatives. An estimation of cell-specific expression profiles can be obtained using statistical permutation analysis, but to the best of our knowledge, this has not been demonstrated before. With this concept study, we assess the usefulness of deconvolution for increasing the detection rate of biomarker candidates in whole tissue samples.

Specifically, the aim of this study was to investigate if the detection rate of significantly regulated transcripts in thyroid microarray data could be increased by deconvolution, and consequently, to what extent results on radiation-induced responses would differ–or remain similar–between convoluted and cell type-specific data sets. Gene expression microarray data of thyroid tissue was taken from a previous study on diurnal variation of gene regulation in mice in response to ^131^I [16]. It should be noted that there are different methods to deconvolve gene expression data. For instance, marker gene probesets can be used as reference expression signals for each cell type, as has been demonstrated for deconvolution of gene expression data from heterogeneous brain tissue [23]. In comparison, thyroid tissue has a simple microanatomy consisting of only two tissue-specific cell types whose frequency can be easily estimated by histological analysis. Hence, a deconvolution method based on cell frequency was chosen over marker probeset-based deconvolution. In particular, the csSAM (cell type-specific significance analysis of microarrays) package in R was used, which deconvolves cell type-specific expression based on cell type frequency in the sample material using least-squares fit [24].

A comparison of detection rate of analytical endpoints between data sets was performed to evaluate potential bias from convoluted microarray data. Endpoints of interest were total transcript regulation, kallikrein transcript regulation, responses in IR-associated and TH-responding signature genes, and regulation of transcript-associated cellular functions. Specifically, the analytical robustness of previously reported results [16] on transcriptional regulation in mouse thyroid following i.v. ^131^I administration was evaluated.

## Materials and methods

### 2.1 Experimental design

In the previous experimental study, female BALB/c nude mice (Charles River Laboratories International, Inc.; Salzfeld, Germany) were i.v. injected with 90 kBq ^131^I (prepared in physiological saline) at different times during the day (n = 4/group) and killed after 24 h [16]. Paired control groups were mock-treated with physiological saline (n = 3–4/group). Thyroids were excised, flash-frozen in liquid nitrogen, and stored at -80°C until extraction of total RNA (RIN value of at least 6.0). Illumina MouseRef-8 Whole-Genome Expression BeadChips (Illumina; San Diego, CA, USA) were used for microarray analysis as described elsewhere [16]. Absorbed dose to the thyroid over 24 h was estimated as 5.9 Gy using the MIRD formalism; please refer to Langen *et al*. (2015) for details on dosimetry [16]. The gene expression data have been deposited in NCBI's Gene Expression Omnibus with GEO accession GSE66303. In the present study, thyroid data of groups treated at 9.00 am with ^131^I or physiological saline (n = 4/group) were used for deconvolution analysis.

### 2.2 Quantitative real-time PCR (QPCR)

The quantitative real-time polymerase chain reaction (QPCR) assay was performed to validate microarray results. For each specimen (n = 4/group), cDNA was synthesized from 1 μg total RNA, i.e. from the same RNA eluate committed to microarray analysis, using SuperScript™ III First-Strand Synthesis SuperMix by Invitrogen (Thermo Fisher Scientific; Carlsbad, CA, USA) according to the manufacturer´s protocol. Validated TaqMan® Gene Expression Assays and the TaqMan® Gene Expression Master Mix were obtained from Applied Biosystems (Thermo Fisher Scientific; Carlsbad, CA, USA). Ten target genes were selected for QPCR validation that showed significant regulation in thyroid microarray data (*Atp2a1*, *Ccnd1*, *Ccng1*, *Klk1b16*, *Myh2*, *Pck1*, *Pvalb*, *Tpm2*). Three constitutive genes that showed homogeneous expression across the entire data set (*Cyp11b1*, *Rptn*, and *Prg3*) were used for normalization using the arithmetic mean of Ct values. cDNA samples were run in triplicate for each assay and the ΔΔCt method was used for quantification of differential expression.

### 2.3 Cell frequency analysis of thyroid tissue

Thyroid glands were excised from two female BALB/c nude mice aged to six weeks and five months, respectively. Thyroids were fixed in formalin (4%, phosphate buffered), dehydrated, and embedded in paraffin following standard procedure. Transverse microtome sections (4 μm) were stained with hematoxylin-eosin following standard protocol. Images were acquired with a Nikon DS-Fi1 camera system (Nikon; Japan) using an Olympus BX45 microscope (Olympus; Japan) and NIS-Elements F 2.30 software (Nikon; Japan). Eight microscope images (20x magnification) from each mouse were analyzed, and in total, over 3,100 thyroid cells were counted to calculate cell frequency of follicular cells and C-cells.

### 2.4 Deconvolution of microarray data

Deconvolution was performed with csSAM package version 1.2.4 [24] retrieved from the CRAN repository (https://cran.r-project.org/) using the R statistical computing environment version 3.0.1 (http://www.r-project.org). The function csfit {csSAM} deconvolves cell type-specific expression based on the frequency of each cell type in the sample material using least-squares fit. Input data was log-transformed since it generally results in lower false discovery rates as opposed to log transformation after deconvolution [24,25]. Cell frequency of C-cells relative to follicular cells was set to 0.11, 0.1046, and 0.1154 (three values needed: mean value and mean value ±SEM were chosen) according to cell counting results reported below. Deconvolved datasets are available via the figshare repository (https://doi.org/10.6084/m9.figshare.6715349). In order to validate overall results concerning this input parameter, we also performed deconvolution and supplemental data analysis (as indicated) with a lower C-cell frequency estimate of 0.05 (+/- 0.005) as indicated below.

### 2.5 Data analysis

Data processing of deconvolved expression data was performed with Nexus Expression 3.0 (BioDiscovery; El Segundo, CA, USA). The false discovery rate (FDR) was controlled using the Benjamini-Hochberg method with an adjusted p-value cutoff of 0.01 and significantly regulated transcripts, hereafter referred to as (differentially) regulated, were identified with a log_2_ ratio threshold of at least 0.58 (fold change ≥ 1.5) [2]. The pool size for intensity based pooling was set to 200. The same statistical processing was used for the convoluted microarray data in the previous study [16]. The relative impact of IR- and TH-induced regulation on observed responses was assessed by significant regulation of respective signature genes as described previously [18]. Briefly, 56 genes associated with IR-induced responses were adapted from Snyder and Morgan, and Chaudhry [26,27]; 61 genes and gene groups (encoding multimeric proteins) associated with TH-induced regulation were compiled from literature [18]. Supplemental analysis was performed for data deconvolved with a C-cell frequency of 0.05, in order to validate overall results with regard to high vs. low C-cell frequency estimation: a cluster analysis was performed for overall transcript regulation using gplots {heatmap.2} in R (version 3.4.3); supplemental signature gene analysis was performed as described above.

Cellular function of transcript regulation was analyzed by categorization of enriched biological processes according to Gene Ontology (http://www.geneontology.org) terms as described elsewhere [15]. Briefly, significance of enrichment was accepted with a p-value of less than 0.05. The strength of response was expressed as the percentage of scored vs. filtered transcripts of all significant biological processes grouped in a given category or subcategory. The result was visualized as a heat map also stating the sum of scored and filtered transcripts (first and second value, respectively) for each category and subcategory.

## Results

### 3.1 Cell frequency in BALB/c nude mouse thyroid

**Fig 1:**
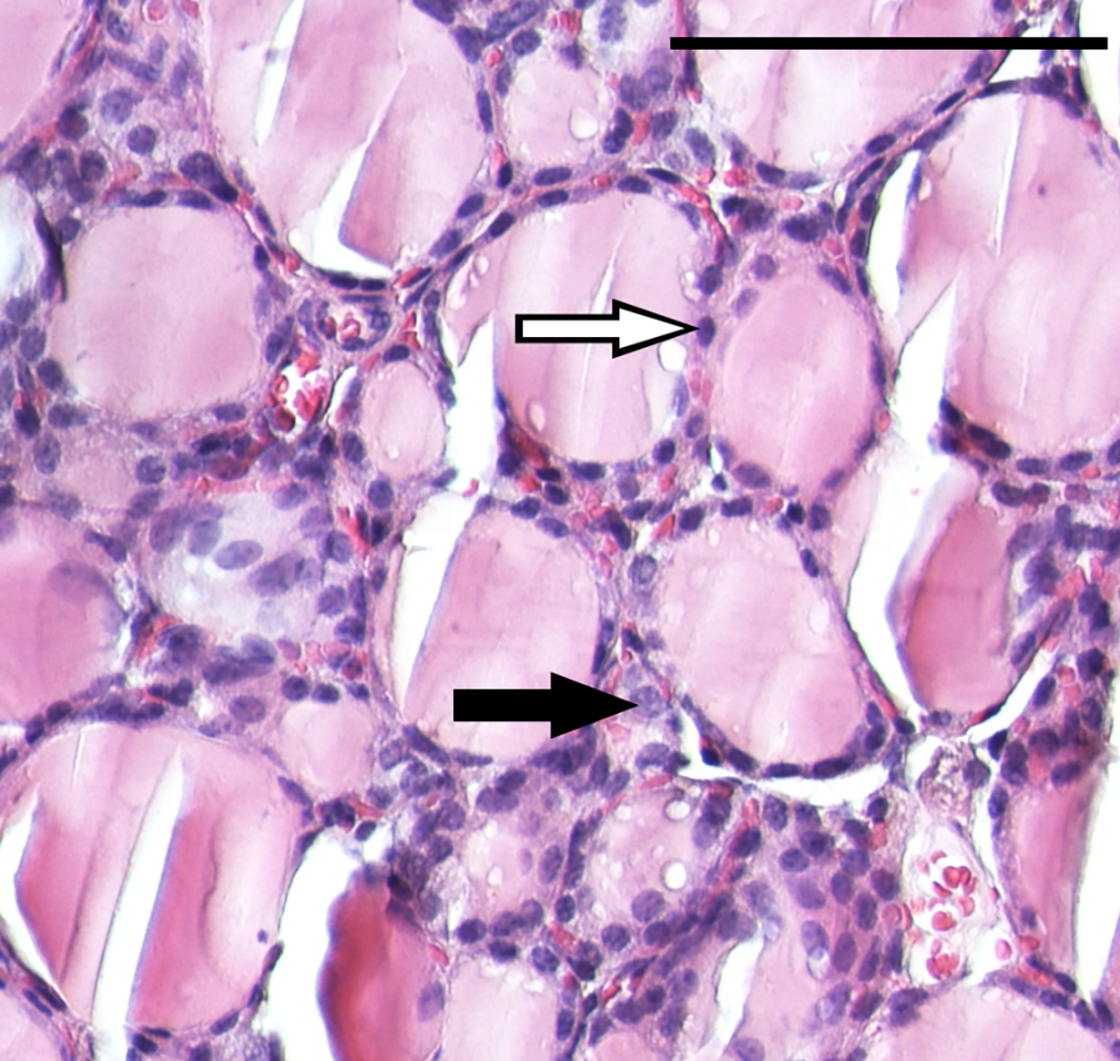
Microanatomy of mouse thyroid tissue. The image shows a 20x magnification of a transverse microtome section of normal thyroid tissue from a 5-month-old female BALB/c nude mouse. Thyroid epithelial cells (follicular cells) and parafollicular cells (C-cells) are indicated exemplarily by white and black arrow, respectively. Scale bar (top right), 100μm.

Heterogeneity of thyroid samples regarding cell type is shown in a (magnified section of a) representative image used in cell counting analysis (Fig 1). The cell frequency of C-cells relative to follicular cells in tissue samples was estimated to be approximately 11% with a SEM of 0.54%. Morphological differences between 6-week-old and 5-month-old mice were not observed, while irregular C-cell distribution was seen. Due to the limited number of specimens, we acknowledge lack of accuracy of the C-cell frequency estimate and potential overestimation.

### 3.2 Differences in total significant transcript regulation

**Fig 2:**
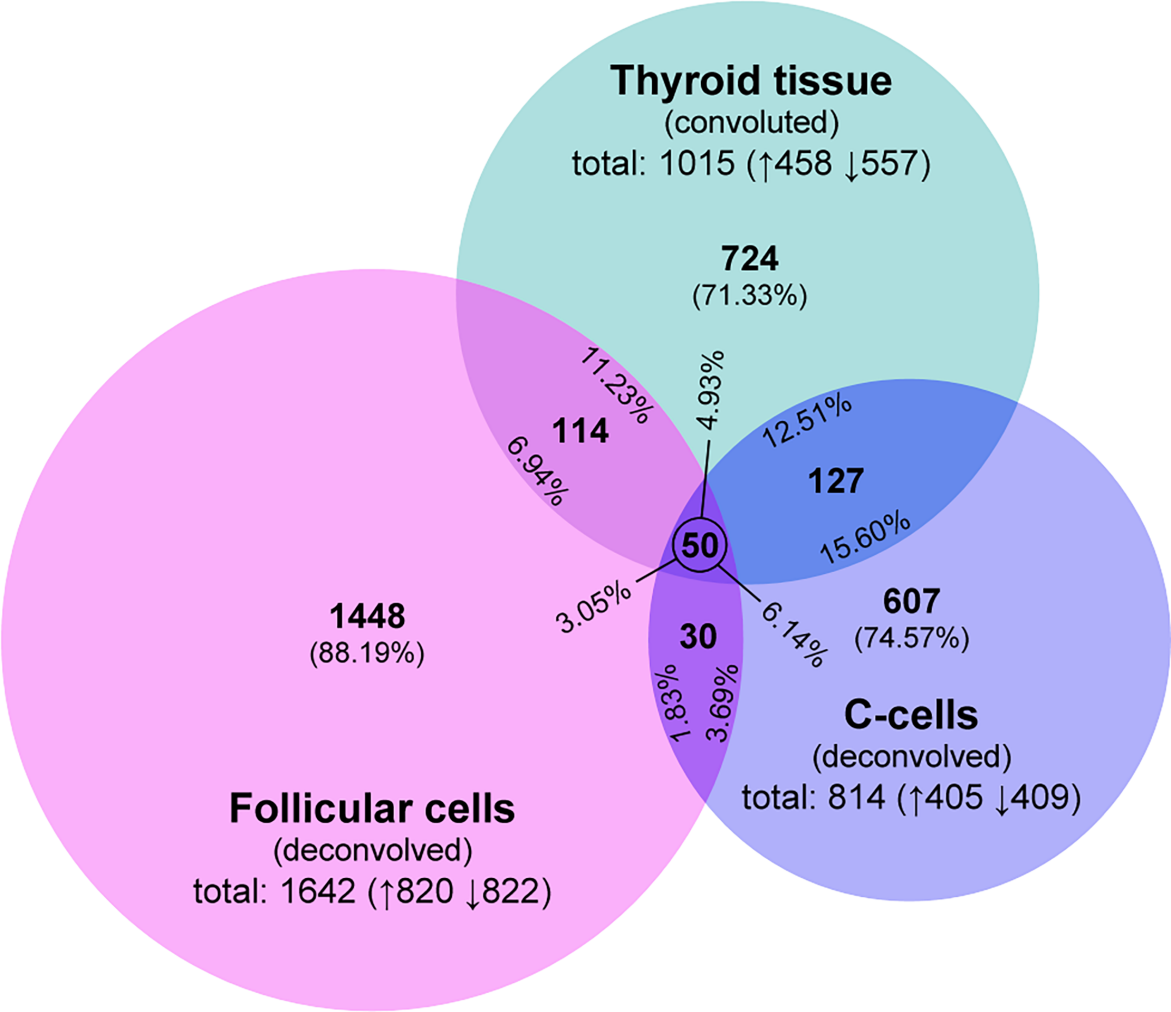
Significant transcript regulation. The Venn diagram shows the number of up- and down-regulated transcripts (up- and down-arrows, respectively) for each data set and the distribution of shared transcripts between data sets. Results of transcript regulation for thyroid tissue adapted from Langen *et al*. [16].

The total significant transcript regulation, i.e. the number of differentially regulated transcripts compared between the ^131^I-treated group and the mock-treated control group, showed clear differences between convoluted data for thyroid tissue and deconvolved data for follicular cells and C-cells (Fig 2). Analysis of convoluted thyroid data yielded 1015 significantly regulated transcripts with up- and down-regulation differing by nearly 100 transcripts. Thyroid microarray data was validated using QPCR (S1 Table). In deconvolved data, significant transcript regulation was distinctly higher in follicular cells (1642 transcripts) but lower in C-cells (814 transcripts), while up- and down-regulation were on the same level. The potential impact of high vs. low estimation of C-cell frequency can be considered low, since cluster analysis of data deconvolved with either 0.11 or 0.05 relative frequency showed highly similar heatmap profiles (please see S1 Fig). Complete lists of significantly regulated transcripts in the thyroid, follicular cells, and C-cells are shown in S2–S4 Tables, respectively.

### 3.3 Differences in significant regulation of kallikrein transcripts

**Fig 3:**
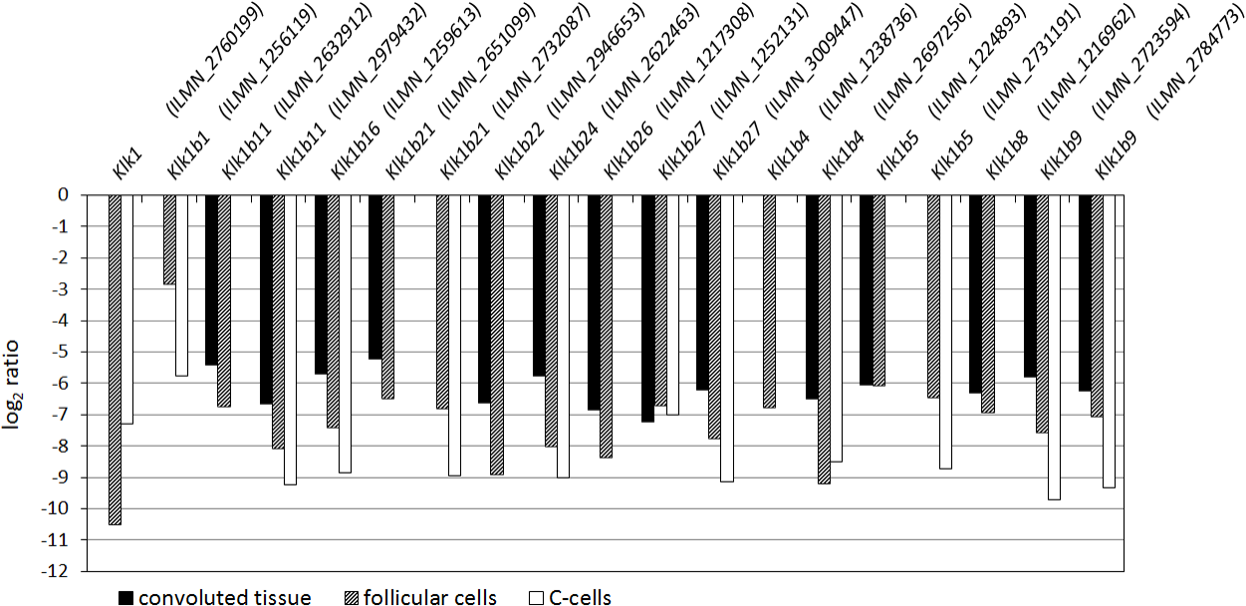
Kallikrein transcript regulation in convoluted and deconvolved data. Significant regulation of kallikrein transcripts is shown in convoluted and deconvolved expression microarray data from normal mouse thyroid. Regulation is in response to 90 kBq ^131^I after 24 h following i.v. administration. Illumina probe ID (ILMN) is written in parentheses. Black, convoluted thyroid tissue; hashed, deconvolved follicular cells; white, deconvolved C-cells.

Pronounced differences in detection rate of significantly regulated transcripts of the kallikrein family were observed between convoluted and deconvolved data sets (Fig 3; see S5 Table for respective fold change values and adjusted p-values). Notably, all but one gene belonged to the Mus musculus species-specific kallikrein subfamily *Klk1b*. Overall, 19 kallikrein transcripts were detected, all of which were detected in at least one deconvolved data set, while only 14 transcripts were detected in convoluted thyroid data. Specifically, 8 transcript probes were detected in thyroid tissue and in both follicular cells and C-cells, while 6 transcript probes were detected in thyroid tissue and in either follicular cells or C-cells. In contrast, 4 transcript probes were detected in both follicular cells and C-cells but not in convoluted thyroid data, and one transcript probe (*Klk1b4*, ILMN_1238736) was detected in only follicular cells but neither in C-cells nor thyroid tissue. Interestingly, gene regulation of *Klk1b5* was detected in all three data sets but with different transcript probes, i.e. *Klk1b5* (ILMN_1224893) in thyroid tissue and follicular cells and *Klk1b5* (ILMN_2731191) in follicular cells and C-cells.

Furthermore, most transcript probes that were detected in at least two data sets showed pronounced differences in log_2_ ratio, while only three transcript probes showed similar expression levels in convoluted and deconvolved data sets, i.e. *Klk1b27* (ILMN_1252131), *Klk1b5* (ILMN_1224893) and *Klk1b8* (ILMN_1216962). All significantly regulated kallikrein transcript probes were down-regulated with generally high log_2_ ratio values, i.e. deconvolution did not change direction of regulation among this set of genes.

### 3.4 Differences in signature gene responses

For both IR-associated and TH-responding signature genes, detection rate of regulated genes was higher in deconvolved data than in convoluted data (Tables 1 and 2). This trend was also reproduced with the supplemental deconvolution analysis using a C-cell frequency estimate of 5% (see S6 and S7 Tables, respectively). Few of these genes (probes) were detected in more than one data set. Regarding IR-associated signature genes, 4 genes (6 probes) were detected in thyroid tissue while 7 genes (7 probes) were identified in deconvolved data, i.e. 6 genes (6 probes) in follicular cells and 1 gene (1 probe) in C-cells (Table 1). Only two genes were detected in both convoluted and deconvolved data: *Gjb2* (ILMN_2999627) was down-regulated with -2.9 log_2_ ratio in thyroid tissue and down-regulated with -5.1 log_2_ ratio in C-cells. The other transcript probe for *Gjb2*, i.e. ILMN_1227148 which targets the same transcript variant as ILMN_2999627, was only detected in convoluted data, however. Concerning *Ccng1* (ILMN_2500276), deconvolution changed direction of regulation from 0.81 log_2_ ratio in thyroid tissue to -2.5 log_2_ ratio in follicular cells. Regarding TH-responding signature genes, 10 genes (13 probes) were detected in thyroid tissue (Table 2). In follicular cells, 11 genes (11 probes) were detected, while 6 genes (6 probes) were detected in C-cells, and only one probe (*Atp2a1*, ILMN_2666864) was detected in both deconvolved data sets. As such, 3 more regulation instances were identified upon deconvolution. *Atp2a1* and *Cd44* were the only genes identified in all three data sets. *Atp2a1* regulation was detected with the same probe (ILMN_2666864) and showed large variation in log_2_ ratio between data sets, i.e. up-regulation ranged from 5.3 log_2_ ratio in thyroid tissue to 10.6 log_2_ ratio in C-cells. *Cd44* (ILMN_3114585) was differentially expressed in both thyroid tissue and follicular cells, with -0.69 and -3.2 log_2_ ratio, respectively, while *Cd44* (ILMN_2754990) was more strongly regulated in C-cells with -5.6 log_2_ ratio.

**Table 1:** Significant regulation of IR-associated gene signature.

Gene symbol (synonym)	Probe ID	Thyroid tissue (conv.)	Follicular cells (deconv.)	C-cells (deconv.)
		log_2_ ratio; fold change (adjusted p-value)		
*Ccnd1*^†^	ILMN_1221503	-0.92; -1.9 (0.0019)		
* *	ILMN_2601471	-0.91; -1.9 (0.0002)		
*Ccng1*	ILMN_2500276	0.81; 1.8 (0.0076)	-2.5; -5.7 (0.0096)	
*Cdkn1a*^†^	ILMN_2846775		-3.4; -11 (0.0001)	
*Fos*^†^	ILMN_2750515		5.0; 32 (0.0001)	
*Gjb2*	ILMN_1227148	-2.2; -4.6 (0.0000)		
* *	ILMN_2999627	-2.9; -7.5 (0.0000)		-5.1; -34 (0.0000)
*Naa35 (Mak10)*	ILMN_2828599		-2.6; -6.1 (0.0080)	
*Plcg2*	ILMN_2601833	-1.1; -2.1 (0.0000)		
*Trp53inp1*	ILMN_2506012		5.0; 32 (0.0000)	
*Trp53inp2*	ILMN_2457585		-3.0; -8.0 (0.0024)	

^†^Note that *Ccnd1*, *Cdkn1a* and *Fos* are reported as both IR-associated and TH-responding in the literature. Results of transcript regulation of respective signature gene for thyroid tissue adapted from Langen *et al*. [16]. Conv., convoluted data; deconv., deconvolved data. Adjusted p-values given as 0.0000 designate values below 10^−5^, i.e. values below the Nexus Expression limit.

**Table 2:** Significant regulation of TH-responding gene signature.

Gene symbol	Probe ID	Thyroid tissue (conv.)	Follicular cells (deconv.)	C-cells (deconv.)
		log_2_ ratio; fold change (adjusted p-value)		
*Atp2a1*	ILMN_2666864	5.3; 41 (0.0000)	7.8; 223 (0.0000)	11; 1552 (0.0000)
*Camkk1*	ILMN_1242310	* *	-2.2; -4.6 (0.0045)	
*Camkk2*	ILMN_1256263	* *		-3.9; -15 (0.0001)
*Ccnd1*	ILMN_1221503	-0.92; -1.9 (0.0019)		
	ILMN_2601471	-0.91; -1.9 (0.0002)		
*Cd44*	ILMN_3114585	-0.69; -1.6 (0.0001)	-3.2; -9.2 (0.0000)	
* *	ILMN_2754990	* *		-5.6; -49 (0.0000)
*Cdkn1a*^†^	ILMN_2846775	* *	-3.4; -11 (0.0001)	
*Egf*	ILMN_2684104	-4.9; -31 (0.0000)		-5.3; -39 (0.0000)
*Egfr*	ILMN_3128725	* *		-4.9; -30 (0.0095)
*Fos*^†^	ILMN_2750515	* *	5.0; 32 (0.0001)	
*Hnrnph3 (Hnrph3)*	ILMN_2958912	* *	5.7; 52 (0.0000)	
*Lmo2*	ILMN_2767605	-1.2; -2.3 (0.0000)		
*Mbp*	ILMN_3081854	* *	-2.9; -7.5 (0.0037)	
*Pck1*	ILMN_1213632	1.2; 2.3 (0.0001)		
*Pfkp*	ILMN_2673233	* *		5.7; 52 (0.0000)
*Pik3c2a*	ILMN_1252098	* *	-2.5; -5.7 (0.0087)	
*Prkag2*	ILMN_3161626	0.80; 1.7 (0.0002)		
*Prkca*	ILMN_1217890	* *	-1.9; -3.7 (0.0092)	
*Rcan2*	ILMN_3033007	* *	-5.0;-32 (0.0000)	
*Slc16a6*	ILMN_1258950	-0.91; -1.9 (0.0005)		
*Slc2a1*	ILMN_1258159	-0.94; -1.9 (0.0007)		
*Sms*	ILMN_1232323	* *	-2.3; -4.9 (0.0003)	
*Vldlr*	ILMN_1218264	1.1; 2.2 (0.0000)		
	ILMN_2515601	1.1; 2.2 (0.0000)		
	ILMN_2796472	1.1; 2.2 (0.0000)		

^†^Note that *Ccnd1*, *Cdkn1a* and *Fos* are reported as both IR-associated and TH-responding in the literature. Results of transcript regulation of respective signature genes for thyroid tissue adapted from Langen *et al*. [16]. Conv., convoluted data; deconv., deconvolved data. Adjusted p-values given as 0.0000 designate values below 10^−5^, i.e. values below the Nexus Expression limit.

### 3.5 Similarities and differences in associated cellular functions

**Fig 4:**
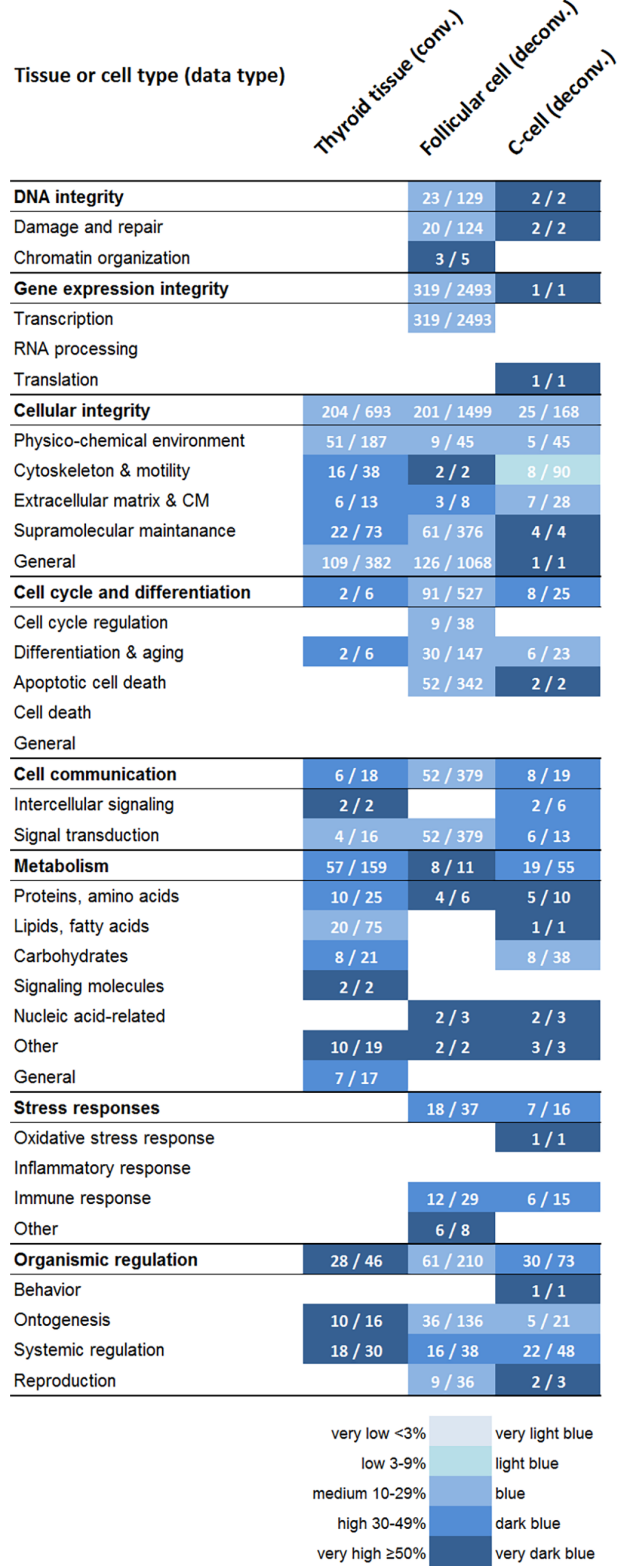
Regulation of biological processes enriched from convoluted and deconvolved data. Significant regulation of cellular function is shown for convoluted (conv.) and deconvolved (deconv.) transcript expression microarray data from normal mouse thyroid. Regulation is in response to 90 kBq ^131^I after 24 h following i.v. administration. Enriched biological processes were grouped into categories and subcategories of associated cellular function based on Gene Ontology terms. The percentage of scored vs. filtered transcripts is shown as very low <3%, low 3–9%, medium 10–29%, high 30–49%, and very high ≥50%, and colored as very light blue, light blue, blue, dark blue, and very dark blue, respectively. Values in white text show number of scored/filtered transcripts for each category and subcategory. For original results on enrichment analysis for convoluted thyroid tissue, please refer to Langen *et al*. [16].

The biological relevance of transcriptional changes was assessed by enriched biological processes that were categorized according to associated cellular function (Fig 4). Results of convoluted thyroid data have been reported and discussed comprehensively in a previous study [16]. Briefly, 5 of 8 categories showed significant responses in convoluted data, i.e. *cellular integrity*, *cell cycle and differentiation*, *cell communication*, *metabolism*, and *organismic regulation*, whereas no significant regulation was detected for *DNA integrity*, *gene expression integrity*, or *stress responses*. In deconvolved data, i.e. in both follicular cells and C-cells, regulation was detected in all categories, and in general, detection rate of significantly enriched biological processes was higher than in convoluted data. Regarding subcategories, 20 and 22 subcategories were detected in follicular cells and C-cells, respectively, while only 16 subcategories showed significant responses in thyroid tissue. Among the latter, only 2 subcategories, i.e. *signaling molecules (metabolism)* and *general (metabolism)*, showed responses in thyroid data but were negative in both follicular cells and C-cells.

The total number of scored transcripts (of enriched biological processes) differed strongly between data sets. Follicular cells showed the highest number with 773 scored transcripts, which was more than 2.5-fold higher than in convoluted thyroid data (297 scored transcripts). C-cells showed the lowest number with 100 scored transcripts, yet also a somewhat wider spread in regulated subcategories as described above. In general, regulation intensity of a category or subcategory, i.e. percentage of scored vs. filtered transcripts as well as number of filtered transcripts, also differed between the data sets.

## Discussion

Biomarker discovery using high-throughput techniques is a novel approach in radiation research. Despite the advantages of genome-wide screening for radiation effects, there are methodological drawbacks when analyzing whole-tissue data from *in vivo* experiments. Deconvolution of mixed cell type data is a step forward to overcome some of the limitations, such as missing biomarker candidates due to false negatives and under-representation of radiation-sensitive cell types. This work is the first to demonstrate the usefulness of the gene expression deconvolution technique in context with radiation-induced effects. As such, the total number of significantly regulated transcripts (probes) was strongly increased upon deconvolution, i.e. more than 600 transcript probes remained undetected in thyroid tissue as compared with follicular cells. This general finding demonstrated the usefulness of deconvolution for increasing detection rate of significantly regulated transcripts in mixed cell data. Nevertheless, it should be pointed out that detection rate is not a criterion per se for the quality of analysis. In context with biomarker discovery research, however, we reason that also false negatives can be considered a "severe" error, since potential biomarker genes would be missed and not validated in consecutive studies. In contrast, false positives would be erroneous candidates that would be eliminated from the candidate pool in validation studies. We assume that a large proportion of transcripts (probes) that were not detected in thyroid tissue data represent type II errors due to misrepresentation of cell type frequencies, i.e. lack thereof, in statistical analysis of convoluted data. Experimental validation studies are needed to confirm or reject this assumption. Another critical point is the change in fold-change of detected transcripts: if the same transcript (probe) was regulated in two or all data sets, the log_2_ ratios differed distinctly between data sets in most cases, which can have consequences for down-stream analysis and interpretation of biological effects. Thus, caution is advised when drawing conclusions from mixed microarray data and analytical end-points should be validated with appropriate tools to avoid bias.

Regarding the given experimental context, the specificity of deconvolved data may be of concern: in theory, frequency can describe microanatomic or microdosimetric properties, i.e. differentiating between different cell types, or between the hit and non-hit fraction. However, the latter can be disregarded, since the range of emitted electrons by ^131^I is long compared with follicle size [28,29], meaning that irradiation occurs homogeneously and both cell types were subject to the same absorbed dose. Accordingly, deconvolved data is specific for respective cell type frequency in this experimental design without a confounding factor of differential dose exposure.

Furthermore, it should be noted the 11% data subset is an estimate to differentiate the C-cell response from the follicular cell response in a purely statistical fashion: for one, the larger binning is possibly also enriched for low-abundance non-thyroid-specific cell types. In addition, by partitioning the signal based on cell type frequency, the more-abundant population (89%) can be expected to exhibit higher changes due to higher relative expression values. On the other hand, the semi-quantitative nature of the microarray analysis and downstream processing such as intensity-based pooling may scale down this effect. As such, the deconvolved data for follicular cells and C-cells represent an in silico statistical permutation analysis to improve biomarker discovery, and differences between both cohorts may indicate–but do not directly represent–biological differences. The biological specificity, i.e. relative response intensity of individual genes originating from a certain cell type, can only be validated by single-cell gene expression analysis, which would require another experimental design. Despite its advantages, in situ single-cell analysis would require a large number of measurements to achieve sufficient statistics, while single-cell analysis by means of tissue dissociation may introduce stress and study-unrelated responses, or eliminate signals owing to the microenvironment (depending on how long cells are kept in suspension).

Another aspect is to what extent the C-cell frequency estimate would impact obtained results. Age difference and biological variability between individuals were not observed in the morphological analysis, although they have been reported for rats [30,31]. The estimation of C-cell frequency has been reported with a relatively broad range, i.e. from a general 2–4% [32], over 11% in (older) rats [30], and up to 23% in mice [33]. The latter study, however, selected a specific thyroid area for their analysis, i.e. the middle third of the lobe, which has a higher concentration of C-cells, meaning that the average frequency in the whole organ would be distinctly lower. To the best of our knowledge, C-cell frequency and biological variability have not been documented for BALB/c nude mice. In this regard, we acknowledge that the cell frequency of 11% used for deconvolution may constitute an upper-end estimate. In order to assess the impact of a potentially lower C-cell frequency [32], a supplemental deconvolution analysis was performed with a 5% estimate. Cluster analysis showed that the transcriptional profiles for both follicular cell data and C-cell data was highly similar between 11% and 5% binning, and the overall trends of signature gene analysis were reproduced, which validated the deconvolution analysis in this range.

This work demonstrated that deconvolution analysis may allow for detection of differential radiation sensitivity between cell types, e.g. when a cell type is more radiosensitive but the distinct signature would be masked in convoluted data due to low cell frequency. This is an important aspect for biomarker screening, in particular if the respective cell type is critical for tissue function. Neglecting cell type-specific radiation sensitivity and respective expression signatures may yield inaccurate results for radiation risk assessment. A mean absorbed dose of 5.9 Gy over 24 h to thyroid was calculated for i.v. administration of 90 kBq ^131^I [16]. For this exposure condition, one ionizing radiation-associated transcript was detected in C-cells compared with six transcripts in follicular cells. Regarding C-cells, studies on cell type-specific IR-induced effects in normal rat thyroid tissue after ^131^I exposure, however, indicate increased radiation sensitivity compared with follicular cells [34,35]. Taking the number of regulated IR-associated genes as a measure, C-cells did not indicate increased radiation sensitivity in this study. However, the analysis is based on a single time-point measurement, and it should be noted that an increased response of IR-associated genes in C-cells may occur at earlier or later time points. Therefore, it is not possible to deduce from these data if the different expression signatures of follicular cells and C-cells are a result of differential radiation sensitivity.

TH-responding signature genes were used to estimate the extent of systemic effects that potentially influenced transcriptional responses in non-thyroid tissues after i.v. ^131^I administration. These genes are also expressed in thyroid tissue and allow for correlation between systemic effects in target tissues and the regulatory thyroid gland in our previous studies [15,16,18]. The higher regulation incidence of TH-responding signature genes compared with IR-associated signature genes, as previously discussed for convoluted thyroid data [16], was validated in deconvolved data with a distinctly increased detection rate considering both follicular cells and C-cells.

All of the kallikrein transcripts detected in convoluted thyroid data were detected in at least one, often both, deconvolved data sets. Moreover, several kallikrein transcripts were detected in deconvolved data that remained undetected in convoluted data. Deconvolution thus supported kallikrein transcripts as biomarkers for absorbed dose and/or induced thyroid damage and increased detection rate among transcripts of a specific gene family. As a result, we continue with further investigation of kallikrein genes and gene products as a biomarker for radiation exposure. It should be noted that QPCR on *Klk1* and *Klk1b16* validated the overall strong down-regulation observed for the kallikrein family; however, log_2_-values from microarray and QPCR analysis showed poor correlation for these specific probes–albeit better agreement with deconvolved data. This discrepancy is assumed to result from the different sequence location of respective QPCR assays and microarray probes. The kallikrein family shows high sequence similarity among transcripts and sequence overlap or unspecific binding may thus yield varying results for different assay/probe locations.

Regarding signature gene analysis, the majority of transcripts detected in thyroid data remained undetected in follicular cells and C-cells, i.e. only few specific signature gene transcripts (probes) were detected in both convoluted and deconvolved data. In contrast, all kallikrein transcripts detected in convoluted data were reproduced in at least one, often both, deconvolved data sets. It should be noted that log_2_ ratio values of kallikrein transcripts were generally high, while log_2_ ratio values of IR-associated and TH-responding signature genes were comparatively low with few exceptions. Comparing the change in detection rate upon deconvolution between both signature gene sets and the kallikrein gene set, reproducibility of significant regulation was distinctly higher in the latter set. This observation illustrated that detection rates among weakly responding gene sets were more strongly affected by changes in log_2_ ratio resulting from deconvolution than detection rates among strongly responding gene sets. For biomarker candidates, it is thus desirable to show not only significant up- or down-regulation, but also sufficiently high log_2_ ratio values so that regulation is detectable irrespective of statistical data processing (i.e. in terms of deconvolution), since highly complex tissues may render deconvolution unfeasible.

The detection rate of enriched biological processes was distinctly higher in deconvolved data. This did not only change the intensity but also the biologic quality of the observed overall response: deconvolved data yielded significant regulation in main categories (i.e. *DNA integrity*, *gene expression integrity*, and *stress responses*) that were non-responding in previous analysis of convoluted data [16]. These categories are of particular importance when investigating IR-induced responses. DNA damage and repair are hallmarks of IR-induced effects and false negatives in this category (*DNA integrity*) might lead to erroneous conclusions on e.g. the extent of IR-induced damage, radiation sensitivity, or the timing of DNA repair processes following irradiation. In particular, an absorbed dose of 5.9 Gy over 24 h, even at low dose rate, is expected to result in a DNA damage and repair response to some extent. Lack of regulation in these categories was not in agreement with established knowledge of the dose-response relationship for DNA damage induction. It should be noted that the dose-response relationship is based mostly on *in vitro* data in radiation research, and it is known that the dose-response relationship can differ in the *in vivo* setting. This leads to an analytical dilemma, since two conclusions can be drawn from the disagreement with literature: for one, the damage response in tissues behaves vastly different compared with cultured cell lines; or for the other, data convolution in whole-tissue data masks the damage response due to misrepresentation of individual cell types. Our work is the first to demonstrate that deconvolution is a useful tool to address this dilemma. If deconvolved data would also show no regulatory response for these categories, it would strengthen the conclusion that the damage response *in vivo* is indeed different from the *in vitro* response in this context; however, the detection of damage responses in deconvolved data suggests that the alternative conclusion is more likely.

## Conclusions

Certain findings obtained from convoluted thyroid data were validated upon deconvolution, such as distinct regulation of the kallikrein gene family and a dominating trend of TH-associated signature genes over IR-associated signature genes. Positive responses in biological processes were also validated in deconvolved data. These results demonstrated the benefit of a computational method to assess robustness of results obtained from convoluted expression microarray data. Moreover, deconvolution also revealed responses in important radiation-associated biological processes that remained undetected in mixed cell type data. Deconvolution is a useful tool to validate biomarker screening in mixed cell type data and can reduce the risk of erroneous conclusion from false negatives.

## Supporting information

S1 Fig Impact of cell frequency heatmap. (TIF)

S1 File NC3Rs ARRIVE guidelines checklist. (PDF)

S1 Table QPCR validation of microarray data. (PDF)

S2 Table List of significantly regulated transcripts in thyroid microarray data. (PDF)

S3 Table List of significantly regulated transcripts in deconvolved data for follicular cells. (PDF)

S4 Table List of significantly regulated transcripts in deconvolved data for C-cells. (PDF)

S5 Table Significantly regulated kallikrein transcripts. (PDF)

S6 Table IR associated gene signature for higher and lower C-cell frequency estimates. (PDF)

S7 Table TH-responding gene signature for higher and lower C-cell frequency estimates. (PDF)
