# Supplementary figures and images for: Deconvolution of expression microarray data reveals 131I-induced responses otherwise undetected in thyroid tissue
Source: PLoS One. 2018 Jul 12;13(7):e0197911. doi: 10.1371/journal.pone.0197911 (PMC6042689; doi:10.1371/journal.pone.0197911)

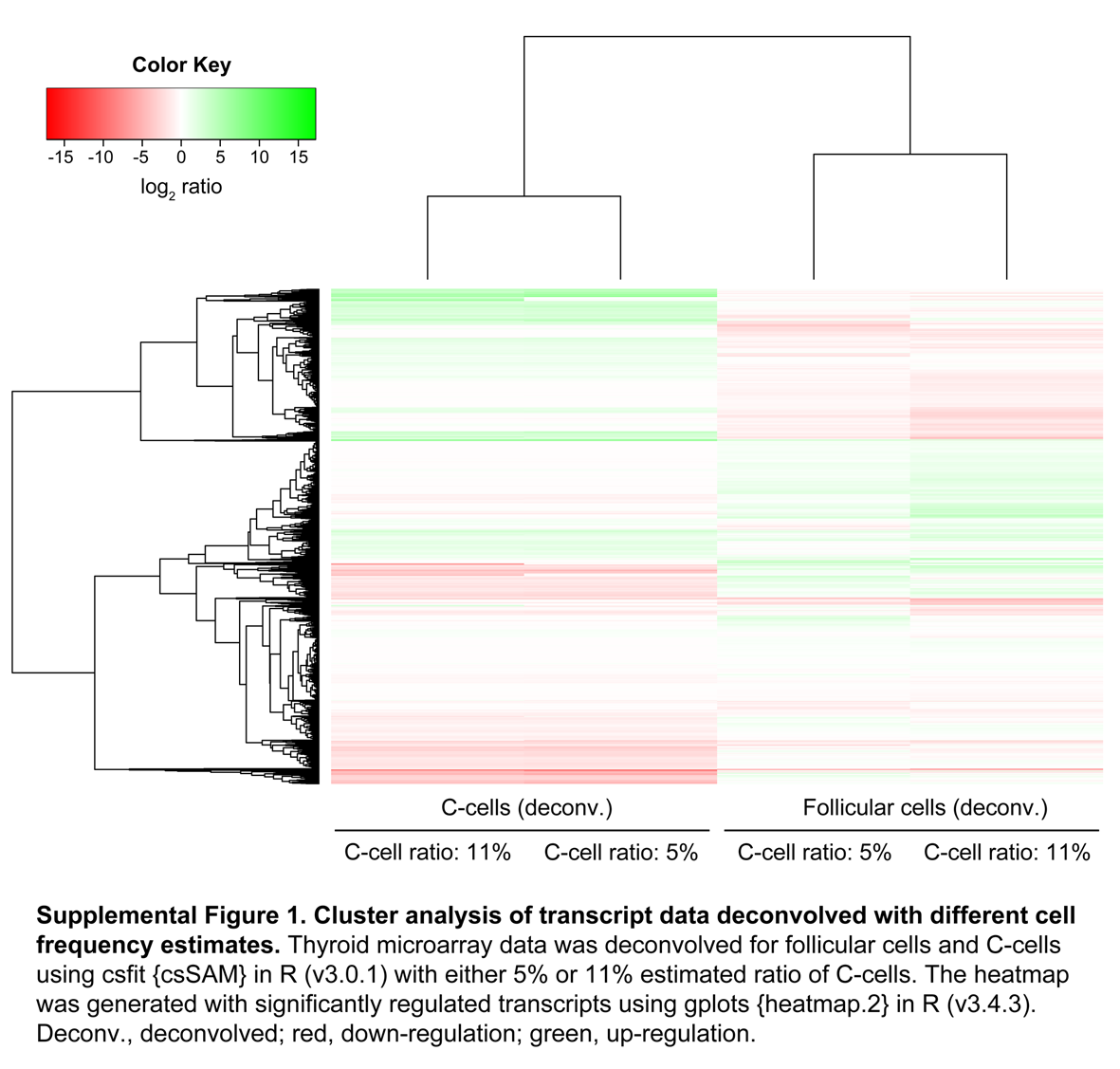

Supplement: S1 Fig — (TIF) [file pone.0197911.s001.tif]
