# Supplementary material for: Deconvolution of expression microarray data reveals 131I-induced responses otherwise undetected in thyroid tissue
Source: PLoS One. 2018 Jul 12;13(7):e0197911. doi: 10.1371/journal.pone.0197911 (PMC6042689; doi:10.1371/journal.pone.0197911)
Supplement: S1 Table — (PDF) [file pone.0197911.s003.pdf]

Supplemental Table 1. QPCR validation of microarray data

		Differential regulation in thyroid after 24 h in response to 5.9 Gy from ^{131}I compared with untreated control	
**Target gene**	**Comment**	**QPCR** *(thyroid cDNA)* $\log_2$	**Microarray data** *(thyroid total RNA)* $\log_2$
Atp2a1	different probe locations[†]	8.5	5.3
Ccnd1	validated	-1.1	-0.92
Ccng1	validated	0.89	0.81
Klk1b16	different probe locations[‡]	-7.1	-5.7
Myh2	validated	4.3	4.3
Pck1	validated	1.8	1.2
Pvalb	validated	4.1	5.2
Tpm2	validated	5.7	4.5

Reference genes were chosen from microarray data that showed lowest overall transcriptional variation (i.e. *Cyp11b1*. *Rptn*. and *Prg3*).

[†]QPCR assay at location 1248; microarray probe at location 3361

[‡]QPCR assay at location 1051; microarray probe at location 1126
