# Supplementary material for: Deconvolution of expression microarray data reveals 131I-induced responses otherwise undetected in thyroid tissue
Source: PLoS One. 2018 Jul 12;13(7):e0197911. doi: 10.1371/journal.pone.0197911 (PMC6042689; doi:10.1371/journal.pone.0197911)
Supplement: S2 Table — (PDF) [file pone.0197911.s004.pdf]

Supplemental Table 2. List of significantly regulated transcripts in thyroid microarray data

The log2-ratio threshold was set to 0.58 with a p-value threshold of 0.01.
Multiple test correction for FDR was performed using the Benjamini Hochberg method in NEXUS 3.0 (BioDiscovery; El Segundo, CA, USA).
The pool size for intensity based pooling was set to 200.

Gene Symbol	Probe	Transcript	Log2 ratio	adjusted p-value
Atp2a1	ILMN_2666864	ILMN_216061	5.35	0.0000
Tnnc2	ILMN_2882658	ILMN_188936	5.32	0.0000
Acta1	ILMN_2738825	ILMN_221671	5.25	0.0000
Pvalb	ILMN_1218223	ILMN_213948	5.24	0.0000
Tnni2	ILMN_2481133	ILMN_191670	5.18	0.0000
Mylpf	ILMN_2977331	ILMN_222158	5.05	0.0000
Mb	ILMN_2954987	ILMN_210416	4.76	0.0000
Tpm2	ILMN_2482209	ILMN_191790	4.53	0.0000
Tnnt3	ILMN_2469018	ILMN_190311	4.51	0.0000
Cox6a2	ILMN_2629581	ILMN_212725	4.48	0.0000
Myh4	ILMN_1241214	ILMN_229231	4.26	0.0000
Myh2	ILMN_1219423	ILMN_219104	4.26	0.0000
Ckm	ILMN_2608804	ILMN_210754	4.26	0.0000
Ttn	ILMN_2416670	ILMN_184200	4.19	0.0000
Ttn	ILMN_3128792	ILMN_255016	4.18	0.0000
Rpl3l	ILMN_2649810	ILMN_214607	4.10	0.0000
Ckmt2	ILMN_2817864	ILMN_218610	4.01	0.0000
Tcap	ILMN_2933357	ILMN_218508	3.92	0.0000
Mb	ILMN_1234662	ILMN_210416	3.91	0.0000
Eno3	ILMN_2757569	ILMN_222979	3.91	0.0000
Pgam2	ILMN_2588815	ILMN_208713	3.90	0.0000
Myh1	ILMN_2865744	ILMN_239496	3.85	0.0000
Ckmt2	ILMN_2698052	ILMN_218610	3.82	0.0000
Mybpc2	ILMN_2676012	ILMN_242030	3.69	0.0000
Eef1a2	ILMN_2971559	ILMN_214177	3.65	0.0000
Actn3	ILMN_2747543	ILMN_219398	3.64	0.0000
Fhl1	ILMN_3117381	ILMN_245416	3.42	0.0000
Fhl1	ILMN_2713285	ILMN_240873	3.39	0.0000
Myoz1	ILMN_2615250	ILMN_211382	3.35	0.0000
Mlf1	ILMN_3112219	ILMN_246637	3.32	0.0000
Ryr1	ILMN_2733073	ILMN_226638	3.27	0.0000
Eef1a2	ILMN_2666990	ILMN_214177	3.26	0.0000
Csrp3	ILMN_2789650	ILMN_217987	3.26	0.0000
Actn2	ILMN_2797061	ILMN_223487	3.25	0.0000
Des	ILMN_2836982	ILMN_223243	3.20	0.0000
Actn3	ILMN_3008110	ILMN_219398	3.20	0.0000
8030451F13Rik	ILMN_2744469	ILMN_256951	3.14	0.0000
Ryr1	ILMN_2730425	ILMN_226638	3.12	0.0000
Tmod4	ILMN_2488846	ILMN_228148	3.07	0.0000
Hrc	ILMN_1259206	ILMN_221655	3.05	0.0000
Myom1	ILMN_2815138	ILMN_241505	3.00	0.0000
Ampd1	ILMN_1237871	ILMN_259430	2.99	0.0000

Tmod4	ILMN_2519313	ILMN_228148	2.96	0.0000
Ryr1	ILMN_2842357	ILMN_226638	2.95	0.0000
2310002L09Rik	ILMN_1214119	ILMN_215549	2.94	0.0000
Csrp3	ILMN_2742068	ILMN_217987	2.94	0.0000
H19	ILMN_2906728	ILMN_219881	2.81	0.0000
Myl1	ILMN_2694907	ILMN_218373	2.79	0.0000
8030451F13Rik	ILMN_2969105	ILMN_256951	2.78	0.0000
Nrap	ILMN_1235070	ILMN_217102	2.77	0.0000
Hspb6	ILMN_2805339	ILMN_234898	2.75	0.0000
Mef2c	ILMN_1214950	ILMN_235198	2.67	0.0000
Fxyd6	ILMN_2609998	ILMN_210866	2.62	0.0000
Myl1	ILMN_2878542	ILMN_218373	2.60	0.0000
Asb2	ILMN_2765759	ILMN_223558	2.58	0.0000
Smpx	ILMN_2785679	ILMN_208970	2.54	0.0000
A2bp1	ILMN_1238309	ILMN_216899	2.46	0.0000
Ampd1	ILMN_2971142	ILMN_259430	2.45	0.0000
Pkia	ILMN_2674122	ILMN_216692	2.43	0.0000
Csrp3	ILMN_2789651	ILMN_217987	2.41	0.0000
Myh1	ILMN_2740259	ILMN_239496	2.39	0.0000
Myoz2	ILMN_1234857	ILMN_223119	2.38	0.0000
Tmod4	ILMN_2438793	ILMN_228148	2.37	0.0000
Rtn2	ILMN_1250904	ILMN_213030	2.36	0.0000
Pde4dip	ILMN_3064283	ILMN_228537	2.33	0.0000
Mlf1	ILMN_2658425	ILMN_215347	2.30	0.0000
Trim72	ILMN_2900484	ILMN_252980	2.28	0.0001
Cryab	ILMN_2840213	ILMN_217805	2.23	0.0000
Pdlim5	ILMN_3137570	ILMN_235674	2.23	0.0000
Ntrk2	ILMN_3138904	ILMN_259664	2.22	0.0000
Schip1	ILMN_2937320	ILMN_216237	2.22	0.0000
Adssl1	ILMN_1245079	ILMN_210075	2.22	0.0000
Mat2a	ILMN_2978838	ILMN_261868	2.16	0.0000
Adssl1	ILMN_2958099	ILMN_210075	2.14	0.0000
Scn4b	ILMN_2813547	ILMN_255776	2.13	0.0000
LOC669660	ILMN_2771219	ILMN_260938	2.09	0.0000
Abcb4	ILMN_2648742	ILMN_214510	2.08	0.0000
Cacna2d1	ILMN_2752030	ILMN_222592	2.07	0.0000
Fsd2	ILMN_1242790	ILMN_211390	2.00	0.0000
Gpc1	ILMN_2635784	ILMN_244157	1.99	0.0000
2310042D19Rik	ILMN_2957987	ILMN_217547	1.95	0.0000
Tmem38a	ILMN_1245673	ILMN_212903	1.94	0.0000
Kcnc4	ILMN_2771095	ILMN_215081	1.93	0.0000
Sln	ILMN_2918875	ILMN_215725	1.93	0.0000
Sypl2	ILMN_2625299	ILMN_212339	1.92	0.0000
Neurl	ILMN_2625940	ILMN_212397	1.90	0.0000
Epdr1	ILMN_1225494	ILMN_214840	1.90	0.0000
1300013J15Rik	ILMN_2849016	ILMN_210868	1.89	0.0000
AI595366	ILMN_3161863	ILMN_253609	1.88	0.0000
Mustn1	ILMN_2797928	ILMN_215350	1.86	0.0000
8430408G22Rik	ILMN_2894211	ILMN_218091	1.85	0.0000
Ky	ILMN_2906855	ILMN_225797	1.84	0.0000

Pdzrn3	ILMN_3156010	ILMN_214528	1.82	0.0000
Fbxo32	ILMN_2873444	ILMN_219973	1.81	0.0000
Dtna	ILMN_1221805	ILMN_223037	1.80	0.0000
Ak1	ILMN_2667805	ILMN_251058	1.80	0.0000
Dmn	ILMN_1214880	ILMN_250370	1.74	0.0000
Pfkm	ILMN_3004553	ILMN_208909	1.72	0.0000
Tmem100	ILMN_1224014	ILMN_223572	1.71	0.0000
Pitx2	ILMN_3118071	ILMN_237162	1.68	0.0000
Kif1b	ILMN_3034877	ILMN_207470	1.68	0.0000
Camk2b	ILMN_1236009	ILMN_223176	1.67	0.0000
Pfn2	ILMN_2929791	ILMN_210891	1.67	0.0069
Rilpl1	ILMN_2848163	ILMN_218974	1.66	0.0000
Unc45b	ILMN_3153940	ILMN_221227	1.65	0.0000
Pop5	ILMN_2973925	ILMN_213331	1.62	0.0000
Gmpr	ILMN_2602581	ILMN_210136	1.60	0.0004
Mustn1	ILMN_2658461	ILMN_215350	1.59	0.0000
Rcsd1	ILMN_1250704	ILMN_214047	1.58	0.0000
Scgb1a1	ILMN_2749152	ILMN_222406	1.57	0.0000
Ank1	ILMN_2715601	ILMN_208704	1.56	0.0000
LOC100046690	ILMN_1236869	ILMN_312512	1.55	0.0000
Kng1	ILMN_2788223	ILMN_214717	1.55	0.0000
Ptp4a3	ILMN_2655260	ILMN_213418	1.54	0.0000
Ddit4l	ILMN_2695819	ILMN_218436	1.53	0.0004
Pfn2	ILMN_1260064	ILMN_210891	1.52	0.0000
LOC100044934	ILMN_2732747	ILMN_318532	1.52	0.0000
Mrpl37	ILMN_1254715	ILMN_221393	1.51	0.0000
Mef2c	ILMN_2732465	ILMN_235198	1.47	0.0003
Pfkm	ILMN_2628892	ILMN_208909	1.47	0.0000
Dmn	ILMN_2636266	ILMN_250370	1.46	0.0000
Tuba8	ILMN_1221298	ILMN_248050	1.44	0.0000
Cyp2b10	ILMN_2594926	ILMN_230152	1.44	0.0000
Gstp2	ILMN_2917386	ILMN_258260	1.42	0.0004
Capzb	ILMN_3031009	ILMN_253743	1.42	0.0000
LOC100046690	ILMN_1244879	ILMN_312512	1.41	0.0000
Pdlim3	ILMN_1235230	ILMN_213954	1.41	0.0000
Phtf2	ILMN_1252206	ILMN_218550	1.41	0.0000
Rbm38	ILMN_1240979	ILMN_210582	1.40	0.0000
Wfdc1	ILMN_2466164	ILMN_189984	1.37	0.0000
Pdlim3	ILMN_2885990	ILMN_213954	1.36	0.0000
Aqp1	ILMN_2980661	ILMN_216314	1.35	0.0000
Mef2c	ILMN_2887992	ILMN_235198	1.34	0.0000
Aqp1	ILMN_2756486	ILMN_216314	1.34	0.0000
Lynx1	ILMN_1256369	ILMN_218793	1.33	0.0004
Art3	ILMN_1223147	ILMN_210398	1.29	0.0003
Popdc2	ILMN_3128351	ILMN_211242	1.29	0.0000
Suclg1	ILMN_2683681	ILMN_217480	1.29	0.0000
Tbx1	ILMN_1252399	ILMN_226888	1.27	0.0002
Kng2	ILMN_3126522	ILMN_199376	1.27	0.0000
Cpt1b	ILMN_2709240	ILMN_216493	1.26	0.0000
Atp1a2	ILMN_2722716	ILMN_220489	1.26	0.0000

Popdc3	ILMN_2895568	ILMN_210556	1.25	0.0000
Reep1	ILMN_2791644	ILMN_218368	1.25	0.0000
2310003L22Rik	ILMN_1216813	ILMN_222302	1.25	0.0035
Art3	ILMN_2836607	ILMN_210398	1.23	0.0000
Olig1	ILMN_2760105	ILMN_223162	1.23	0.0000
Slc27a2	ILMN_2648445	ILMN_258646	1.23	0.0081
Tmem45b	ILMN_2661366	ILMN_215591	1.22	0.0000
Acin1	ILMN_2755195	ILMN_222814	1.21	0.0001
EG277333	ILMN_2591127	ILMN_315488	1.21	0.0000
Tcea3	ILMN_2907214	ILMN_243591	1.21	0.0000
Rtn4	ILMN_3140071	ILMN_216522	1.20	0.0000
Hoxa5	ILMN_1217978	ILMN_213378	1.20	0.0000
Fabp4	ILMN_2615947	ILMN_211442	1.19	0.0000
Ndrg2	ILMN_3001650	ILMN_244033	1.18	0.0001
Hbb-b1	ILMN_1235372	ILMN_212995	1.18	0.0001
B230339M05Rik	ILMN_2894574	ILMN_260273	1.18	0.0001
Clpx	ILMN_3154849	ILMN_211234	1.18	0.0000
Pck1	ILMN_1213632	ILMN_238641	1.17	0.0001
Lyve1	ILMN_2483304	ILMN_191911	1.17	0.0000
Sdhb	ILMN_2592389	ILMN_209086	1.17	0.0002
Car14	ILMN_2973824	ILMN_208706	1.16	0.0000
Mkks	ILMN_1257332	ILMN_217710	1.16	0.0000
Rtn4	ILMN_2699645	ILMN_216522	1.15	0.0000
Vldlr	ILMN_1218264	ILMN_188056	1.15	0.0000
Srpk3	ILMN_2684515	ILMN_217542	1.15	0.0000
Fabp3	ILMN_2887630	ILMN_210798	1.14	0.0000
A930009M04Rik	ILMN_2734855	ILMN_189339	1.13	0.0000
Vldlr	ILMN_2515601	ILMN_188056	1.13	0.0000
Asah3l	ILMN_2629112	ILMN_212677	1.13	0.0000
Twf2	ILMN_1234388	ILMN_212893	1.13	0.0000
Alas2	ILMN_2675874	ILMN_216832	1.12	0.0002
Acadl	ILMN_2735084	ILMN_217932	1.12	0.0000
Angptl4	ILMN_2759365	ILMN_259520	1.12	0.0000
Vldlr	ILMN_2796472	ILMN_188056	1.12	0.0000
D10Jhu81e	ILMN_2991545	ILMN_223114	1.11	0.0000
Ahnak	ILMN_1258578	ILMN_253782	1.09	0.0001
Pparg	ILMN_1221594	ILMN_212393	1.09	0.0004
Aqp7	ILMN_2943165	ILMN_210666	1.09	0.0000
Gyg	ILMN_2686069	ILMN_215615	1.09	0.0000
Igf2	ILMN_2597769	ILMN_209642	1.08	0.0034
2310076L09Rik	ILMN_3158565	ILMN_210594	1.08	0.0001
Pcolce2	ILMN_1238603	ILMN_211112	1.08	0.0037
Coq6	ILMN_2766720	ILMN_211735	1.07	0.0001
Tmod1	ILMN_2977404	ILMN_189256	1.07	0.0000
Cd274	ILMN_1226800	ILMN_208897	1.07	0.0003
Hoxb4	ILMN_1241077	ILMN_231624	1.07	0.0000
Itih4	ILMN_2718431	ILMN_212489	1.07	0.0055
Rbm38	ILMN_2926155	ILMN_210582	1.07	0.0002
2310003L22Rik	ILMN_2747677	ILMN_222302	1.06	0.0000
Cox7a1	ILMN_1240857	ILMN_215598	1.06	0.0000

Acot11	ILMN_2799351	ILMN_217575	1.05	0.0007
Etfa	ILMN_2649966	ILMN_214618	1.05	0.0000
Gpc3	ILMN_2832979	ILMN_220269	1.05	0.0064
Lrrc39	ILMN_1230043	ILMN_218323	1.05	0.0002
St3gal3	ILMN_2879848	ILMN_211800	1.05	0.0005
Hspb1	ILMN_2706269	ILMN_224679	1.03	0.0012
Zeb1	ILMN_2492500	ILMN_232490	1.03	0.0001
Vdac1	ILMN_2832808	ILMN_237906	1.03	0.0003
Adora1	ILMN_3143358	ILMN_238585	1.02	0.0039
Sgcb	ILMN_1222716	ILMN_209284	1.02	0.0002
Aldh1a1	ILMN_1222734	ILMN_261983	1.02	0.0006
Ntrk2	ILMN_2705460	ILMN_240641	1.01	0.0004
Crls1	ILMN_1219797	ILMN_260069	1.01	0.0004
Gpx3	ILMN_2715546	ILMN_219947	1.01	0.0001
Grb14	ILMN_2625280	ILMN_212337	1.01	0.0063
EG317677	ILMN_2929594	ILMN_213670	1.00	0.0000
Aldoa	ILMN_2599130	ILMN_209778	1.00	0.0000
Maob	ILMN_2719069	ILMN_211647	1.00	0.0017
Cdsn	ILMN_2796382	ILMN_229304	1.00	0.0001
Aqp1	ILMN_2980663	ILMN_216314	1.00	0.0024
Mtap7d1	ILMN_2855590	ILMN_219950	0.99	0.0001
Aqp4	ILMN_2757232	ILMN_247494	0.99	0.0000
Tnxb	ILMN_2440823	ILMN_187061	0.99	0.0059
Ppara	ILMN_2927172	ILMN_221142	0.99	0.0009
Acaa2	ILMN_2704823	ILMN_254698	0.99	0.0001
Egfl7	ILMN_3163288	ILMN_257108	0.99	0.0007
Otop1	ILMN_1228647	ILMN_214803	0.98	0.0001
St3gal6	ILMN_2695199	ILMN_230085	0.98	0.0000
Letm1	ILMN_2755152	ILMN_211496	0.98	0.0001
V1rd6	ILMN_2971043	ILMN_194381	0.98	0.0000
Npr3	ILMN_3127391	ILMN_243188	0.98	0.0000
Hbb-b2	ILMN_2729513	ILMN_220984	0.97	0.0026
Kng1	ILMN_2788221	ILMN_214717	0.97	0.0008
LOC620807	ILMN_2953807	ILMN_234960	0.97	0.0015
Xdh	ILMN_1239055	ILMN_246330	0.97	0.0010
Cpt1b	ILMN_1227666	ILMN_216493	0.97	0.0006
Idh3g	ILMN_1224368	ILMN_213852	0.96	0.0001
Mgst3	ILMN_1238479	ILMN_210134	0.96	0.0001
Acadl	ILMN_2783519	ILMN_217932	0.96	0.0010
Mgst3	ILMN_2615035	ILMN_210134	0.96	0.0001
Letmd1	ILMN_2616215	ILMN_211469	0.96	0.0051
Ppp1r1a	ILMN_2616584	ILMN_211515	0.95	0.0001
Atp5j2	ILMN_1213914	ILMN_222082	0.95	0.0000
Sgk3	ILMN_3108894	ILMN_226965	0.94	0.0000
Clstn3	ILMN_2827217	ILMN_209442	0.94	0.0022
Pparg	ILMN_1221060	ILMN_212393	0.94	0.0017
AI427515	ILMN_1226356	ILMN_223744	0.94	0.0001
Acaa2	ILMN_2704826	ILMN_211418	0.94	0.0004
Otop1	ILMN_3137899	ILMN_214803	0.94	0.0003
Atp5k	ILMN_2776899	ILMN_224224	0.94	0.0072

Macrod1	ILMN_2683986	ILMN_217502	0.93	0.0001
Acaa2	ILMN_2704822	ILMN_211418	0.93	0.0004
Acsm3	ILMN_3111685	ILMN_249319	0.93	0.0038
Gbp3	ILMN_2918002	ILMN_216816	0.93	0.0036
Kcnk3	ILMN_2669487	ILMN_259977	0.92	0.0008
Vim	ILMN_2451022	ILMN_187199	0.92	0.0030
2810055F11Rik	ILMN_1230505	ILMN_213841	0.92	0.0004
Itga11	ILMN_2701778	ILMN_210182	0.92	0.0043
S100a13	ILMN_2987984	ILMN_218915	0.92	0.0005
Rab6b	ILMN_2761430	ILMN_223251	0.92	0.0002
Cd36	ILMN_1232470	ILMN_210534	0.92	0.0007
Ly6c1	ILMN_1254927	ILMN_234229	0.92	0.0025
Tle4	ILMN_2589792	ILMN_208815	0.92	0.0006
Aqp7	ILMN_1254953	ILMN_210666	0.91	0.0007
Lipe	ILMN_2742636	ILMN_221959	0.91	0.0000
Ndufb2	ILMN_2636435	ILMN_226970	0.91	0.0002
Etfdh	ILMN_3005058	ILMN_216328	0.91	0.0001
Ugp2	ILMN_1244631	ILMN_193899	0.91	0.0033
Ndufb5	ILMN_2617335	ILMN_211581	0.90	0.0002
2310076L09Rik	ILMN_2607215	ILMN_210594	0.90	0.0021
LOC668837	ILMN_2587859	ILMN_312076	0.90	0.0010
Cenpa	ILMN_1236574	ILMN_223574	0.90	0.0034
Eif4e3	ILMN_1254878	ILMN_211293	0.90	0.0002
Cidea	ILMN_1215446	ILMN_221565	0.89	0.0004
Rusc2	ILMN_1214953	ILMN_224812	0.89	0.0009
Ecm1	ILMN_2947526	ILMN_213475	0.89	0.0003
Tmem14c	ILMN_2472110	ILMN_186398	0.89	0.0000
Rasl2-9	ILMN_1229223	ILMN_219836	0.89	0.0004
Gbp3	ILMN_1244513	ILMN_216816	0.89	0.0005
Dpt	ILMN_2606210	ILMN_210496	0.89	0.0004
Hba-a1	ILMN_1212702	ILMN_216083	0.89	0.0001
Myo5b	ILMN_2610576	ILMN_210919	0.89	0.0054
Stbd1	ILMN_2663211	ILMN_215752	0.89	0.0057
Rgs5	ILMN_2979824	ILMN_256631	0.89	0.0033
Usmg5	ILMN_1219002	ILMN_194875	0.88	0.0074
Ubac1	ILMN_1241976	ILMN_185910	0.88	0.0027
Gpd2	ILMN_1215797	ILMN_214592	0.88	0.0002
Phlda3	ILMN_2923607	ILMN_212624	0.88	0.0042
Acaa2	ILMN_2788984	ILMN_254698	0.87	0.0017
Sftpc	ILMN_2638865	ILMN_213605	0.87	0.0002
Eif4e3	ILMN_2643291	ILMN_211293	0.87	0.0016
Dci	ILMN_2615207	ILMN_211377	0.87	0.0001
Cdh13	ILMN_2592321	ILMN_228863	0.86	0.0006
Hdc	ILMN_2965903	ILMN_242858	0.86	0.0046
Hp	ILMN_2668510	ILMN_214144	0.86	0.0076
D0H4S114	ILMN_2680054	ILMN_217188	0.86	0.0019
Akap1	ILMN_3146196	ILMN_234975	0.86	0.0016
Slc25a4	ILMN_1225312	ILMN_249346	0.86	0.0000
Lgals9	ILMN_2752873	ILMN_220025	0.86	0.0060
Tmem65	ILMN_2987564	ILMN_255403	0.86	0.0001

Glul	ILMN_2644496	ILMN_208631	0.86	0.0036
Pkig	ILMN_3090728	ILMN_230872	0.86	0.0018
Mdh2	ILMN_2873112	ILMN_223596	0.86	0.0029
Sh3rf2	ILMN_2942551	ILMN_213769	0.85	0.0000
Sdpr	ILMN_2687547	ILMN_217783	0.85	0.0005
Dbp	ILMN_2616226	ILMN_211471	0.85	0.0002
Cib2	ILMN_2659168	ILMN_215411	0.85	0.0049
Qrsl1	ILMN_1214450	ILMN_230382	0.85	0.0007
A130092J06Rik	ILMN_2601453	ILMN_210026	0.85	0.0007
Zadh1	ILMN_2476885	ILMN_191185	0.85	0.0087
Lor	ILMN_1241825	ILMN_218790	0.84	0.0005
Mreg	ILMN_2976159	ILMN_253687	0.84	0.0000
Ebf2	ILMN_2883606	ILMN_221447	0.84	0.0092
Txn2	ILMN_1257624	ILMN_192993	0.84	0.0010
Optn	ILMN_2907370	ILMN_216896	0.84	0.0083
Lims2	ILMN_2738345	ILMN_221324	0.84	0.0005
Cd209d	ILMN_2696291	ILMN_218474	0.84	0.0005
Pkm2	ILMN_2756041	ILMN_222874	0.84	0.0051
Srgn	ILMN_2722732	ILMN_220490	0.84	0.0056
Acadvl	ILMN_2956932	ILMN_249043	0.84	0.0003
Slc25a19	ILMN_1237074	ILMN_211007	0.84	0.0015
Acat2	ILMN_1253008	ILMN_217761	0.83	0.0089
Echdc3	ILMN_1234901	ILMN_211941	0.83	0.0005
Rnf125	ILMN_2917338	ILMN_209261	0.83	0.0011
Pdhb	ILMN_3041839	ILMN_213240	0.83	0.0007
LOC100047606	ILMN_2675199	ILMN_317392	0.83	0.0074
Adora1	ILMN_1254016	ILMN_238585	0.83	0.0002
Hp	ILMN_2668509	ILMN_214144	0.83	0.0047
Tppp3	ILMN_2655929	ILMN_215136	0.83	0.0000
Ndufa8	ILMN_2682019	ILMN_217333	0.82	0.0018
Sdcbp2	ILMN_2825446	ILMN_217512	0.82	0.0022
Sgcb	ILMN_1255256	ILMN_209284	0.82	0.0083
Tubb6	ILMN_2718217	ILMN_220148	0.82	0.0055
EG622339	ILMN_3006990	ILMN_232532	0.82	0.0006
Cox7b	ILMN_2671689	ILMN_242680	0.82	0.0009
Itih4	ILMN_2797276	ILMN_212489	0.81	0.0016
Ndufb4	ILMN_1227012	ILMN_223783	0.81	0.0007
Tmem70	ILMN_3153614	ILMN_217035	0.81	0.0008
Ramp1	ILMN_2734391	ILMN_221353	0.81	0.0016
Ccng1	ILMN_2500276	ILMN_232357	0.81	0.0076
Cox8b	ILMN_2727520	ILMN_220835	0.81	0.0007
Lrtm1	ILMN_1241151	ILMN_209217	0.80	0.0052
Prkag2	ILMN_3161626	ILMN_221101	0.80	0.0002
2810453I06Rik	ILMN_1224076	ILMN_244463	0.80	0.0091
Nol3	ILMN_2931033	ILMN_213608	0.80	0.0091
Nox4	ILMN_2698499	ILMN_218644	0.80	0.0003
Cldn5	ILMN_1241293	ILMN_224508	0.80	0.0085
Dst	ILMN_2721385	ILMN_211736	0.80	0.0037
Cisd1	ILMN_2706205	ILMN_219251	0.80	0.0012
Mrpl1	ILMN_2711966	ILMN_219665	0.80	0.0016

Aoc3	ILMN_2625920	ILMN_212395	0.79	0.0019
Pi16	ILMN_2669062	ILMN_216258	0.79	0.0031
Tnnc1	ILMN_2503052	ILMN_194064	0.79	0.0056
Nrp1	ILMN_1237197	ILMN_214296	0.79	0.0017
Ubac1	ILMN_2431046	ILMN_185910	0.79	0.0010
Cyp27a1	ILMN_2960114	ILMN_211874	0.79	0.0018
Phlda3	ILMN_2628567	ILMN_212624	0.79	0.0011
Sdhd	ILMN_1239143	ILMN_209676	0.79	0.0040
Nampt	ILMN_2821850	ILMN_212748	0.79	0.0018
Idh3a	ILMN_2740134	ILMN_221774	0.79	0.0087
Scgb3a2	ILMN_2753867	ILMN_222722	0.79	0.0002
Ndufb7	ILMN_2717460	ILMN_220088	0.78	0.0010
Tsc22d3	ILMN_3150811	ILMN_257708	0.77	0.0024
Atpaf2	ILMN_2601118	ILMN_196566	0.77	0.0015
Rsph1	ILMN_2441534	ILMN_187143	0.77	0.0000
Ndufa3	ILMN_2792560	ILMN_261570	0.77	0.0002
Nrp1	ILMN_2669912	ILMN_214296	0.77	0.0008
Hyal1	ILMN_2644375	ILMN_214111	0.77	0.0016
Tspan3	ILMN_1232099	ILMN_189492	0.76	0.0010
4833442J19Rik	ILMN_3160574	ILMN_214657	0.76	0.0098
Hmgcs2	ILMN_1216322	ILMN_219260	0.76	0.0069
LOC100046918	ILMN_2699167	ILMN_318141	0.76	0.0068
D430015B01Rik	ILMN_1224427	ILMN_254424	0.75	0.0078
Fmo1	ILMN_2838308	ILMN_213755	0.75	0.0031
Fbxo31	ILMN_2687032	ILMN_217743	0.75	0.0038
3300001G02Rik	ILMN_2990229	ILMN_240673	0.75	0.0057
Pex7	ILMN_2739118	ILMN_221690	0.75	0.0025
Lias	ILMN_2743668	ILMN_221016	0.75	0.0007
Serping1	ILMN_2913166	ILMN_256713	0.75	0.0037
Etfdh	ILMN_2669804	ILMN_216328	0.75	0.0073
Cycs	ILMN_3155380	ILMN_223048	0.75	0.0038
Pcolce2	ILMN_2678421	ILMN_211112	0.74	0.0089
BC028528	ILMN_2656422	ILMN_215178	0.74	0.0035
Cxcl13	ILMN_2760019	ILMN_223155	0.74	0.0090
Pgm2	ILMN_2788673	ILMN_225618	0.74	0.0048
Coq6	ILMN_1229589	ILMN_211735	0.74	0.0021
Esam1	ILMN_2690839	ILMN_218041	0.74	0.0053
1810049H13Rik	ILMN_2680665	ILMN_217236	0.74	0.0019
Ndufa4	ILMN_2666061	ILMN_215988	0.74	0.0018
Plin	ILMN_2687984	ILMN_217822	0.74	0.0061
Aldh5a1	ILMN_2664660	ILMN_246401	0.73	0.0066
D10Ertd322e	ILMN_2727992	ILMN_220871	0.73	0.0018
Rnf144a	ILMN_2678127	ILMN_217013	0.73	0.0033
Arsa	ILMN_1225552	ILMN_254441	0.73	0.0098
Ndufb3	ILMN_2734511	ILMN_221363	0.73	0.0015
Gypc	ILMN_1218358	ILMN_260598	0.73	0.0089
Acads	ILMN_2806676	ILMN_210961	0.73	0.0030
Sftpd	ILMN_1244169	ILMN_214890	0.73	0.0004
Cox11	ILMN_1247397	ILMN_222359	0.72	0.0048
Mtch2	ILMN_1229238	ILMN_210887	0.72	0.0076

Ednrb	ILMN_2589640	ILMN_208799	0.72	0.0047
Sucla2	ILMN_2596942	ILMN_241666	0.72	0.0079
Sepp1	ILMN_3141048	ILMN_217349	0.72	0.0086
Lace1	ILMN_1231795	ILMN_213918	0.72	0.0066
Igfbp6	ILMN_2689790	ILMN_217958	0.72	0.0047
Reep6	ILMN_2634216	ILMN_213154	0.72	0.0071
Hsd17b7	ILMN_1229529	ILMN_216414	0.72	0.0037
Suclg1	ILMN_1216493	ILMN_217480	0.71	0.0005
Agpat6	ILMN_1252335	ILMN_213262	0.71	0.0024
Tpi1	ILMN_2932359	ILMN_186788	0.71	0.0011
1810020D17Rik	ILMN_2896944	ILMN_223656	0.71	0.0096
Cd300lg	ILMN_2688416	ILMN_252644	0.71	0.0034
1700037H04Rik	ILMN_1213070	ILMN_232254	0.71	0.0039
Sar1b	ILMN_2997998	ILMN_219902	0.70	0.0090
Rilp	ILMN_1218228	ILMN_226905	0.70	0.0065
9530058B02Rik	ILMN_2904819	ILMN_209043	0.70	0.0082
OTTMUSG00000000971	ILMN_2864309	ILMN_244723	0.69	0.0017
Csda	ILMN_1234336	ILMN_211098	0.69	0.0087
Dlst	ILMN_1237507	ILMN_261671	0.69	0.0040
Hspe1	ILMN_2960308	ILMN_213386	0.69	0.0010
Ramp2	ILMN_2661422	ILMN_215597	0.69	0.0091
Mrps31	ILMN_2829295	ILMN_220521	0.69	0.0011
Nt5e	ILMN_2813830	ILMN_213360	0.69	0.0058
Coq5	ILMN_1257300	ILMN_211122	0.69	0.0091
Hint3	ILMN_2757092	ILMN_222943	0.69	0.0025
Cox6b1	ILMN_2616405	ILMN_211493	0.68	0.0050
Gadd45b	ILMN_2900653	ILMN_223355	0.68	0.0001
Rb1	ILMN_2861644	ILMN_217149	0.68	0.0088
Grpel1	ILMN_2945806	ILMN_216950	0.67	0.0080
Etfa	ILMN_2800655	ILMN_214618	0.67	0.0078
Tgfbr3	ILMN_2789239	ILMN_240795	0.67	0.0024
Pkn1	ILMN_2990872	ILMN_256799	0.67	0.0032
Acadm	ILMN_2810473	ILMN_227083	0.67	0.0047
Etfb	ILMN_3104414	ILMN_212569	0.67	0.0019
Mrps31	ILMN_2732565	ILMN_220521	0.67	0.0032
Nit2	ILMN_2681622	ILMN_217299	0.67	0.0034
Gpd2	ILMN_1247257	ILMN_214592	0.67	0.0082
LOC100047214	ILMN_1255556	ILMN_313723	0.67	0.0073
Actc1	ILMN_2598916	ILMN_209757	0.66	0.0000
2310007A19Rik	ILMN_1222196	ILMN_227146	0.66	0.0032
Krt13	ILMN_2704972	ILMN_230613	0.66	0.0000
Rhot2	ILMN_2740348	ILMN_215732	0.66	0.0093
Mbnl1	ILMN_2766253	ILMN_219259	0.66	0.0097
Ndufs4	ILMN_1234521	ILMN_212301	0.66	0.0049
Clic5	ILMN_2677634	ILMN_216973	0.66	0.0095
Rpl39l	ILMN_2684892	ILMN_217567	0.65	0.0068
Pla2g7	ILMN_2692696	ILMN_218184	0.65	0.0004
Apoc1	ILMN_2599794	ILMN_208882	0.65	0.0071
Rmnd1	ILMN_2955535	ILMN_209646	0.65	0.0042
Abcb1b	ILMN_2918499	ILMN_212008	0.64	0.0025

Evc	ILMN_2706562	ILMN_238803	0.64	0.0005
Uqcrc2	ILMN_2435140	ILMN_186391	0.64	0.0050
LOC100048613	ILMN_2647188	ILMN_311557	0.64	0.0090
Cox5a	ILMN_2682800	ILMN_247252	0.64	0.0035
Krtdap	ILMN_2868152	ILMN_237217	0.63	0.0000
Gpc3	ILMN_2719973	ILMN_220269	0.63	0.0006
Uqcrh	ILMN_2512849	ILMN_195130	0.62	0.0038
Vsig4	ILMN_1241350	ILMN_187127	0.62	0.0001
LOC100047762	ILMN_1217043	ILMN_319923	0.62	0.0011
Uck1	ILMN_1226366	ILMN_189864	0.62	0.0034
Pknox2	ILMN_3135037	ILMN_254436	0.61	0.0000
Sdhb	ILMN_2769065	ILMN_209086	0.61	0.0093
Hamp2	ILMN_2852624	ILMN_217466	0.61	0.0003
Mrps18b	ILMN_2865239	ILMN_252778	0.61	0.0060
Mfn2	ILMN_2641278	ILMN_213835	0.60	0.0057
Gpnmb	ILMN_2648669	ILMN_211323	0.58	0.0004
Tmem139	ILMN_2600822	ILMN_209958	-0.59	0.0001
Arhgef3	ILMN_2645208	ILMN_214179	-0.59	0.0084
Kcnk5	ILMN_1232263	ILMN_218669	-0.59	0.0003
Hist1h2ah	ILMN_2730329	ILMN_196729	-0.61	0.0029
Kcnj15	ILMN_3125966	ILMN_233678	-0.62	0.0012
Dyrk3	ILMN_2699522	ILMN_258582	-0.62	0.0031
Enpp1	ILMN_2782964	ILMN_210635	-0.62	0.0069
Ifi30	ILMN_2767918	ILMN_216051	-0.64	0.0007
Ren1	ILMN_2777226	ILMN_242576	-0.64	0.0098
Bach2	ILMN_2819558	ILMN_223297	-0.64	0.0003
Sh3tc2	ILMN_1240728	ILMN_241633	-0.65	0.0012
Prps2	ILMN_1234614	ILMN_213601	-0.65	0.0058
Rps4x	ILMN_2948086	ILMN_233225	-0.66	0.0061
Rims3	ILMN_2488599	ILMN_192493	-0.66	0.0010
Abcc3	ILMN_2685157	ILMN_245663	-0.66	0.0001
Erp29	ILMN_1239185	ILMN_218519	-0.66	0.0083
Ttpa	ILMN_2470983	ILMN_190534	-0.66	0.0009
Trib2	ILMN_2432550	ILMN_186091	-0.66	0.0082
Flvcr2	ILMN_1248190	ILMN_222509	-0.67	0.0002
Dnajc6	ILMN_1230224	ILMN_247689	-0.67	0.0073
2610528J11Rik	ILMN_1221084	ILMN_216824	-0.67	0.0000
Epha1	ILMN_2972748	ILMN_213132	-0.67	0.0000
B3galt5	ILMN_1245509	ILMN_221459	-0.68	0.0063
Ch25h	ILMN_2702303	ILMN_218948	-0.68	0.0000
Slc9a3r2	ILMN_2710274	ILMN_219545	-0.68	0.0058
Eef1b2	ILMN_1229042	ILMN_212511	-0.69	0.0035
Pi4k2b	ILMN_3099758	ILMN_207467	-0.69	0.0021
Slc7a8	ILMN_2598877	ILMN_209055	-0.69	0.0077
Chad	ILMN_2659340	ILMN_215425	-0.69	0.0010
Cd44	ILMN_3114585	ILMN_245439	-0.69	0.0001
Adh1	ILMN_2850077	ILMN_214816	-0.70	0.0070
Tlr1	ILMN_1236908	ILMN_220747	-0.70	0.0000
Litaf	ILMN_2665490	ILMN_238658	-0.70	0.0030
D14Ertd668e	ILMN_2827780	ILMN_217565	-0.70	0.0012

Ndfip2	ILMN_1247636	ILMN_219034	-0.70	0.0098
B3gnt5	ILMN_1216368	ILMN_222923	-0.70	0.0000
Prr15	ILMN_2649456	ILMN_214576	-0.71	0.0003
Lcn2	ILMN_2712075	ILMN_219674	-0.71	0.0061
Zfhx3	ILMN_2623793	ILMN_249194	-0.71	0.0034
Ndst1	ILMN_2832641	ILMN_253710	-0.71	0.0022
Rps8	ILMN_1234590	ILMN_218934	-0.72	0.0026
Rerg	ILMN_1230648	ILMN_212208	-0.72	0.0001
Echdc2	ILMN_2846194	ILMN_211203	-0.72	0.0039
Slc31a1	ILMN_1254437	ILMN_221784	-0.72	0.0072
Hs3st1	ILMN_1253182	ILMN_209971	-0.73	0.0076
Dgka	ILMN_2940446	ILMN_215394	-0.73	0.0081
Psat1	ILMN_1221048	ILMN_253395	-0.73	0.0022
Luzp1	ILMN_2942276	ILMN_209553	-0.74	0.0045
Pdk3	ILMN_2676876	ILMN_216915	-0.74	0.0000
Upb1	ILMN_2516705	ILMN_192546	-0.74	0.0000
BC030476	ILMN_2819380	ILMN_209600	-0.74	0.0035
Spint1	ILMN_2621463	ILMN_227593	-0.75	0.0016
Sh3bgrl3	ILMN_2625351	ILMN_212344	-0.75	0.0087
Rps26	ILMN_2794676	ILMN_234107	-0.75	0.0010
Tegt	ILMN_2697248	ILMN_218556	-0.75	0.0026
Sphk1	ILMN_1232884	ILMN_255350	-0.76	0.0002
Tjp2	ILMN_1256328	ILMN_249962	-0.76	0.0049
Ywhaz	ILMN_2952914	ILMN_188261	-0.76	0.0035
AI661453	ILMN_2830520	ILMN_214565	-0.76	0.0001
Rin2	ILMN_2663196	ILMN_215751	-0.77	0.0051
Ctnnal1	ILMN_1232261	ILMN_214433	-0.78	0.0056
Stx3	ILMN_3162879	ILMN_258475	-0.78	0.0001
Fkbp4	ILMN_1257428	ILMN_232691	-0.78	0.0038
Slc13a2	ILMN_2646369	ILMN_214285	-0.79	0.0000
Syvn1	ILMN_2628258	ILMN_212597	-0.79	0.0042
Gcnt1	ILMN_2672297	ILMN_214513	-0.79	0.0002
Gna14	ILMN_2597778	ILMN_209643	-0.79	0.0001
Ldb1	ILMN_2601907	ILMN_210069	-0.79	0.0004
Hsp90b1	ILMN_2504433	ILMN_194217	-0.79	0.0028
6330505N24Rik	ILMN_1220688	ILMN_230010	-0.79	0.0043
Elmo1	ILMN_2658407	ILMN_215345	-0.80	0.0050
Mtap7	ILMN_1252545	ILMN_245164	-0.80	0.0026
Cd27	ILMN_3128992	ILMN_230311	-0.80	0.0064
Rnasek	ILMN_2593134	ILMN_209163	-0.81	0.0005
Rpl12	ILMN_2874228	ILMN_232095	-0.81	0.0004
Kcnk6	ILMN_3049650	ILMN_240136	-0.81	0.0027
Cds1	ILMN_2919860	ILMN_215908	-0.82	0.0011
Dusp6	ILMN_2925711	ILMN_213741	-0.82	0.0006
C430004E15Rik	ILMN_2625047	ILMN_212315	-0.82	0.0086
Hist1h2an	ILMN_1248830	ILMN_214020	-0.82	0.0095
Bcl2l2	ILMN_2936517	ILMN_210038	-0.82	0.0052
Tmem117	ILMN_2692733	ILMN_218190	-0.82	0.0016
Mapre2	ILMN_1229743	ILMN_217280	-0.83	0.0008
Gas6	ILMN_2686327	ILMN_217682	-0.83	0.0021

Upb1	ILMN_2870786	ILMN_192546	-0.83	0.0000
Sorl1	ILMN_1249578	ILMN_224800	-0.84	0.0031
Qsox1	ILMN_1239077	ILMN_251363	-0.84	0.0000
Pdzk1ip1	ILMN_2618935	ILMN_211728	-0.84	0.0017
Add3	ILMN_2588671	ILMN_208695	-0.84	0.0002
Dgkz	ILMN_2915059	ILMN_210741	-0.84	0.0085
Mcfd2	ILMN_3111298	ILMN_250390	-0.85	0.0002
2810022L02Rik	ILMN_2673917	ILMN_214243	-0.85	0.0004
Lgals3	ILMN_1223317	ILMN_222956	-0.85	0.0001
Sdc4	ILMN_2728729	ILMN_220929	-0.86	0.0018
Irx3	ILMN_2776909	ILMN_224225	-0.86	0.0012
Stap1	ILMN_2719803	ILMN_220254	-0.86	0.0000
Sfi1	ILMN_1256699	ILMN_220674	-0.86	0.0022
Enpp1	ILMN_1231851	ILMN_210635	-0.87	0.0009
Hist2h2ac	ILMN_1218380	ILMN_233024	-0.87	0.0006
Col18a1	ILMN_2735184	ILMN_221410	-0.87	0.0073
Ephx1	ILMN_2664224	ILMN_215839	-0.88	0.0008
Smpdl3a	ILMN_1224116	ILMN_209738	-0.88	0.0007
Ttr	ILMN_2443330	ILMN_187347	-0.88	0.0000
Pkp2	ILMN_2927565	ILMN_213446	-0.88	0.0063
Atp13a4	ILMN_2721169	ILMN_236723	-0.88	0.0000
Zfhx3	ILMN_1250928	ILMN_249194	-0.88	0.0036
1700027N10Rik	ILMN_2935237	ILMN_242897	-0.89	0.0001
Il17re	ILMN_2634933	ILMN_213228	-0.89	0.0001
Cdc42se1	ILMN_1259946	ILMN_219464	-0.89	0.0003
Centg2	ILMN_3086136	ILMN_224681	-0.90	0.0007
Slc44a4	ILMN_2970023	ILMN_209371	-0.90	0.0000
Gcnt1	ILMN_1256299	ILMN_214513	-0.91	0.0087
Wwc1	ILMN_2898637	ILMN_229795	-0.91	0.0000
Gnptg	ILMN_2617865	ILMN_208722	-0.91	0.0068
Ass1	ILMN_1247811	ILMN_216093	-0.91	0.0005
Ccnd1	ILMN_2601471	ILMN_210028	-0.91	0.0002
Slc16a6	ILMN_1258950	ILMN_233199	-0.91	0.0005
Tpm1	ILMN_3007072	ILMN_241818	-0.91	0.0005
Ralbp1	ILMN_1231228	ILMN_217172	-0.91	0.0098
Ddx26b	ILMN_1224487	ILMN_215260	-0.92	0.0009
Ccnd1	ILMN_1221503	ILMN_210028	-0.92	0.0019
Prlr	ILMN_2868699	ILMN_210332	-0.92	0.0000
Pglyrp1	ILMN_2592486	ILMN_209096	-0.93	0.0000
Itpr3	ILMN_1228385	ILMN_220408	-0.93	0.0000
Ctage5	ILMN_2645138	ILMN_199381	-0.93	0.0051
Osgin1	ILMN_2645780	ILMN_214233	-0.93	0.0000
5830467P10Rik	ILMN_1234099	ILMN_213543	-0.94	0.0000
Mark2	ILMN_2689887	ILMN_250718	-0.94	0.0004
Frk	ILMN_2615513	ILMN_211404	-0.94	0.0000
Plekhg3	ILMN_1221920	ILMN_222207	-0.94	0.0002
Slc2a1	ILMN_1258159	ILMN_215238	-0.94	0.0007
Apof	ILMN_2644185	ILMN_214094	-0.94	0.0000
Coch	ILMN_2919201	ILMN_222845	-0.95	0.0000
Tmigd1	ILMN_1220817	ILMN_216690	-0.96	0.0000

Spon2	ILMN_1229547	ILMN_223108	-0.96	0.0023
Abca3	ILMN_3150233	ILMN_241076	-0.96	0.0005
Dgka	ILMN_2658961	ILMN_215394	-0.96	0.0031
Trim2	ILMN_2511355	ILMN_187599	-0.97	0.0024
LOC100045542	ILMN_1230893	ILMN_317147	-0.98	0.0007
Ppfibp2	ILMN_2964841	ILMN_223154	-0.98	0.0001
6430527G18Rik	ILMN_3109491	ILMN_230817	-0.98	0.0013
Ptpre	ILMN_1254630	ILMN_215063	-0.98	0.0003
Nup210	ILMN_1257579	ILMN_211743	-0.99	0.0082
Vps37b	ILMN_2681670	ILMN_217304	-0.99	0.0074
Scamp2	ILMN_1226985	ILMN_219369	-1.00	0.0000
Cd24a	ILMN_1237868	ILMN_220466	-1.00	0.0001
Mfsd10	ILMN_1252243	ILMN_214166	-1.00	0.0001
Adamts2	ILMN_1226259	ILMN_210818	-1.00	0.0021
Hist2h3c1	ILMN_3136540	ILMN_227824	-1.01	0.0006
D14Ertd449e	ILMN_2693858	ILMN_243465	-1.01	0.0001
Dnase1	ILMN_2734251	ILMN_214587	-1.02	0.0000
Tbc1d8	ILMN_1255779	ILMN_259676	-1.02	0.0080
St3gal1	ILMN_1224619	ILMN_244745	-1.02	0.0000
2310008M10Rik	ILMN_2904961	ILMN_213855	-1.02	0.0001
Ripk4	ILMN_2840856	ILMN_231082	-1.02	0.0006
Ppap2c	ILMN_3003631	ILMN_240291	-1.03	0.0001
Pkp2	ILMN_2665948	ILMN_213446	-1.03	0.0086
Cldn1	ILMN_2870295	ILMN_219513	-1.03	0.0000
Trim8	ILMN_2782503	ILMN_185225	-1.03	0.0003
Slc46a3	ILMN_2627733	ILMN_212552	-1.03	0.0000
Hist1h2ao	ILMN_2836654	ILMN_196731	-1.03	0.0000
Stard10	ILMN_2759762	ILMN_212367	-1.03	0.0017
Mif	ILMN_2867835	ILMN_207449	-1.04	0.0000
Slc5a1	ILMN_2715270	ILMN_260913	-1.05	0.0000
Pi4k2b	ILMN_2627217	ILMN_207467	-1.05	0.0009
4930504E06Rik	ILMN_1237664	ILMN_212829	-1.06	0.0005
Cx3cl1	ILMN_2627041	ILMN_212491	-1.06	0.0001
Alcam	ILMN_2606804	ILMN_249460	-1.06	0.0004
Abhd14b	ILMN_3007862	ILMN_246267	-1.06	0.0000
Sel1l	ILMN_1244431	ILMN_258443	-1.06	0.0001
Clec2d	ILMN_2603647	ILMN_210243	-1.07	0.0000
Plcg2	ILMN_2601833	ILMN_210061	-1.07	0.0000
Atpif1	ILMN_1250068	ILMN_217464	-1.07	0.0001
Sel1l	ILMN_3137920	ILMN_258443	-1.07	0.0000
Copz2	ILMN_2647028	ILMN_214342	-1.08	0.0000
Atp8b1	ILMN_2977129	ILMN_245949	-1.08	0.0000
Cd63	ILMN_3128907	ILMN_214933	-1.08	0.0000
Rapgef3	ILMN_1256335	ILMN_213815	-1.08	0.0001
Il28ra	ILMN_2653328	ILMN_213007	-1.09	0.0001
Arhgef19	ILMN_1212875	ILMN_219605	-1.09	0.0045
Gnmt	ILMN_2837816	ILMN_215974	-1.09	0.0058
Copz2	ILMN_2878274	ILMN_214342	-1.09	0.0000
Celsr1	ILMN_1235423	ILMN_219113	-1.09	0.0000
H3f3b	ILMN_2648292	ILMN_214469	-1.10	0.0000

Hist2h3b	ILMN_2934120	ILMN_253111	-1.10	0.0003
LOC100044475	ILMN_2596917	ILMN_312482	-1.11	0.0002
Pycr2	ILMN_2998548	ILMN_215594	-1.11	0.0000
Faah	ILMN_2766930	ILMN_215313	-1.11	0.0000
Tcf19	ILMN_2624827	ILMN_212294	-1.11	0.0000
Serpinb6a	ILMN_1250279	ILMN_239826	-1.11	0.0000
Tpd52l1	ILMN_2434472	ILMN_185695	-1.12	0.0000
Lad1	ILMN_1227260	ILMN_217597	-1.12	0.0000
Galnt3	ILMN_1254361	ILMN_246654	-1.12	0.0000
Tspan8	ILMN_1220261	ILMN_194091	-1.13	0.0001
Cmtm7	ILMN_2648409	ILMN_214480	-1.13	0.0007
Cd63	ILMN_2653617	ILMN_214933	-1.13	0.0003
Tmem16a	ILMN_2645341	ILMN_214193	-1.13	0.0017
Scamp1	ILMN_2706853	ILMN_253931	-1.14	0.0000
Gpx2	ILMN_2674483	ILMN_216720	-1.14	0.0001
D4Bwg0951e	ILMN_2646296	ILMN_214279	-1.15	0.0024
Tmem183a	ILMN_2667441	ILMN_249013	-1.15	0.0080
Igsf3	ILMN_1229553	ILMN_229190	-1.15	0.0002
Pak4	ILMN_2640570	ILMN_213768	-1.16	0.0001
Tmem125	ILMN_2703138	ILMN_219015	-1.16	0.0000
Bik	ILMN_2717011	ILMN_220051	-1.16	0.0009
Folr1	ILMN_3000679	ILMN_219347	-1.16	0.0000
Elovl1	ILMN_3143506	ILMN_252169	-1.16	0.0000
Csrp1	ILMN_1260378	ILMN_211706	-1.16	0.0000
Tcn2	ILMN_1221787	ILMN_209382	-1.18	0.0002
Pip4k2c	ILMN_2696191	ILMN_218465	-1.18	0.0000
Lmo2	ILMN_2767605	ILMN_223680	-1.19	0.0000
9130005N14Rik	ILMN_1217235	ILMN_211732	-1.19	0.0000
0610040J01Rik	ILMN_2915716	ILMN_209810	-1.19	0.0000
Pam	ILMN_2626294	ILMN_212425	-1.19	0.0000
LOC100047173	ILMN_2651054	ILMN_312499	-1.20	0.0038
Dsg2	ILMN_1242566	ILMN_245162	-1.20	0.0000
Smap2	ILMN_3120652	ILMN_213072	-1.20	0.0000
Ngfrap1	ILMN_2943599	ILMN_211730	-1.22	0.0001
Ildr1	ILMN_2734693	ILMN_219794	-1.22	0.0000
Creld2	ILMN_2983948	ILMN_222963	-1.22	0.0018
Serpinb1a	ILMN_1231573	ILMN_218572	-1.23	0.0000
Asns	ILMN_3006123	ILMN_213406	-1.23	0.0000
Dpp4	ILMN_2615096	ILMN_211363	-1.23	0.0036
Pdlim1	ILMN_1234072	ILMN_253907	-1.23	0.0036
Rbm47	ILMN_2720836	ILMN_218273	-1.23	0.0000
Tpd52l1	ILMN_1249224	ILMN_185695	-1.24	0.0000
Pafah1b3	ILMN_2640971	ILMN_213804	-1.24	0.0009
Cmtm8	ILMN_1249046	ILMN_211784	-1.24	0.0000
Usp54	ILMN_2418324	ILMN_184400	-1.25	0.0002
LOC100046232	ILMN_2595732	ILMN_310357	-1.25	0.0000
Tuba6	ILMN_2476139	ILMN_191105	-1.25	0.0000
Ankrd56	ILMN_2946760	ILMN_221648	-1.26	0.0000
Tceal8	ILMN_2861493	ILMN_223469	-1.26	0.0008
Arg1	ILMN_2952275	ILMN_255007	-1.26	0.0000

Pqlc1	ILMN_2592266	ILMN_209073	-1.27	0.0000
Hn1	ILMN_2758452	ILMN_223034	-1.27	0.0000
Serpinb6b	ILMN_2611755	ILMN_211031	-1.27	0.0002
Ctsh	ILMN_2872058	ILMN_241653	-1.28	0.0000
Ccdc68	ILMN_2929572	ILMN_223560	-1.29	0.0000
Slc7a4	ILMN_2639360	ILMN_229227	-1.29	0.0000
Cmtm7	ILMN_1233809	ILMN_214480	-1.31	0.0000
Crip2	ILMN_2710449	ILMN_219558	-1.31	0.0000
C230093N12Rik	ILMN_1225348	ILMN_217458	-1.31	0.0000
Sh3yl1	ILMN_1237964	ILMN_220343	-1.31	0.0001
Hn1	ILMN_2914744	ILMN_223034	-1.31	0.0000
Socs2	ILMN_2628178	ILMN_212590	-1.32	0.0000
Asns	ILMN_2643513	ILMN_213406	-1.32	0.0000
Lrrc1	ILMN_2868459	ILMN_219669	-1.32	0.0000
Myh11	ILMN_2622217	ILMN_212053	-1.33	0.0000
Xbp1	ILMN_2431237	ILMN_185934	-1.33	0.0000
Stx7	ILMN_2645255	ILMN_214183	-1.34	0.0000
Liph	ILMN_2760434	ILMN_217708	-1.34	0.0000
Ddah1	ILMN_1256676	ILMN_195643	-1.34	0.0000
Papss1	ILMN_2594477	ILMN_209301	-1.34	0.0008
Acot1	ILMN_3139875	ILMN_223756	-1.35	0.0000
Hmgn3	ILMN_2644719	ILMN_235659	-1.35	0.0000
Nrip3	ILMN_2875404	ILMN_217092	-1.37	0.0000
Ceacam1	ILMN_3126609	ILMN_250809	-1.38	0.0000
Rnase1	ILMN_1220763	ILMN_223373	-1.39	0.0000
Gmds	ILMN_2766596	ILMN_223617	-1.40	0.0004
Pabpc1	ILMN_1259482	ILMN_201529	-1.40	0.0000
Pdlim4	ILMN_2606162	ILMN_210491	-1.41	0.0000
Pllp	ILMN_1249529	ILMN_249550	-1.41	0.0000
Tmem51	ILMN_2589741	ILMN_208808	-1.42	0.0000
Rab4a	ILMN_1212902	ILMN_223403	-1.43	0.0000
Def6	ILMN_2595842	ILMN_209446	-1.43	0.0000
Nudt22	ILMN_1227723	ILMN_218008	-1.43	0.0000
Spcs3	ILMN_2787085	ILMN_240413	-1.44	0.0025
Tcfap2b	ILMN_3151298	ILMN_238889	-1.45	0.0000
Ddr1	ILMN_2713898	ILMN_219438	-1.45	0.0000
Meis2	ILMN_2850391	ILMN_254922	-1.45	0.0000
Elf3	ILMN_2850233	ILMN_212003	-1.45	0.0000
Rasgrp1	ILMN_1246609	ILMN_208941	-1.46	0.0000
Tgfb2	ILMN_1225196	ILMN_247901	-1.46	0.0000
Aass	ILMN_2644092	ILMN_214085	-1.46	0.0000
Anpep	ILMN_2589651	ILMN_208800	-1.46	0.0001
Papss1	ILMN_2859978	ILMN_209301	-1.46	0.0000
Gltp	ILMN_2971816	ILMN_208899	-1.47	0.0000
Cmtm8	ILMN_2801427	ILMN_211784	-1.48	0.0001
Rnf208	ILMN_2636349	ILMN_213366	-1.48	0.0000
Hmgn3	ILMN_1247704	ILMN_214141	-1.49	0.0000
Dstn	ILMN_2801683	ILMN_221304	-1.49	0.0000
Rab3d	ILMN_2588737	ILMN_208703	-1.51	0.0000
Slc9a3r1	ILMN_1240256	ILMN_212760	-1.51	0.0000

C130090K23Rik	ILMN_3163255	ILMN_209707	-1.51	0.0000
Pml	ILMN_2732576	ILMN_249462	-1.51	0.0000
AU040829	ILMN_2702434	ILMN_218962	-1.51	0.0000
3110043J09Rik	ILMN_1228093	ILMN_252216	-1.52	0.0000
LOC100047634	ILMN_1215644	ILMN_309955	-1.52	0.0000
Capn5	ILMN_2627566	ILMN_212539	-1.53	0.0000
Elmo3	ILMN_1219244	ILMN_218257	-1.53	0.0008
Soat1	ILMN_1248510	ILMN_259411	-1.53	0.0001
Galnt10	ILMN_2750011	ILMN_212503	-1.53	0.0008
Prkcd	ILMN_1245750	ILMN_214910	-1.54	0.0000
Lxn	ILMN_2694275	ILMN_218315	-1.54	0.0001
Sgpp1	ILMN_2877165	ILMN_214520	-1.55	0.0000
Es22	ILMN_2627528	ILMN_212535	-1.56	0.0000
Folr1	ILMN_2707541	ILMN_219347	-1.56	0.0000
Scnn1a	ILMN_2729607	ILMN_220993	-1.57	0.0000
Tcfap2b	ILMN_2760450	ILMN_217594	-1.59	0.0000
Sec11c	ILMN_1221592	ILMN_216361	-1.59	0.0000
Cd82	ILMN_2747196	ILMN_222273	-1.59	0.0000
Mup1	ILMN_2875730	ILMN_240932	-1.59	0.0000
Fnbp1l	ILMN_2746132	ILMN_222193	-1.59	0.0000
Kcnj16	ILMN_2613284	ILMN_211173	-1.60	0.0000
Llgl2	ILMN_1224093	ILMN_223065	-1.60	0.0000
6430548M08Rik	ILMN_2599214	ILMN_188148	-1.60	0.0000
LOC100046781	ILMN_2727598	ILMN_313837	-1.60	0.0099
Ccdc120	ILMN_2672543	ILMN_216562	-1.60	0.0000
App	ILMN_2597532	ILMN_209617	-1.61	0.0000
Cftr	ILMN_1259577	ILMN_215935	-1.61	0.0000
Gyltl1b	ILMN_2988849	ILMN_216242	-1.62	0.0000
Eps8l2	ILMN_2821263	ILMN_212969	-1.62	0.0000
Atad4	ILMN_1246558	ILMN_258414	-1.63	0.0000
Lamb3	ILMN_2605512	ILMN_210428	-1.63	0.0000
C130026I21Rik	ILMN_3162925	ILMN_317869	-1.63	0.0000
Cish	ILMN_2718330	ILMN_220158	-1.64	0.0000
LOC100046781	ILMN_1249142	ILMN_313837	-1.64	0.0002
1110003E01Rik	ILMN_1225158	ILMN_215508	-1.65	0.0000
Appl2	ILMN_1219978	ILMN_220140	-1.67	0.0000
Ica1	ILMN_1245768	ILMN_217379	-1.69	0.0000
Wtip	ILMN_2424268	ILMN_225883	-1.69	0.0000
Ngfr	ILMN_2851288	ILMN_246045	-1.69	0.0000
Alox12	ILMN_2613908	ILMN_211240	-1.70	0.0000
Man2b1	ILMN_2707967	ILMN_246706	-1.70	0.0000
Edem2	ILMN_2684279	ILMN_226722	-1.71	0.0000
St3gal4	ILMN_2935870	ILMN_213622	-1.72	0.0000
Nedd9	ILMN_1223678	ILMN_211969	-1.72	0.0000
Adcy8	ILMN_2607127	ILMN_210586	-1.72	0.0000
Prss8	ILMN_2846148	ILMN_221829	-1.73	0.0000
Fpgs	ILMN_2870443	ILMN_221380	-1.73	0.0000
Klf5	ILMN_1253387	ILMN_236003	-1.73	0.0000
Cobl	ILMN_2951682	ILMN_255747	-1.73	0.0000
Krt17	ILMN_2661214	ILMN_215576	-1.74	0.0000

Rab25	ILMN_2781721	ILMN_241897	-1.75	0.0000
Qtrt1	ILMN_1217927	ILMN_219623	-1.75	0.0002
LOC100044177	ILMN_1232495	ILMN_330013	-1.76	0.0000
Asgr1	ILMN_2608703	ILMN_210744	-1.77	0.0000
Serpinb11	ILMN_2834563	ILMN_215688	-1.77	0.0000
Cmas	ILMN_2683128	ILMN_217430	-1.77	0.0000
Acta2	ILMN_2923445	ILMN_261122	-1.77	0.0000
Kdelr3	ILMN_2675697	ILMN_240560	-1.78	0.0000
Krtcap3	ILMN_2859518	ILMN_220056	-1.79	0.0000
Clic6	ILMN_2667635	ILMN_216126	-1.79	0.0000
Aldh18a1	ILMN_3123473	ILMN_253613	-1.80	0.0000
Map3k1	ILMN_3006611	ILMN_211294	-1.80	0.0000
Hexb	ILMN_2829330	ILMN_221504	-1.80	0.0000
2310057J18Rik	ILMN_1220128	ILMN_210967	-1.83	0.0000
Tmem184a	ILMN_2894396	ILMN_214733	-1.83	0.0000
Tmed3	ILMN_2850937	ILMN_214664	-1.84	0.0000
D3Ucla1	ILMN_2805945	ILMN_220728	-1.85	0.0000
Prss8	ILMN_2740965	ILMN_221829	-1.85	0.0000
Acpl2	ILMN_1254634	ILMN_256033	-1.85	0.0000
Zfp750	ILMN_2654571	ILMN_215018	-1.86	0.0000
Kcnk5	ILMN_2982781	ILMN_218669	-1.88	0.0000
Car12	ILMN_2891583	ILMN_233734	-1.90	0.0000
Ero1lb	ILMN_2692960	ILMN_218209	-1.90	0.0000
Faah	ILMN_2657980	ILMN_215313	-1.90	0.0000
D530004J12Rik	ILMN_2703364	ILMN_219031	-1.91	0.0000
Foxa1	ILMN_1237195	ILMN_221873	-1.91	0.0000
Marveld3	ILMN_2632585	ILMN_213009	-1.91	0.0000
Nedd9	ILMN_2654186	ILMN_211969	-1.91	0.0000
Cyp2s1	ILMN_2758264	ILMN_223026	-1.92	0.0000
Scnn1g	ILMN_2759914	ILMN_223147	-1.92	0.0000
Ap1m2	ILMN_2925872	ILMN_211827	-1.93	0.0000
Liph	ILMN_1217063	ILMN_217708	-1.93	0.0000
Irx2	ILMN_3115213	ILMN_256714	-1.93	0.0003
Meis1	ILMN_1218266	ILMN_220284	-1.95	0.0000
Wwc1	ILMN_2525423	ILMN_229795	-1.96	0.0000
Rgs1	ILMN_2897891	ILMN_226813	-1.96	0.0000
LOC100047619	ILMN_2711948	ILMN_313920	-1.97	0.0000
Dsp	ILMN_2654997	ILMN_255543	-1.98	0.0000
LOC626152	ILMN_1243900	ILMN_316341	-2.00	0.0000
Emb	ILMN_1218799	ILMN_208803	-2.02	0.0000
AW555464	ILMN_2685769	ILMN_217638	-2.02	0.0000
Irf6	ILMN_1216279	ILMN_215395	-2.04	0.0000
2410002F23Rik	ILMN_2629648	ILMN_212730	-2.04	0.0000
Dtnb	ILMN_2920027	ILMN_219325	-2.05	0.0000
D330028D13Rik	ILMN_1217670	ILMN_218922	-2.05	0.0000
Ildr1	ILMN_2857684	ILMN_219794	-2.06	0.0000
9130404D14Rik	ILMN_2686975	ILMN_217737	-2.06	0.0000
2410002F23Rik	ILMN_2965641	ILMN_212730	-2.07	0.0000
Pip5k1b	ILMN_2711562	ILMN_219634	-2.07	0.0000
St5	ILMN_1250665	ILMN_235447	-2.09	0.0000

Elf5	ILMN_2938440	ILMN_216298	-2.10	0.0000
Ap1m2	ILMN_2762318	ILMN_211827	-2.10	0.0000
Efcab4a	ILMN_2720663	ILMN_246608	-2.11	0.0000
Acta2	ILMN_2693895	ILMN_261122	-2.12	0.0000
Bsnd	ILMN_2669493	ILMN_216297	-2.13	0.0000
1500015O10Rik	ILMN_1249000	ILMN_222808	-2.13	0.0000
5330417C22Rik	ILMN_3160472	ILMN_231220	-2.13	0.0000
Kndc1	ILMN_2757844	ILMN_256267	-2.13	0.0000
Nans	ILMN_1245757	ILMN_215041	-2.14	0.0000
Gdpd1	ILMN_2683528	ILMN_217468	-2.16	0.0000
Tes	ILMN_3084954	ILMN_216487	-2.17	0.0000
Grhl1	ILMN_1246419	ILMN_214278	-2.18	0.0000
Smpdl3b	ILMN_2602938	ILMN_210171	-2.19	0.0000
Bglap1	ILMN_3101908	ILMN_210884	-2.20	0.0000
Gjb2	ILMN_1227148	ILMN_210347	-2.20	0.0000
Prlr	ILMN_2617005	ILMN_210332	-2.20	0.0000
Mtap2	ILMN_3137552	ILMN_214246	-2.21	0.0000
Pdcd4	ILMN_2898878	ILMN_212354	-2.21	0.0000
St5	ILMN_3154820	ILMN_210050	-2.21	0.0000
Jam4	ILMN_2919999	ILMN_217986	-2.23	0.0000
Tuft1	ILMN_2419185	ILMN_184505	-2.23	0.0000
D10Bwg1379e	ILMN_2781181	ILMN_239049	-2.23	0.0000
Ptprk	ILMN_2879600	ILMN_227089	-2.24	0.0000
Cadps2	ILMN_2998313	ILMN_208854	-2.24	0.0000
Ascl3	ILMN_2691996	ILMN_218130	-2.27	0.0000
Ly6g6e	ILMN_2991389	ILMN_259052	-2.28	0.0000
Tram1	ILMN_2487934	ILMN_192424	-2.29	0.0000
Atad4	ILMN_2788191	ILMN_258414	-2.29	0.0000
Itgb4	ILMN_3144575	ILMN_249306	-2.32	0.0000
Grhl2	ILMN_1257070	ILMN_240233	-2.32	0.0000
Gyltl1b	ILMN_1235131	ILMN_216242	-2.33	0.0000
P4hb	ILMN_2735996	ILMN_221467	-2.34	0.0000
AI428936	ILMN_2619639	ILMN_211797	-2.35	0.0000
Spnb3	ILMN_2661588	ILMN_240209	-2.36	0.0000
Aldh1l2	ILMN_2898319	ILMN_222938	-2.37	0.0000
Lman1l	ILMN_3127595	ILMN_214284	-2.37	0.0000
Stk39	ILMN_2751494	ILMN_222561	-2.39	0.0000
Foxc1	ILMN_2886260	ILMN_214651	-2.40	0.0000
Tram1	ILMN_2781938	ILMN_192424	-2.41	0.0000
4922503N01Rik	ILMN_2939503	ILMN_210268	-2.42	0.0000
Cd9	ILMN_2725414	ILMN_220691	-2.44	0.0000
Krt7	ILMN_2961152	ILMN_223017	-2.45	0.0000
Cyb561	ILMN_1232601	ILMN_219873	-2.46	0.0000
Rrbp1	ILMN_2612079	ILMN_211061	-2.47	0.0000
Cldn3	ILMN_2634167	ILMN_213147	-2.48	0.0000
Tns4	ILMN_1236029	ILMN_216838	-2.48	0.0000
Tcfap2b	ILMN_1223731	ILMN_238889	-2.49	0.0000
Slit2	ILMN_1253797	ILMN_259247	-2.54	0.0000
Tmem176a	ILMN_2795412	ILMN_216668	-2.54	0.0000
Efcab4a	ILMN_2913222	ILMN_246608	-2.54	0.0000

Pof1b	ILMN_1242912	ILMN_221065	-2.54	0.0000
Atp1b1	ILMN_2767615	ILMN_223681	-2.58	0.0000
Slc12a2	ILMN_2700059	ILMN_211631	-2.59	0.0000
Cldn4	ILMN_1223949	ILMN_209388	-2.60	0.0000
Abpg	ILMN_2696136	ILMN_218461	-2.64	0.0000
Gne	ILMN_2682503	ILMN_251062	-2.67	0.0000
Gstt3	ILMN_2665715	ILMN_215961	-2.67	0.0000
Grb7	ILMN_2773169	ILMN_247424	-2.68	0.0000
Garnl4	ILMN_2940713	ILMN_226588	-2.69	0.0000
LOC100046616	ILMN_1259982	ILMN_324507	-2.70	0.0000
BC017612	ILMN_2631514	ILMN_234140	-2.71	0.0000
Mup4	ILMN_2592166	ILMN_209061	-2.73	0.0000
Foxq1	ILMN_1224637	ILMN_221286	-2.74	0.0000
Wbp5	ILMN_2429025	ILMN_185673	-2.75	0.0000
Atp1a1	ILMN_1218058	ILMN_222215	-2.76	0.0000
Nupr1	ILMN_2742042	ILMN_221920	-2.77	0.0000
Cxcl17	ILMN_1226394	ILMN_211385	-2.78	0.0000
1190003J15Rik	ILMN_2734924	ILMN_238108	-2.78	0.0000
Scnn1b	ILMN_2618383	ILMN_211671	-2.80	0.0000
Rab27a	ILMN_2614966	ILMN_211354	-2.80	0.0000
Herc2	ILMN_2717727	ILMN_220107	-2.82	0.0000
Tpd52	ILMN_3091288	ILMN_246767	-2.82	0.0000
Tmprss2	ILMN_1223880	ILMN_189973	-2.83	0.0000
Tmem176b	ILMN_1259470	ILMN_211657	-2.84	0.0000
Gjb2	ILMN_2999627	ILMN_210347	-2.85	0.0000
Acpp	ILMN_2693019	ILMN_218213	-2.86	0.0000
LOC100048733	ILMN_2759344	ILMN_318857	-2.89	0.0000
Krt14	ILMN_2722616	ILMN_220479	-2.89	0.0000
Oit1	ILMN_2732163	ILMN_221188	-2.90	0.0000
Slc5a8	ILMN_2739384	ILMN_209122	-2.93	0.0000
BC024561	ILMN_2783414	ILMN_211385	-2.93	0.0000
Sidt1	ILMN_1248211	ILMN_256339	-2.93	0.0000
Cdh1	ILMN_2628629	ILMN_212630	-2.97	0.0000
Fa2h	ILMN_2746783	ILMN_222241	-3.01	0.0000
Iqgap2	ILMN_2908846	ILMN_232331	-3.02	0.0000
Tesc	ILMN_2748222	ILMN_222340	-3.13	0.0000
Bglap1	ILMN_2610166	ILMN_247195	-3.15	0.0000
Muc13	ILMN_2717678	ILMN_217318	-3.15	0.0000
1600029D21Rik	ILMN_1259777	ILMN_222410	-3.18	0.0000
Kcnn4	ILMN_2765032	ILMN_223516	-3.19	0.0000
Kcnk1	ILMN_3009501	ILMN_237297	-3.25	0.0000
Elf5	ILMN_2736380	ILMN_216298	-3.38	0.0000
Slc12a2	ILMN_3146420	ILMN_211631	-3.42	0.0000
Csprs	ILMN_2661289	ILMN_215584	-3.42	0.0000
Unc13b	ILMN_1220360	ILMN_248534	-3.44	0.0000
Klk1b1	ILMN_1256119	ILMN_196746	-3.44	0.0000
Mansc1	ILMN_2848273	ILMN_212652	-3.46	0.0000
Bglap2	ILMN_2944508	ILMN_226483	-3.50	0.0000
Cited4	ILMN_2998934	ILMN_255417	-3.57	0.0000
Lpo	ILMN_2684053	ILMN_217505	-3.57	0.0000

Pigr	ILMN_1225605	ILMN_220227	-3.62	0.0000
Krt8	ILMN_1221157	ILMN_212791	-3.64	0.0000
Smgc	ILMN_2775962	ILMN_244638	-3.67	0.0000
OTTMUSG00000007485	ILMN_2881950	ILMN_241785	-3.70	0.0000
Phlda1	ILMN_2754985	ILMN_222798	-3.71	0.0000
Cyp2f2	ILMN_2702903	ILMN_218995	-3.72	0.0000
Krt18	ILMN_2711267	ILMN_219615	-3.74	0.0000
Prom2	ILMN_1246392	ILMN_214759	-3.76	0.0000
Ngfb	ILMN_2660233	ILMN_215493	-3.77	0.0000
Slc12a8	ILMN_2721789	ILMN_312656	-3.77	0.0000
Bhlhb8	ILMN_2664777	ILMN_214740	-3.77	0.0000
Car6	ILMN_1257323	ILMN_258206	-3.79	0.0000
Fkbp11	ILMN_1224635	ILMN_210599	-3.80	0.0000
Clcnkb	ILMN_2757283	ILMN_234837	-3.91	0.0000
Fxyd3	ILMN_2595593	ILMN_209417	-3.91	0.0000
Npal2	ILMN_2723369	ILMN_235586	-3.97	0.0000
Rab15	ILMN_1217009	ILMN_218127	-3.97	0.0000
Ppp1r1b	ILMN_2954824	ILMN_214764	-4.00	0.0000
Fxyd2	ILMN_1251018	ILMN_211483	-4.05	0.0000
Ngfb	ILMN_2937596	ILMN_215493	-4.07	0.0000
Mist1	ILMN_2651388	ILMN_214740	-4.17	0.0000
Lrrc26	ILMN_2682268	ILMN_217355	-4.17	0.0000
Ncald	ILMN_2652414	ILMN_247828	-4.19	0.0000
Cldn10	ILMN_2723576	ILMN_252548	-4.22	0.0000
Atp2a3	ILMN_2688236	ILMN_213534	-4.26	0.0000
Aqp5	ILMN_3115472	ILMN_217154	-4.28	0.0000
Ckmt1	ILMN_2773537	ILMN_235471	-4.28	0.0000
Atp2a3	ILMN_2900462	ILMN_213534	-4.29	0.0000
Rbm35a	ILMN_2947559	ILMN_210806	-4.39	0.0000
Bhlhb8	ILMN_2651389	ILMN_214740	-4.41	0.0000
Cldn10	ILMN_1214954	ILMN_209356	-4.43	0.0000
Abpg	ILMN_2599449	ILMN_209813	-4.45	0.0000
Bglap-rs1	ILMN_1233122	ILMN_236469	-4.49	0.0000
Fxyd2	ILMN_2616328	ILMN_211483	-4.53	0.0000
Wfdc2	ILMN_1236758	ILMN_216447	-4.74	0.0000
Krt23	ILMN_2671165	ILMN_216445	-4.85	0.0000
Kal1	ILMN_1239717	ILMN_223014	-4.92	0.0000
Egf	ILMN_2684104	ILMN_217509	-4.94	0.0000
Klk1b21	ILMN_2732087	ILMN_196711	-5.20	0.0000
Klk1b21	ILMN_2651099	ILMN_196711	-5.23	0.0000
Cldn10	ILMN_2515816	ILMN_252548	-5.41	0.0000
Klk1b11	ILMN_2632912	ILMN_196710	-5.42	0.0000
Klk1b16	ILMN_1259613	ILMN_196767	-5.71	0.0000
Smgc	ILMN_2602496	ILMN_210125	-5.72	0.0000
Klk1b24	ILMN_2622463	ILMN_196770	-5.76	0.0000
Klk1b9	ILMN_2723594	ILMN_196709	-5.80	0.0000
2310057J18Rik	ILMN_2940678	ILMN_210967	-5.98	0.0000
Klk1b5	ILMN_1224893	ILMN_196768	-6.04	0.0000
Smgc	ILMN_2620853	ILMN_210125	-6.04	0.0000
Psp	ILMN_1245726	ILMN_216963	-6.18	0.0000

Klk1b27	ILMN_3009447	ILMN_196774	-6.20	0.0000
Klk1b9	ILMN_2784773	ILMN_196709	-6.26	0.0000
Klk1b8	ILMN_1216962	ILMN_212623	-6.31	0.0000
Abpa	ILMN_1249191	ILMN_216701	-6.35	0.0000
Klk1b4	ILMN_2697256	ILMN_199361	-6.51	0.0000
Wfdc12	ILMN_2948296	ILMN_196009	-6.62	0.0000
Klk1b22	ILMN_2946653	ILMN_239601	-6.64	0.0000
Klk1b11	ILMN_2979432	ILMN_196710	-6.64	0.0000
Egfbp2	ILMN_2791241	ILMN_196757	-6.67	0.0000
Mup5	ILMN_3158509	ILMN_214274	-6.67	0.0000
Klk1b26	ILMN_1217308	ILMN_196712	-6.84	0.0000
Klk1b5	ILMN_2731191	ILMN_196768	-6.86	0.0000
Abpb	ILMN_2915893	ILMN_250100	-6.87	0.0000
Klk1b4	ILMN_1238736	ILMN_199361	-6.94	0.0000
Pip	ILMN_2621766	ILMN_212014	-6.98	0.0000
LOC100044256	ILMN_2703052	ILMN_313374	-6.99	0.0000
Muc10	ILMN_1247393	ILMN_212146	-7.01	0.0000
Abpb	ILMN_2771109	ILMN_315214	-7.01	0.0000
Klk1	ILMN_2760199	ILMN_196747	-7.04	0.0000
Mup5	ILMN_3079257	ILMN_214274	-7.07	0.0000
Spt1	ILMN_2845370	ILMN_217293	-7.15	0.0000
Muc10	ILMN_2623234	ILMN_212146	-7.15	0.0000
Klk1b27	ILMN_1252131	ILMN_196774	-7.23	0.0000
