# Supplementary material for: Deconvolution of expression microarray data reveals 131I-induced responses otherwise undetected in thyroid tissue
Source: PLoS One. 2018 Jul 12;13(7):e0197911. doi: 10.1371/journal.pone.0197911 (PMC6042689; doi:10.1371/journal.pone.0197911)
Supplement: S3 Table — (PDF) [file pone.0197911.s005.pdf]

Supplemental Table 3. List of significantly regulated transcripts in deconvolved data for follicular cells

The log2-ratio threshold was set to 0.58 with a p-value threshold of 0.01.
Multiple test correction for FDR was performed using the Benjamini Hochberg method in NEXUS 3.0 (BioDiscovery; El Segundo, CA, USA).
The pool size for intensity based pooling was set to 200.

Gene Symbol	Probe	Transcript	Log_2 ratio	adjusted p-value
Enpp7	ILMN_3075190	ILMN_248310	11.92	0.0000
Tmem23	ILMN_2480682	ILMN_191618	9.75	0.0000
Lbh	ILMN_1233545	ILMN_215742	9.71	0.0000
Itgbl1	ILMN_2636424	ILMN_236699	9.67	0.0000
4933412E14Rik	ILMN_2680238	ILMN_217200	9.29	0.0000
Mat2a	ILMN_1258415	ILMN_261868	8.53	0.0000
Acta1	ILMN_2738825	ILMN_221671	8.14	0.0000
Tnni2	ILMN_2481133	ILMN_191670	8.14	0.0000
Lmna	ILMN_2662387	ILMN_209635	7.87	0.0000
Atp2a1	ILMN_2666864	ILMN_216061	7.83	0.0000
Samhd1	ILMN_1221736	ILMN_211012	7.65	0.0000
Psmb8	ILMN_2693940	ILMN_259219	7.14	0.0000
Tcap	ILMN_2933357	ILMN_218508	7.11	0.0000
Prpf39	ILMN_2692799	ILMN_218194	7.08	0.0000
Tnnc2	ILMN_2882658	ILMN_188936	6.84	0.0000
Krtcap2	ILMN_2605694	ILMN_210448	6.70	0.0000
Upf1	ILMN_2604108	ILMN_239726	6.66	0.0000
Mcfd2	ILMN_3111298	ILMN_250390	6.65	0.0000
Npm3-ps1	ILMN_2965417	ILMN_255379	6.61	0.0000
Zgpat	ILMN_3157372	ILMN_261515	6.60	0.0000
Slc30a5	ILMN_1221208	ILMN_211338	6.51	0.0000
Slc40a1	ILMN_2747923	ILMN_222315	6.48	0.0000
Arhgap29	ILMN_2753687	ILMN_237863	6.40	0.0000
Ada	ILMN_1228696	ILMN_217667	6.20	0.0000
Myom1	ILMN_2815138	ILMN_241505	6.19	0.0000
Tpm4	ILMN_2515363	ILMN_237612	6.08	0.0000
1700047I17Rik1	ILMN_3034034	ILMN_221070	6.07	0.0000
Ttn	ILMN_2416670	ILMN_184200	6.04	0.0000
Vps28	ILMN_1226704	ILMN_194493	6.00	0.0000
Zfp61	ILMN_2475363	ILMN_188854	5.85	0.0000
Ttn	ILMN_3128792	ILMN_255016	5.79	0.0000
Atp6ap2	ILMN_2628657	ILMN_210527	5.67	0.0000
Hnrph3	ILMN_2958912	ILMN_255042	5.67	0.0000
A2bp1	ILMN_1238309	ILMN_216899	5.65	0.0000
Supt4h1	ILMN_1247505	ILMN_231446	5.45	0.0000
Mtus1	ILMN_3064383	ILMN_244478	5.39	0.0000
2010106G01Rik	ILMN_1252689	ILMN_220370	5.37	0.0000
Ubtd2	ILMN_2669259	ILMN_216275	5.36	0.0000
Tbcd	ILMN_2891506	ILMN_255544	5.36	0.0000
2410022L05Rik	ILMN_1234516	ILMN_214324	5.36	0.0000
Nol9	ILMN_3160348	ILMN_223288	5.34	0.0000
Asb2	ILMN_2765759	ILMN_223558	5.33	0.0000

Olr1	ILMN_2745565	ILMN_216724	5.31	0.0000
Ankrd54	ILMN_2600078	ILMN_209878	5.30	0.0000
Grap	ILMN_2617656	ILMN_211613	5.27	0.0000
Ube2q1	ILMN_1259048	ILMN_191561	5.27	0.0000
AW146242	ILMN_2735429	ILMN_221426	5.26	0.0000
2610209M04Rik	ILMN_2860750	ILMN_223751	5.26	0.0000
Cox6a2	ILMN_2629581	ILMN_212725	5.22	0.0000
Dcp1a	ILMN_2783429	ILMN_218539	5.20	0.0000
Uchl1	ILMN_1225261	ILMN_190011	5.20	0.0000
E130309F12Rik	ILMN_1218474	ILMN_210734	5.17	0.0036
Fbxo32	ILMN_2873444	ILMN_219973	5.13	0.0000
Igf2bp3	ILMN_1218913	ILMN_218056	5.12	0.0000
Etsrp71	ILMN_2678641	ILMN_217057	5.09	0.0000
Fbxo33	ILMN_3162369	ILMN_234957	5.07	0.0000
Fos	ILMN_2750515	ILMN_222500	5.05	0.0001
Mrpl51	ILMN_2831273	ILMN_199367	5.01	0.0000
4933417E01Rik	ILMN_1226326	ILMN_318969	5.01	0.0000
Wdr5b	ILMN_2425488	ILMN_256044	5.01	0.0000
LOC100045542	ILMN_1230893	ILMN_317147	5.00	0.0000
Trp53inp1	ILMN_2506012	ILMN_194391	4.99	0.0000
Crisp2	ILMN_2715114	ILMN_219913	4.96	0.0000
Reps1	ILMN_1250054	ILMN_210950	4.95	0.0000
Xpo4	ILMN_1231751	ILMN_185320	4.94	0.0000
AA407659	ILMN_2876010	ILMN_208916	4.93	0.0000
Sema3f	ILMN_1248740	ILMN_213287	4.93	0.0000
LOC669001	ILMN_1256102	ILMN_241214	4.92	0.0000
Rad50	ILMN_2867427	ILMN_214521	4.92	0.0000
Mrps18c	ILMN_2747345	ILMN_244766	4.91	0.0014
Prim2	ILMN_2982965	ILMN_222217	4.90	0.0000
Hnrpk	ILMN_1245987	ILMN_213571	4.84	0.0001
Trim33	ILMN_1236637	ILMN_188197	4.81	0.0000
Plekhg2	ILMN_2987027	ILMN_210871	4.79	0.0000
Nr1h2	ILMN_3014084	ILMN_225790	4.79	0.0000
Prss8	ILMN_2740965	ILMN_221829	4.72	0.0000
Cytl1	ILMN_2877029	ILMN_255006	4.71	0.0000
AA407270	ILMN_2913989	ILMN_261110	4.69	0.0000
Sec14l1	ILMN_2680308	ILMN_238122	4.69	0.0000
Gdap1l1	ILMN_2989312	ILMN_210626	4.68	0.0000
Cript	ILMN_1223929	ILMN_220029	4.66	0.0000
Senp7	ILMN_1260543	ILMN_259312	4.65	0.0000
Ahnak2	ILMN_3161105	ILMN_254174	4.65	0.0000
Mpp5	ILMN_2590811	ILMN_208920	4.65	0.0029
Dyrk3	ILMN_2699522	ILMN_258582	4.64	0.0000
Syngr1	ILMN_2657751	ILMN_214854	4.63	0.0000
Kars	ILMN_2606848	ILMN_210561	4.63	0.0000
Cherp	ILMN_2746924	ILMN_222253	4.62	0.0008
Cpsf3	ILMN_2870410	ILMN_218395	4.62	0.0000
Zfp459	ILMN_1253331	ILMN_225147	4.60	0.0000
Zscan2	ILMN_2481127	ILMN_191669	4.59	0.0000
Mrc2	ILMN_2639981	ILMN_251607	4.59	0.0000

Slc39a5	ILMN_2705424	ILMN_219188	4.57	0.0000
Gimap4	ILMN_1225854	ILMN_222975	4.57	0.0000
Kynu	ILMN_1214750	ILMN_219585	4.57	0.0000
Olfr6	ILMN_2708938	ILMN_219441	4.55	0.0000
Aif1	ILMN_2804487	ILMN_215740	4.54	0.0000
Sepm	ILMN_1226838	ILMN_219316	4.51	0.0000
Napa	ILMN_2865822	ILMN_212166	4.51	0.0000
Tas2r114	ILMN_1248846	ILMN_223207	4.49	0.0000
Nans	ILMN_1245757	ILMN_215041	4.49	0.0018
Epha3	ILMN_2629805	ILMN_212746	4.48	0.0000
Sesn3	ILMN_2622997	ILMN_228252	4.47	0.0000
Pik3ip1	ILMN_2769772	ILMN_210015	4.47	0.0000
LOC100045522	ILMN_3163529	ILMN_322016	4.47	0.0000
Mbd1	ILMN_2893081	ILMN_210686	4.45	0.0000
Psmd11	ILMN_1230682	ILMN_213316	4.43	0.0010
Foxo1	ILMN_2656498	ILMN_242706	4.43	0.0000
Brd4	ILMN_2627582	ILMN_210066	4.42	0.0005
Fbxo32	ILMN_2752994	ILMN_219973	4.42	0.0000
Tgfbr1	ILMN_2708965	ILMN_210148	4.41	0.0068
Lonrf2	ILMN_2848288	ILMN_247187	4.41	0.0000
Snta1	ILMN_2734142	ILMN_221335	4.40	0.0000
Commd2	ILMN_2642615	ILMN_210241	4.39	0.0000
BC024997	ILMN_1247389	ILMN_222811	4.39	0.0057
4930427A07Rik	ILMN_1217337	ILMN_221758	4.38	0.0000
Gpkow	ILMN_2728189	ILMN_214633	4.38	0.0071
Ccdc21	ILMN_2759983	ILMN_223152	4.37	0.0000
Clec7a	ILMN_2653132	ILMN_260465	4.37	0.0000
Trim30	ILMN_1229630	ILMN_257424	4.37	0.0011
D3Wsu161e	ILMN_2636456	ILMN_213376	4.35	0.0000
Tmem49	ILMN_2616768	ILMN_209017	4.35	0.0001
Hsdl1	ILMN_2691227	ILMN_218076	4.35	0.0000
Slc24a6	ILMN_2643377	ILMN_214023	4.34	0.0000
Ddx19a	ILMN_1233412	ILMN_218762	4.34	0.0000
Ankrd2	ILMN_2806065	ILMN_214035	4.33	0.0000
Cort	ILMN_1226607	ILMN_209317	4.33	0.0000
Icmt	ILMN_2791410	ILMN_255616	4.32	0.0000
AA691260	ILMN_2606136	ILMN_193283	4.32	0.0000
Mfsd10	ILMN_2836586	ILMN_214166	4.31	0.0000
Mreg	ILMN_2976159	ILMN_253687	4.31	0.0015
Ly6d	ILMN_2803921	ILMN_223061	4.31	0.0000
Nrap	ILMN_1235070	ILMN_217102	4.30	0.0000
1110020G09Rik	ILMN_1237830	ILMN_313573	4.28	0.0090
Klhdc4	ILMN_2602115	ILMN_237570	4.28	0.0069
Gtf2e2	ILMN_2817371	ILMN_222459	4.27	0.0000
Rab7l1	ILMN_2681186	ILMN_217263	4.27	0.0000
Peli3	ILMN_2619678	ILMN_211803	4.27	0.0000
Asb3	ILMN_2663555	ILMN_246247	4.26	0.0000
Ptp4a3	ILMN_2655260	ILMN_213418	4.25	0.0003
Ttc7	ILMN_2946689	ILMN_257003	4.23	0.0001
Btg2	ILMN_1256772	ILMN_219869	4.22	0.0000

Rnf135	ILMN_2655034	ILMN_215057	4.21	0.0000
Dhx30	ILMN_1233008	ILMN_212148	4.20	0.0000
Actrt1	ILMN_2618100	ILMN_211644	4.20	0.0047
4933439C20Rik	ILMN_2944601	ILMN_241433	4.19	0.0000
Rbm43	ILMN_2995426	ILMN_218064	4.19	0.0000
Snap91	ILMN_2597032	ILMN_209567	4.19	0.0000
Kremen1	ILMN_2651035	ILMN_230470	4.18	0.0008
Ercc6	ILMN_2688327	ILMN_260837	4.17	0.0000
Jarid1c	ILMN_2970823	ILMN_247102	4.17	0.0018
Ube2k	ILMN_2655066	ILMN_213294	4.17	0.0002
Myd116	ILMN_2722938	ILMN_220505	4.17	0.0000
Cox7a2l	ILMN_2888713	ILMN_238225	4.15	0.0000
Snapc3	ILMN_1213437	ILMN_249101	4.15	0.0000
H2afz	ILMN_3074985	ILMN_222997	4.15	0.0002
Eif2c2	ILMN_2609056	ILMN_226444	4.14	0.0000
Nr1d2	ILMN_2602387	ILMN_210116	4.13	0.0005
1190002A17Rik	ILMN_2590894	ILMN_258501	4.13	0.0005
Igf2bp3	ILMN_1226175	ILMN_218056	4.13	0.0000
Zfp410	ILMN_2840327	ILMN_231991	4.12	0.0000
Plscr1	ILMN_2911344	ILMN_218871	4.12	0.0000
Cebpa	ILMN_1247147	ILMN_231653	4.12	0.0000
A730011L01Rik	ILMN_1238805	ILMN_208999	4.11	0.0000
Zfp523	ILMN_1236258	ILMN_196146	4.10	0.0004
Nrap	ILMN_3061287	ILMN_247151	4.09	0.0001
Flcn	ILMN_1213483	ILMN_210700	4.09	0.0000
Fes	ILMN_2695793	ILMN_218433	4.09	0.0028
Defcr6	ILMN_1255021	ILMN_196559	4.09	0.0000
Eya3	ILMN_2761966	ILMN_210388	4.08	0.0000
Junb	ILMN_1220034	ILMN_221987	4.08	0.0001
Tmem2	ILMN_2430220	ILMN_246039	4.08	0.0002
Actn3	ILMN_2708303	ILMN_219398	4.08	0.0000
Btbd9	ILMN_2643933	ILMN_214072	4.08	0.0001
Ecm1	ILMN_2947526	ILMN_213475	4.07	0.0000
2310010B21Rik	ILMN_2696360	ILMN_218479	4.07	0.0000
Myot	ILMN_2444217	ILMN_250811	4.07	0.0000
Acss1	ILMN_2628174	ILMN_212588	4.07	0.0000
Chmp4c	ILMN_2601851	ILMN_210063	4.06	0.0001
Mmp2	ILMN_2678218	ILMN_217021	4.06	0.0001
Kif4	ILMN_2747641	ILMN_222148	4.05	0.0021
Ddhd1	ILMN_2446727	ILMN_187741	4.04	0.0004
Sfrs2	ILMN_1235653	ILMN_213364	4.02	0.0000
Athl1	ILMN_2910684	ILMN_240733	4.01	0.0000
Ltf	ILMN_2754364	ILMN_222761	4.01	0.0010
Zfp87	ILMN_2992836	ILMN_191750	4.01	0.0001
Pld3	ILMN_2702101	ILMN_218931	4.00	0.0000
Myh9	ILMN_2672626	ILMN_216568	3.99	0.0001
Sf1	ILMN_2493851	ILMN_188043	3.99	0.0000
Naca	ILMN_2651643	ILMN_214762	3.99	0.0000
Ada	ILMN_2686132	ILMN_217667	3.98	0.0000
Hnt	ILMN_2681195	ILMN_260609	3.98	0.0041

AU040320	ILMN_3024494	ILMN_241990	3.97	0.0000
Thbs3	ILMN_2654651	ILMN_210110	3.97	0.0000
Ctsk	ILMN_2711163	ILMN_219610	3.94	0.0003
EG317677	ILMN_1260226	ILMN_213670	3.91	0.0000
5630401D24Rik	ILMN_2754492	ILMN_221329	3.91	0.0000
Polr3k	ILMN_1215772	ILMN_213704	3.91	0.0009
Ccs	ILMN_1253618	ILMN_215857	3.90	0.0086
EG432870	ILMN_3161312	ILMN_250183	3.90	0.0000
Ceacam18	ILMN_1217029	ILMN_231109	3.89	0.0001
Glra2	ILMN_2729364	ILMN_220976	3.88	0.0000
Top2a	ILMN_2830661	ILMN_187810	3.87	0.0000
Bclaf1	ILMN_3096361	ILMN_242377	3.87	0.0001
Midn	ILMN_2658566	ILMN_209610	3.86	0.0011
Kif1b	ILMN_2654314	ILMN_214427	3.86	0.0004
Eif2ak1	ILMN_2709397	ILMN_193332	3.85	0.0054
1190005P17Rik	ILMN_2596834	ILMN_209045	3.85	0.0000
Brca2	ILMN_2634750	ILMN_213209	3.84	0.0000
Lsm5	ILMN_2971946	ILMN_259737	3.83	0.0000
Chac1	ILMN_2617468	ILMN_211596	3.83	0.0000
Cox7a2	ILMN_2733698	ILMN_221305	3.83	0.0030
Adam6	ILMN_1238157	ILMN_210813	3.83	0.0028
Lce3f	ILMN_2610594	ILMN_240447	3.83	0.0081
Pbx3	ILMN_2650972	ILMN_210749	3.82	0.0000
Pcdhga2	ILMN_2746009	ILMN_222189	3.82	0.0059
6430526O11Rik	ILMN_2728255	ILMN_219257	3.79	0.0063
Arl6ip2	ILMN_3060766	ILMN_215568	3.79	0.0001
Nhlrc3	ILMN_1219639	ILMN_225167	3.79	0.0000
Ankrd12	ILMN_3151492	ILMN_258504	3.77	0.0003
Spnb2	ILMN_1244992	ILMN_209605	3.77	0.0000
Gga3	ILMN_1242273	ILMN_254242	3.77	0.0002
Zfp330	ILMN_2440789	ILMN_187056	3.77	0.0008
Tug1	ILMN_3028324	ILMN_260041	3.77	0.0019
LOC225594	ILMN_2996973	ILMN_261460	3.76	0.0002
Aldh3b1	ILMN_2645793	ILMN_214234	3.76	0.0004
Chmp5	ILMN_2670352	ILMN_216374	3.75	0.0000
Mrpl52	ILMN_2601865	ILMN_210065	3.75	0.0000
Twf2	ILMN_1234388	ILMN_212893	3.74	0.0000
Osr1	ILMN_1259787	ILMN_213374	3.73	0.0000
Tnfrsf26	ILMN_2479124	ILMN_191444	3.73	0.0000
Ctsw	ILMN_2721399	ILMN_220388	3.73	0.0001
Fastkd2	ILMN_2817960	ILMN_216878	3.73	0.0000
Sostdc1	ILMN_2642800	ILMN_213965	3.72	0.0001
Scarb1	ILMN_1223315	ILMN_213357	3.72	0.0098
Mtus1	ILMN_3162801	ILMN_244329	3.72	0.0000
Pnliprp2	ILMN_2692167	ILMN_218140	3.71	0.0000
Tex264	ILMN_1247681	ILMN_223212	3.71	0.0002
Ccl6	ILMN_2694179	ILMN_218308	3.70	0.0000
Krtap16-3	ILMN_2856457	ILMN_208768	3.70	0.0000
EG622976	ILMN_2804283	ILMN_243733	3.70	0.0004
Ctnnbl1	ILMN_1249568	ILMN_212264	3.70	0.0003

Dolpp1	ILMN_2723338	ILMN_220141	3.69	0.0001
Atm	ILMN_2647620	ILMN_214401	3.69	0.0040
Tnfrsf11a	ILMN_2482600	ILMN_191831	3.69	0.0000
Ramp3	ILMN_2749052	ILMN_222398	3.68	0.0000
LOC100045280	ILMN_2634591	ILMN_323613	3.68	0.0000
Apip	ILMN_2799945	ILMN_208725	3.68	0.0000
Tyms-ps	ILMN_2921303	ILMN_230689	3.68	0.0003
Ube2q2	ILMN_2749911	ILMN_211956	3.67	0.0002
Tor2a	ILMN_2519488	ILMN_195870	3.66	0.0095
Nfat5	ILMN_2732123	ILMN_221186	3.66	0.0000
1810033M07Rik	ILMN_2888940	ILMN_215397	3.66	0.0002
Rnaset2	ILMN_3154111	ILMN_242135	3.65	0.0000
Wbp7	ILMN_2699833	ILMN_231579	3.64	0.0005
Ighmbp2	ILMN_2860419	ILMN_218180	3.64	0.0001
Scgb1a1	ILMN_2749152	ILMN_222406	3.64	0.0049
Gja3	ILMN_2632678	ILMN_213018	3.64	0.0000
1810049H13Rik	ILMN_2680665	ILMN_217236	3.64	0.0000
Cbr2	ILMN_2756665	ILMN_222912	3.63	0.0052
Tef	ILMN_2715400	ILMN_219934	3.62	0.0001
Slc31a2	ILMN_2634656	ILMN_213199	3.62	0.0006
Ccrl1	ILMN_1251660	ILMN_213477	3.61	0.0000
H2-Ab1	ILMN_2631423	ILMN_209838	3.61	0.0000
Acsm3	ILMN_3075043	ILMN_248603	3.60	0.0001
A930016P21Rik	ILMN_1224980	ILMN_257215	3.59	0.0014
Ccdc98	ILMN_2758642	ILMN_223050	3.59	0.0032
Ghrh	ILMN_1253853	ILMN_213432	3.59	0.0002
Wwc2	ILMN_2685464	ILMN_217612	3.58	0.0000
9030221M09Rik	ILMN_2718350	ILMN_211066	3.58	0.0000
Tsc22d3	ILMN_3150811	ILMN_257708	3.58	0.0001
3200002M19Rik	ILMN_2711401	ILMN_247516	3.56	0.0003
EG634650	ILMN_2837493	ILMN_252479	3.56	0.0000
C4b	ILMN_3049559	ILMN_210539	3.56	0.0000
Gm904	ILMN_2921438	ILMN_260240	3.55	0.0010
Ceacam13	ILMN_2592257	ILMN_213925	3.55	0.0029
Ncapg2	ILMN_2770723	ILMN_246953	3.55	0.0000
4932414J04Rik	ILMN_1237480	ILMN_313033	3.54	0.0000
Irgm	ILMN_1234539	ILMN_213497	3.54	0.0000
Dnmt3b	ILMN_1252310	ILMN_236655	3.54	0.0002
Prmt6	ILMN_3007271	ILMN_208707	3.53	0.0006
Gsk3a	ILMN_3160897	ILMN_254737	3.53	0.0001
Polr3a	ILMN_1255559	ILMN_244669	3.53	0.0001
Calm5	ILMN_3160926	ILMN_235729	3.52	0.0045
Mov10l1	ILMN_2617219	ILMN_211569	3.52	0.0068
OTTMUSG00000015762	ILMN_2932993	ILMN_249322	3.52	0.0003
Wbscr27	ILMN_1235834	ILMN_194140	3.52	0.0024
Rbbp7	ILMN_2691192	ILMN_215499	3.52	0.0020
Eef1a2	ILMN_2645171	ILMN_214177	3.52	0.0010
Olfr410	ILMN_1225411	ILMN_219502	3.51	0.0040
Fbxo32	ILMN_2715893	ILMN_219973	3.51	0.0019
Cnbp	ILMN_2663780	ILMN_215804	3.50	0.0081

Sart3	ILMN_2800297	ILMN_222751	3.50	0.0000
Zfp13	ILMN_2877740	ILMN_191405	3.50	0.0008
Cys1	ILMN_1229578	ILMN_239582	3.49	0.0000
Chek2	ILMN_1247062	ILMN_213090	3.49	0.0000
Prune	ILMN_1252629	ILMN_213137	3.48	0.0000
Axl	ILMN_2651715	ILMN_214769	3.47	0.0000
2310036O22Rik	ILMN_1229716	ILMN_217389	3.47	0.0000
Rcl1	ILMN_1254929	ILMN_220432	3.47	0.0051
Slc38a2	ILMN_1244123	ILMN_217242	3.46	0.0000
Il18	ILMN_1236517	ILMN_223025	3.46	0.0007
Cyfip2	ILMN_2670713	ILMN_214228	3.45	0.0000
Olfr1156	ILMN_2738305	ILMN_221642	3.45	0.0000
Faah	ILMN_2657980	ILMN_215313	3.45	0.0002
Asah3l	ILMN_2629112	ILMN_212677	3.45	0.0023
Sel1l	ILMN_1244431	ILMN_258443	3.45	0.0047
Hipk3	ILMN_1253204	ILMN_222086	3.44	0.0000
Myh8	ILMN_2978038	ILMN_214541	3.44	0.0001
Scap	ILMN_2764143	ILMN_241129	3.44	0.0001
Cd63	ILMN_3128907	ILMN_214933	3.44	0.0001
Ube1c	ILMN_2879517	ILMN_194035	3.43	0.0010
Dap3	ILMN_2646230	ILMN_211847	3.43	0.0051
Prelp	ILMN_2739760	ILMN_221743	3.43	0.0002
Wdr45l	ILMN_1242852	ILMN_212583	3.43	0.0001
1810009A15Rik	ILMN_1221262	ILMN_218996	3.43	0.0049
LOC100044275	ILMN_1253678	ILMN_329235	3.43	0.0001
Phactr1	ILMN_2683499	ILMN_242055	3.42	0.0047
Senp7	ILMN_2706451	ILMN_259164	3.42	0.0007
Mbd3l2	ILMN_2609129	ILMN_210782	3.41	0.0000
Gmpr	ILMN_2602581	ILMN_210136	3.41	0.0000
Ovgp1	ILMN_2600928	ILMN_209968	3.40	0.0016
Bach1	ILMN_2741677	ILMN_221891	3.40	0.0017
Nrk	ILMN_1220907	ILMN_257470	3.40	0.0001
Tm9sf3	ILMN_2603545	ILMN_256422	3.39	0.0002
Emp1	ILMN_2642913	ILMN_213976	3.39	0.0003
C130090K23Rik	ILMN_3163255	ILMN_209707	3.39	0.0000
Rad51l3	ILMN_2755140	ILMN_208658	3.39	0.0029
Actn2	ILMN_2797061	ILMN_223487	3.39	0.0001
Wars	ILMN_3156343	ILMN_184424	3.38	0.0000
Echdc3	ILMN_1234901	ILMN_211941	3.38	0.0004
Rassf7	ILMN_2778094	ILMN_217400	3.38	0.0000
A830093I24Rik	ILMN_1255757	ILMN_223276	3.37	0.0047
Tada3l	ILMN_2489694	ILMN_192612	3.37	0.0000
Dhps	ILMN_2637241	ILMN_234246	3.37	0.0012
Nup155	ILMN_2695281	ILMN_253387	3.37	0.0000
Snapc2	ILMN_1215174	ILMN_217540	3.37	0.0045
Il1rl1	ILMN_2757617	ILMN_215312	3.37	0.0000
Hivep1	ILMN_2657800	ILMN_215299	3.36	0.0000
Nrbp1	ILMN_1229702	ILMN_208786	3.36	0.0017
Csf2	ILMN_2749412	ILMN_222423	3.35	0.0004
Wwc2	ILMN_2748806	ILMN_217612	3.35	0.0017

Hist2h2ab	ILMN_2785454	ILMN_196734	3.34	0.0000
Mfap5	ILMN_1225835	ILMN_233307	3.34	0.0011
Slc38a9	ILMN_1242435	ILMN_217616	3.34	0.0004
Clspn	ILMN_2858359	ILMN_212127	3.34	0.0098
Syt12	ILMN_1246560	ILMN_219798	3.34	0.0004
Arl6ip4	ILMN_3034691	ILMN_221776	3.34	0.0000
V1rj2	ILMN_2503699	ILMN_194132	3.33	0.0066
Ric3	ILMN_2624193	ILMN_212228	3.32	0.0002
LOC100045252	ILMN_2692980	ILMN_317035	3.32	0.0005
Utp11l	ILMN_2720334	ILMN_220301	3.32	0.0001
Zfp655	ILMN_1219539	ILMN_209693	3.32	0.0000
Bnipl	ILMN_2834971	ILMN_211723	3.32	0.0027
Pafah1b1	ILMN_2640401	ILMN_209398	3.31	0.0001
Rac2	ILMN_2600678	ILMN_209944	3.31	0.0000
Slitrk1	ILMN_2961282	ILMN_260619	3.31	0.0000
Ckm	ILMN_2608804	ILMN_210754	3.30	0.0000
Car14	ILMN_2973824	ILMN_208706	3.30	0.0009
Rpl4	ILMN_2631003	ILMN_212850	3.30	0.0005
Centb5	ILMN_1236232	ILMN_210009	3.30	0.0033
Ch25h	ILMN_2702303	ILMN_218948	3.29	0.0012
Rfxdc1	ILMN_2734616	ILMN_221366	3.29	0.0010
Col14a1	ILMN_2591027	ILMN_208943	3.29	0.0003
Stk19	ILMN_1232972	ILMN_214568	3.29	0.0000
Usp33	ILMN_3129526	ILMN_242196	3.29	0.0000
LOC100048046	ILMN_2740176	ILMN_318424	3.29	0.0000
Tmem121	ILMN_1231884	ILMN_216549	3.28	0.0006
Myc	ILMN_2623526	ILMN_210055	3.28	0.0000
Chkb	ILMN_2755021	ILMN_257429	3.28	0.0001
Rabgap1	ILMN_3061844	ILMN_246037	3.28	0.0006
Mlx	ILMN_2620510	ILMN_211894	3.28	0.0005
Flot2	ILMN_2688439	ILMN_211586	3.28	0.0006
Map2k7	ILMN_3102163	ILMN_234235	3.27	0.0001
Ldb3	ILMN_3136283	ILMN_235875	3.27	0.0036
Mad2l1bp	ILMN_1221616	ILMN_216260	3.27	0.0005
Igfbp6	ILMN_2689790	ILMN_217958	3.27	0.0012
Snapc1	ILMN_2745849	ILMN_222181	3.25	0.0009
Slc2a6	ILMN_2618918	ILMN_211726	3.25	0.0002
Pik3r1	ILMN_3114641	ILMN_256560	3.25	0.0002
Taf5l	ILMN_2700751	ILMN_218829	3.24	0.0006
Pafah1b3	ILMN_2640971	ILMN_213804	3.24	0.0007
C4a	ILMN_2911128	ILMN_241215	3.23	0.0052
Ddt	ILMN_2596297	ILMN_209491	3.23	0.0080
Pop5	ILMN_2973925	ILMN_213331	3.22	0.0000
Zfp143	ILMN_1214416	ILMN_246559	3.22	0.0001
Plekhb1	ILMN_2933022	ILMN_221623	3.22	0.0002
Uhrf1bp1l	ILMN_1258677	ILMN_251894	3.21	0.0002
Snx17	ILMN_2694926	ILMN_218375	3.21	0.0004
Sept6	ILMN_2663914	ILMN_212389	3.21	0.0036
Ptch2	ILMN_2728710	ILMN_220926	3.20	0.0000
Ddb1	ILMN_2620930	ILMN_211937	3.20	0.0001

Grasp	ILMN_2656031	ILMN_209500	3.20	0.0002
Scmh1	ILMN_2720786	ILMN_247166	3.20	0.0002
Alas2	ILMN_2675874	ILMN_216832	3.19	0.0002
Saps3	ILMN_2698353	ILMN_218636	3.19	0.0007
Rex2	ILMN_3062349	ILMN_256827	3.19	0.0009
Tmem45a	ILMN_2982663	ILMN_211317	3.18	0.0002
Psmd2	ILMN_2682945	ILMN_217415	3.18	0.0010
Kcnh3	ILMN_2726397	ILMN_220756	3.18	0.0000
Pde4dip	ILMN_1215884	ILMN_228537	3.18	0.0013
Vegfa	ILMN_3121255	ILMN_224512	3.18	0.0020
Il7	ILMN_2630852	ILMN_212833	3.18	0.0002
Rtn1	ILMN_2758070	ILMN_221348	3.18	0.0017
Gsta2	ILMN_1248849	ILMN_196593	3.17	0.0002
Myl6	ILMN_2900910	ILMN_261689	3.17	0.0005
Cebpg	ILMN_2972026	ILMN_234754	3.17	0.0001
Prmt2	ILMN_3099751	ILMN_239396	3.16	0.0002
Dusp16	ILMN_3112011	ILMN_232573	3.16	0.0002
Smc1a	ILMN_1220530	ILMN_210942	3.16	0.0002
C630011I23	ILMN_2636990	ILMN_213430	3.16	0.0000
Hibch	ILMN_2635150	ILMN_213247	3.15	0.0012
B230339H12Rik	ILMN_2628744	ILMN_209162	3.15	0.0033
Camk1	ILMN_2944939	ILMN_216496	3.15	0.0034
Azi2	ILMN_3082580	ILMN_240407	3.15	0.0005
Pik3c2g	ILMN_3082346	ILMN_232457	3.14	0.0000
Ankfn1	ILMN_3127224	ILMN_254549	3.14	0.0022
Htr2a	ILMN_2725529	ILMN_214572	3.14	0.0016
A530023O14Rik	ILMN_2689651	ILMN_216995	3.13	0.0002
Noto	ILMN_2833082	ILMN_227181	3.13	0.0006
Zfp488	ILMN_2808383	ILMN_255922	3.12	0.0036
Sftpc	ILMN_2638865	ILMN_213605	3.12	0.0022
Tmem9b	ILMN_1246495	ILMN_214538	3.11	0.0003
Ksr1	ILMN_2849036	ILMN_211298	3.11	0.0000
Lmf1	ILMN_2655144	ILMN_215065	3.11	0.0001
Bmp4	ILMN_1215252	ILMN_223427	3.11	0.0001
Mospd1	ILMN_3006930	ILMN_220553	3.10	0.0019
Il18rap	ILMN_1221620	ILMN_218950	3.10	0.0006
Thap3	ILMN_2652425	ILMN_214829	3.10	0.0000
Tsc22d3	ILMN_2701664	ILMN_217937	3.10	0.0002
Ubl4b	ILMN_2741322	ILMN_221861	3.10	0.0004
Acot8	ILMN_2738629	ILMN_215852	3.09	0.0027
Iqgap1	ILMN_2654495	ILMN_192065	3.09	0.0002
Stag1	ILMN_1233273	ILMN_216336	3.08	0.0001
Myo5b	ILMN_2610576	ILMN_210919	3.08	0.0000
Cnp	ILMN_2802263	ILMN_222860	3.07	0.0028
Rabgap1	ILMN_3139344	ILMN_246037	3.07	0.0072
Scara5	ILMN_3008068	ILMN_222294	3.06	0.0007
Faim	ILMN_2632976	ILMN_213047	3.06	0.0029
Nme5	ILMN_2696232	ILMN_256278	3.06	0.0004
AI747448	ILMN_1253045	ILMN_248596	3.06	0.0017
Sorbs3	ILMN_2618221	ILMN_211656	3.06	0.0000

Pik3r1	ILMN_2473531	ILMN_244014	3.06	0.0003
Bnc2	ILMN_2885206	ILMN_220395	3.05	0.0001
1810010M01Rik	ILMN_2728429	ILMN_220906	3.05	0.0096
Dctn2	ILMN_2859659	ILMN_217096	3.05	0.0000
Cdc42ep3	ILMN_2733185	ILMN_224538	3.05	0.0002
Esrrb	ILMN_1228497	ILMN_213635	3.05	0.0092
Cilp	ILMN_2634430	ILMN_213174	3.05	0.0027
Dlgap2	ILMN_2937486	ILMN_211933	3.05	0.0000
Kcnc4	ILMN_1242025	ILMN_215081	3.04	0.0029
Frmd6	ILMN_2828916	ILMN_253678	3.03	0.0002
Ryr1	ILMN_2730425	ILMN_226638	3.03	0.0027
Eml1	ILMN_1214866	ILMN_261917	3.03	0.0030
Frat1	ILMN_1251022	ILMN_215812	3.03	0.0000
Nqo2	ILMN_2968731	ILMN_208782	3.03	0.0005
Mustn1	ILMN_2658461	ILMN_215350	3.03	0.0003
Fnip1	ILMN_2725155	ILMN_227968	3.03	0.0002
Hmgcs2	ILMN_1216322	ILMN_219260	3.02	0.0079
Csnk1g2	ILMN_2875159	ILMN_212758	3.02	0.0033
D9Ertd392e	ILMN_1227277	ILMN_193217	3.02	0.0046
Tnni3k	ILMN_3096551	ILMN_193974	3.01	0.0076
Capns1	ILMN_2772248	ILMN_223956	3.01	0.0079
Mpv17l	ILMN_2794258	ILMN_199364	3.01	0.0065
Slc9a6	ILMN_2892678	ILMN_246900	3.01	0.0040
Slc35b2	ILMN_1246740	ILMN_253509	3.01	0.0002
Clec9a	ILMN_2929768	ILMN_223116	3.00	0.0000
Prrx1	ILMN_2614463	ILMN_211302	3.00	0.0011
Synj2bp	ILMN_1252068	ILMN_218270	2.99	0.0000
Nudt21	ILMN_2979052	ILMN_215791	2.99	0.0072
Lor	ILMN_1241825	ILMN_218790	2.99	0.0007
Olfr1157	ILMN_2694128	ILMN_218302	2.99	0.0037
Rfxank	ILMN_3125181	ILMN_228492	2.98	0.0033
Ppp6c	ILMN_1242053	ILMN_230422	2.98	0.0006
Olfr195	ILMN_1247436	ILMN_214501	2.98	0.0014
4930402E16Rik	ILMN_2696146	ILMN_249392	2.98	0.0014
Oxt	ILMN_2669486	ILMN_216295	2.98	0.0014
Ankrd46	ILMN_1222794	ILMN_211567	2.98	0.0095
V1rc22	ILMN_2484864	ILMN_191341	2.98	0.0003
Pax2	ILMN_2949326	ILMN_211210	2.98	0.0000
Ggcx	ILMN_1249282	ILMN_212986	2.97	0.0038
Ccdc51	ILMN_1248125	ILMN_220092	2.97	0.0009
Klrb1b	ILMN_2646358	ILMN_196749	2.96	0.0045
Srm	ILMN_2809611	ILMN_211754	2.96	0.0020
Zbtb22	ILMN_1233129	ILMN_190228	2.96	0.0004
Churc1	ILMN_2797689	ILMN_250242	2.96	0.0049
2300002D11Rik	ILMN_1253600	ILMN_260547	2.95	0.0010
Slc1a4	ILMN_2888552	ILMN_213958	2.94	0.0017
Trim33	ILMN_2514776	ILMN_188197	2.94	0.0060
Smap2	ILMN_1222682	ILMN_213072	2.94	0.0007
Cdk2	ILMN_2770759	ILMN_256716	2.94	0.0008
Ipmk	ILMN_2938390	ILMN_233236	2.94	0.0064

Camk1d	ILMN_1220441	ILMN_253301	2.93	0.0087
Tpr	ILMN_2502996	ILMN_194060	2.93	0.0048
Olfr1100	ILMN_1242841	ILMN_223641	2.93	0.0034
Col17a1	ILMN_1216228	ILMN_221356	2.93	0.0007
St6galnac6	ILMN_3131666	ILMN_215086	2.93	0.0044
Svil	ILMN_2662644	ILMN_215439	2.93	0.0017
4931440F15Rik	ILMN_2789611	ILMN_218541	2.92	0.0001
Sirt3	ILMN_2737480	ILMN_221583	2.92	0.0002
Crim2	ILMN_2907964	ILMN_253999	2.92	0.0012
Abca4	ILMN_2728706	ILMN_211540	2.91	0.0039
Kcna7	ILMN_2619156	ILMN_211751	2.91	0.0005
Naca	ILMN_2651642	ILMN_214762	2.91	0.0003
Ranbp6	ILMN_2614861	ILMN_211344	2.91	0.0003
Plec1	ILMN_2599783	ILMN_209851	2.91	0.0002
Hnrpu	ILMN_1259775	ILMN_210101	2.90	0.0070
Bcl2l11	ILMN_1229255	ILMN_222050	2.90	0.0051
Glrb	ILMN_1232399	ILMN_209702	2.90	0.0026
Hs2st1	ILMN_2853601	ILMN_243423	2.90	0.0041
Olfr347	ILMN_1242663	ILMN_220320	2.90	0.0055
Hpx	ILMN_2830333	ILMN_222100	2.89	0.0009
Impdh1	ILMN_1216301	ILMN_214886	2.89	0.0005
Dclre1c	ILMN_1240123	ILMN_216472	2.89	0.0001
Mark1	ILMN_1236611	ILMN_257245	2.89	0.0000
Vil1	ILMN_2518406	ILMN_195755	2.88	0.0036
Sec61a1	ILMN_3162476	ILMN_219727	2.88	0.0066
Ankrd13a	ILMN_2613696	ILMN_259897	2.88	0.0084
Rad51c	ILMN_2748321	ILMN_247761	2.87	0.0003
Cpb2	ILMN_2905902	ILMN_208958	2.87	0.0005
Nkx2-1	ILMN_1260212	ILMN_208673	2.87	0.0015
Cd63	ILMN_3052430	ILMN_214933	2.87	0.0010
Tm2d3	ILMN_2608574	ILMN_208638	2.87	0.0030
Olfr1339	ILMN_2752937	ILMN_229602	2.87	0.0048
Rcor2	ILMN_1214713	ILMN_221340	2.86	0.0036
D11Wsu47e	ILMN_2700833	ILMN_218836	2.86	0.0023
BC018242	ILMN_2757716	ILMN_222987	2.86	0.0015
Sep15	ILMN_1249836	ILMN_251840	2.86	0.0024
Zfp273	ILMN_1232296	ILMN_250512	2.86	0.0012
Prss8	ILMN_2846148	ILMN_221829	2.86	0.0069
Kcnk5	ILMN_2982781	ILMN_218669	2.85	0.0041
Thoc3	ILMN_2888342	ILMN_222955	2.84	0.0014
Gm1821	ILMN_3157823	ILMN_243605	2.84	0.0009
Ptprr	ILMN_2665459	ILMN_215941	2.84	0.0009
Gm129	ILMN_3102736	ILMN_243152	2.84	0.0040
Aasdh	ILMN_1221418	ILMN_222321	2.84	0.0058
Cnot6l	ILMN_2642671	ILMN_220697	2.83	0.0041
Phldb1	ILMN_2615534	ILMN_211406	2.83	0.0008
Ankrd23	ILMN_2741117	ILMN_221842	2.83	0.0058
LOC100044159	ILMN_2599145	ILMN_320773	2.83	0.0073
9430041O17Rik	ILMN_1236413	ILMN_212896	2.82	0.0011
Tenc1	ILMN_2744164	ILMN_208700	2.82	0.0062

Tegt	ILMN_2697248	ILMN_218556	2.82	0.0004
Dhx35	ILMN_2861225	ILMN_220803	2.82	0.0057
Tmem102	ILMN_1245382	ILMN_256383	2.82	0.0051
Tacc2	ILMN_2738739	ILMN_244476	2.81	0.0000
Olfr676	ILMN_1256438	ILMN_220059	2.81	0.0005
Bri3	ILMN_2700334	ILMN_218796	2.80	0.0001
Htatip2	ILMN_2603837	ILMN_210264	2.80	0.0015
Trim44	ILMN_1231217	ILMN_189102	2.80	0.0006
Nnmt	ILMN_2885277	ILMN_215936	2.80	0.0042
Txlnb	ILMN_2886610	ILMN_211914	2.80	0.0021
Klf7	ILMN_1251426	ILMN_225175	2.79	0.0066
Pou2f1	ILMN_3127178	ILMN_218154	2.79	0.0030
Sbno2	ILMN_2638333	ILMN_213561	2.79	0.0035
Gng2	ILMN_3143604	ILMN_236937	2.79	0.0085
Lin54	ILMN_2793616	ILMN_215463	2.79	0.0013
EG317677	ILMN_2929594	ILMN_213670	2.79	0.0026
Slc12a3	ILMN_2756550	ILMN_254166	2.79	0.0002
Ddhd1	ILMN_3163148	ILMN_249923	2.78	0.0014
Pgpep1	ILMN_2846255	ILMN_209658	2.78	0.0079
Zfp202	ILMN_1223591	ILMN_192577	2.78	0.0005
Nsun5	ILMN_2796028	ILMN_210282	2.78	0.0000
Usp16	ILMN_2491301	ILMN_192783	2.78	0.0014
Zfp428	ILMN_1228583	ILMN_240402	2.78	0.0083
Boc	ILMN_1241005	ILMN_223229	2.78	0.0001
Zfp324	ILMN_2687195	ILMN_238165	2.78	0.0000
Tardbp	ILMN_3029953	ILMN_211677	2.77	0.0030
Pik3ap1	ILMN_2590736	ILMN_208912	2.77	0.0008
Pfn3	ILMN_2610314	ILMN_210897	2.77	0.0006
Vgll2	ILMN_2909248	ILMN_185749	2.76	0.0061
Adra1b	ILMN_2606415	ILMN_190038	2.76	0.0043
Kng2	ILMN_3126522	ILMN_199376	2.76	0.0025
Wnt10b	ILMN_2422848	ILMN_184938	2.76	0.0029
Tmem162	ILMN_1233860	ILMN_219091	2.76	0.0001
Usp25	ILMN_1226508	ILMN_189583	2.76	0.0092
Hcrt	ILMN_2592983	ILMN_209147	2.76	0.0006
Hdc	ILMN_2965903	ILMN_242858	2.76	0.0062
Clec4a3	ILMN_2606619	ILMN_210538	2.75	0.0031
Serpinb12	ILMN_3140610	ILMN_220564	2.75	0.0030
Ube2q1	ILMN_1247833	ILMN_191561	2.75	0.0002
Epdr1	ILMN_1225494	ILMN_214840	2.75	0.0066
Trim35	ILMN_2445989	ILMN_187656	2.74	0.0023
Eif5a	ILMN_2686044	ILMN_217074	2.74	0.0029
Orc4l	ILMN_2689513	ILMN_209879	2.73	0.0016
Slc35e3	ILMN_2619574	ILMN_247158	2.73	0.0010
Gtrgeo22	ILMN_2592095	ILMN_209052	2.73	0.0042
Glycam1	ILMN_2640849	ILMN_213794	2.73	0.0023
Olfr473	ILMN_1219139	ILMN_221584	2.73	0.0004
Theg	ILMN_2749394	ILMN_222422	2.72	0.0008
Figla	ILMN_2854992	ILMN_215962	2.72	0.0009
LOC100041585	ILMN_1249590	ILMN_317487	2.72	0.0010

Aatf	ILMN_3122864	ILMN_212076	2.72	0.0028
Rab12	ILMN_1232851	ILMN_247938	2.71	0.0098
Lonrf1	ILMN_3111383	ILMN_228296	2.71	0.0062
D130059P03Rik	ILMN_2709787	ILMN_208640	2.71	0.0022
Tpsab1	ILMN_2705860	ILMN_219221	2.70	0.0037
Hsd11b2	ILMN_2731265	ILMN_239314	2.70	0.0036
9930023K05Rik	ILMN_1250913	ILMN_216333	2.70	0.0032
Btn2a2	ILMN_1216723	ILMN_213941	2.69	0.0002
Zmynd19	ILMN_1257785	ILMN_191630	2.69	0.0028
Pkd2	ILMN_2866327	ILMN_220788	2.69	0.0030
Lnpep	ILMN_1242150	ILMN_209487	2.68	0.0018
Ykt6	ILMN_1246405	ILMN_219090	2.67	0.0031
2410015M20Rik	ILMN_1230931	ILMN_210022	2.67	0.0033
Pnrc2	ILMN_2861335	ILMN_221033	2.67	0.0015
LOC100044829	ILMN_1215147	ILMN_310045	2.66	0.0069
Atp5s	ILMN_2642158	ILMN_213913	2.66	0.0064
Clec1b	ILMN_2749280	ILMN_210411	2.66	0.0031
Pnpo	ILMN_2855792	ILMN_216152	2.66	0.0049
Elmo2	ILMN_3135409	ILMN_207476	2.66	0.0003
Timm8a1	ILMN_2896552	ILMN_223570	2.66	0.0019
Tbx20	ILMN_2601336	ILMN_210011	2.66	0.0027
Nup133	ILMN_2719266	ILMN_214967	2.66	0.0002
B230339M05Rik	ILMN_2894574	ILMN_260273	2.65	0.0006
C330027C09Rik	ILMN_2647961	ILMN_214430	2.65	0.0019
Mtnr1a	ILMN_1256388	ILMN_218773	2.65	0.0009
9530066K23Rik	ILMN_2844642	ILMN_209760	2.65	0.0050
Lin7c	ILMN_2669869	ILMN_253981	2.64	0.0018
Paip2	ILMN_1258515	ILMN_212185	2.64	0.0050
AW551984	ILMN_2721980	ILMN_220435	2.64	0.0026
Bmpr1b	ILMN_1216262	ILMN_209303	2.63	0.0024
Hrsp12	ILMN_2610703	ILMN_210108	2.63	0.0079
Pcdhgc3	ILMN_2890002	ILMN_236578	2.62	0.0018
Rab5b	ILMN_3163335	ILMN_260688	2.62	0.0060
Usp1	ILMN_1240950	ILMN_185240	2.62	0.0045
Tctn3	ILMN_1224799	ILMN_210189	2.62	0.0001
Sucla2	ILMN_2596942	ILMN_241666	2.61	0.0051
Tmem184a	ILMN_1242101	ILMN_214733	2.61	0.0048
Scp2	ILMN_1221684	ILMN_211436	2.60	0.0027
Epn2	ILMN_3158060	ILMN_235437	2.60	0.0043
AW111922	ILMN_2612776	ILMN_210056	2.60	0.0010
BC043934	ILMN_1253937	ILMN_219229	2.60	0.0011
Cog4	ILMN_1228129	ILMN_213878	2.59	0.0058
Alg9	ILMN_2609495	ILMN_210816	2.59	0.0049
Commd1	ILMN_1215639	ILMN_219841	2.58	0.0037
Asah1	ILMN_1231966	ILMN_211541	2.58	0.0006
Tcf20	ILMN_2763781	ILMN_201560	2.58	0.0010
Dnmbp	ILMN_1218651	ILMN_220754	2.58	0.0018
Snx14	ILMN_2619249	ILMN_211759	2.58	0.0046
Trex1	ILMN_2509737	ILMN_249588	2.58	0.0066
Slc12a6	ILMN_1213275	ILMN_214309	2.58	0.0010

Flt1	ILMN_2732721	ILMN_219641	2.57	0.0020
Chst8	ILMN_2643049	ILMN_213991	2.57	0.0019
Sparcl1	ILMN_1237917	ILMN_215660	2.57	0.0021
AA467197	ILMN_3160750	ILMN_236731	2.57	0.0010
Tctn3	ILMN_2932474	ILMN_210189	2.57	0.0017
Gm711	ILMN_2641920	ILMN_213893	2.57	0.0023
Gnaq	ILMN_2658443	ILMN_230615	2.56	0.0029
Rps24	ILMN_2625060	ILMN_237874	2.56	0.0093
Gfra3	ILMN_3102260	ILMN_211590	2.56	0.0075
Traf3	ILMN_3144289	ILMN_186921	2.56	0.0062
LOC235033	ILMN_2533387	ILMN_198917	2.56	0.0058
Whdc1	ILMN_3160616	ILMN_234611	2.56	0.0078
Neu4	ILMN_2605810	ILMN_210458	2.56	0.0021
Mgea6	ILMN_1259488	ILMN_199381	2.56	0.0005
A730008L03Rik	ILMN_2873174	ILMN_215122	2.55	0.0023
Slc10a6	ILMN_2636624	ILMN_213394	2.54	0.0074
Dom3z	ILMN_1233838	ILMN_214924	2.53	0.0014
OTTMUSG00000015529	ILMN_1238850	ILMN_223581	2.53	0.0009
Pde4dip	ILMN_3064283	ILMN_228537	2.53	0.0033
Gsdmc1	ILMN_1240230	ILMN_199368	2.53	0.0029
Sult5a1	ILMN_2731020	ILMN_221092	2.52	0.0026
Dars	ILMN_2589477	ILMN_208784	2.52	0.0070
Tmed10	ILMN_3002943	ILMN_239113	2.52	0.0016
Ifng	ILMN_2791459	ILMN_217631	2.52	0.0074
Hpcal4	ILMN_2662054	ILMN_215652	2.52	0.0060
Cyp2b10	ILMN_2594926	ILMN_230152	2.52	0.0007
Wdr62	ILMN_1241754	ILMN_249747	2.52	0.0004
Nipsnap3a	ILMN_2657376	ILMN_209109	2.52	0.0036
Btbd3	ILMN_2599955	ILMN_209090	2.52	0.0051
Pms2	ILMN_2707947	ILMN_219373	2.51	0.0005
Tbc1d22a	ILMN_2767791	ILMN_223698	2.51	0.0005
Rassf4	ILMN_2956095	ILMN_213302	2.51	0.0057
Hsd17b11	ILMN_2705361	ILMN_213984	2.51	0.0035
Ddef2	ILMN_3046047	ILMN_239548	2.51	0.0099
Adprhl1	ILMN_1257998	ILMN_209406	2.50	0.0028
Dusp21	ILMN_1213939	ILMN_251757	2.50	0.0061
Epn2	ILMN_1218487	ILMN_235437	2.49	0.0011
Cugbp1	ILMN_2726496	ILMN_220762	2.49	0.0016
1200013P24Rik	ILMN_1245322	ILMN_219220	2.48	0.0008
Olfr459	ILMN_1252811	ILMN_213676	2.48	0.0043
Adfp	ILMN_1222246	ILMN_223620	2.48	0.0033
Calcrl	ILMN_1235390	ILMN_209412	2.47	0.0082
Slc1a1	ILMN_1225873	ILMN_208829	2.47	0.0011
Raet1e	ILMN_1231358	ILMN_201534	2.47	0.0001
4930544G21Rik	ILMN_2841307	ILMN_243701	2.46	0.0026
Lims2	ILMN_2942674	ILMN_221324	2.46	0.0081
Zfp791	ILMN_2990485	ILMN_234512	2.46	0.0066
Scgb3a1	ILMN_2658200	ILMN_215331	2.46	0.0050
Mid1	ILMN_3080065	ILMN_242848	2.46	0.0037
Cd2	ILMN_2753697	ILMN_222706	2.46	0.0065

Slc14a1	ILMN_1235006	ILMN_246276	2.46	0.0087
2610109H07Rik	ILMN_3018201	ILMN_236248	2.45	0.0056
Vps16	ILMN_2420505	ILMN_184664	2.45	0.0034
Stag2	ILMN_3123970	ILMN_257297	2.45	0.0066
Abcf1	ILMN_2760415	ILMN_218252	2.45	0.0081
Birc2	ILMN_1226186	ILMN_240777	2.44	0.0042
4732418C07Rik	ILMN_2692060	ILMN_215708	2.43	0.0061
Mon1a	ILMN_2607297	ILMN_210601	2.43	0.0072
Kcnh2	ILMN_1244402	ILMN_213233	2.43	0.0032
2010100O12Rik	ILMN_2670427	ILMN_214419	2.43	0.0053
Cd83	ILMN_2865016	ILMN_215128	2.43	0.0085
Olfr43	ILMN_2589171	ILMN_208751	2.43	0.0014
Rbm6	ILMN_1252216	ILMN_212268	2.42	0.0036
Aup1	ILMN_2682623	ILMN_234129	2.42	0.0079
Ms4a5	ILMN_2726615	ILMN_220769	2.42	0.0016
Actr1b	ILMN_3161327	ILMN_230692	2.42	0.0054
B430211C08Rik	ILMN_2901576	ILMN_244026	2.42	0.0045
Ccdc127	ILMN_2932814	ILMN_214991	2.41	0.0034
Tsc22d1	ILMN_2677878	ILMN_216991	2.41	0.0019
Gzmn	ILMN_1229426	ILMN_196570	2.41	0.0009
Palm2	ILMN_2670330	ILMN_210672	2.41	0.0041
Sfmbt2	ILMN_2659651	ILMN_193443	2.41	0.0041
Ubb	ILMN_2516699	ILMN_195563	2.40	0.0080
Brd2	ILMN_2751505	ILMN_252658	2.40	0.0014
Srr	ILMN_2622605	ILMN_212090	2.40	0.0033
Hes6	ILMN_2608043	ILMN_210674	2.40	0.0010
Pigt	ILMN_2868131	ILMN_252543	2.40	0.0088
Casp9	ILMN_1217061	ILMN_212974	2.39	0.0015
Fabp6	ILMN_2826304	ILMN_222022	2.39	0.0016
Pou5f1	ILMN_1252910	ILMN_216259	2.39	0.0073
V1rg8	ILMN_1256776	ILMN_184774	2.38	0.0081
LOC100048332	ILMN_2717496	ILMN_317679	2.38	0.0096
8030451F13Rik	ILMN_2739266	ILMN_256951	2.38	0.0069
Slc5a10	ILMN_1222016	ILMN_228817	2.37	0.0062
Mmp12	ILMN_1250421	ILMN_255926	2.37	0.0056
Steap4	ILMN_2657297	ILMN_209928	2.37	0.0055
Prkcdbp	ILMN_1243602	ILMN_214335	2.37	0.0022
Timm22	ILMN_2589359	ILMN_208771	2.37	0.0038
Hfe2	ILMN_2688092	ILMN_217829	2.36	0.0021
EG434858	ILMN_3084883	ILMN_233946	2.36	0.0012
Errfi1	ILMN_2714031	ILMN_246139	2.36	0.0086
Sil1	ILMN_2720930	ILMN_220348	2.36	0.0081
Pmm2	ILMN_2858742	ILMN_261882	2.36	0.0013
Ift122	ILMN_2429970	ILMN_185784	2.35	0.0045
Mfge8	ILMN_3133448	ILMN_228582	2.34	0.0079
Olfr1263	ILMN_1231085	ILMN_216791	2.33	0.0018
Bmp10	ILMN_2935098	ILMN_255512	2.33	0.0062
Olfr1080	ILMN_1248928	ILMN_216797	2.33	0.0018
Kctd15	ILMN_2655869	ILMN_215130	2.31	0.0046
V1rg4	ILMN_2460929	ILMN_189399	2.31	0.0086

LOC100048221	ILMN_2748584	ILMN_314100	2.31	0.0043
Gp38	ILMN_2654754	ILMN_215032	2.31	0.0038
2310042D19Rik	ILMN_2957987	ILMN_217547	2.30	0.0094
Zfp597	ILMN_3160688	ILMN_260846	2.29	0.0086
3300001P08Rik	ILMN_2798797	ILMN_213598	2.28	0.0069
Adora1	ILMN_3065420	ILMN_238585	2.28	0.0064
2610020H08Rik	ILMN_2743616	ILMN_222027	2.28	0.0008
Zpbp2	ILMN_2757923	ILMN_211909	2.28	0.0052
B930076A02	ILMN_1214060	ILMN_210034	2.27	0.0027
Bhmt	ILMN_2620994	ILMN_225088	2.27	0.0007
AA673488	ILMN_3059135	ILMN_239401	2.27	0.0003
EG237009	ILMN_2739355	ILMN_321979	2.27	0.0010
Zbbx	ILMN_2707676	ILMN_219361	2.27	0.0008
C9	ILMN_2594846	ILMN_209337	2.26	0.0015
9830002I17Rik	ILMN_2732757	ILMN_221228	2.26	0.0096
Sntb2	ILMN_1217790	ILMN_218207	2.26	0.0084
Spata3	ILMN_2706809	ILMN_219287	2.25	0.0089
Ank3	ILMN_1223963	ILMN_252240	2.24	0.0011
Apoa2	ILMN_1247156	ILMN_216506	2.24	0.0082
Fgfbp3	ILMN_2628999	ILMN_212665	2.23	0.0025
9030227G01Rik	ILMN_2686433	ILMN_209828	2.23	0.0058
Obrgrp	ILMN_2603568	ILMN_210235	2.23	0.0049
Adam4	ILMN_1235132	ILMN_220039	2.23	0.0012
Asph	ILMN_2776700	ILMN_209310	2.22	0.0096
Pcdh1	ILMN_2597660	ILMN_209630	2.22	0.0067
Nup155	ILMN_1235035	ILMN_253387	2.22	0.0029
Oca2	ILMN_2475689	ILMN_191055	2.22	0.0033
5730577I03Rik	ILMN_2794628	ILMN_222559	2.21	0.0060
Gipc1	ILMN_1254924	ILMN_209294	2.21	0.0018
Pabpc1	ILMN_2705407	ILMN_201529	2.21	0.0080
Pank1	ILMN_2868013	ILMN_222263	2.21	0.0056
Col4a3bp	ILMN_2613636	ILMN_261277	2.20	0.0037
Ccr10	ILMN_1230042	ILMN_212484	2.19	0.0087
Rfc3	ILMN_2726047	ILMN_220736	2.19	0.0014
Pgls	ILMN_2664548	ILMN_215862	2.19	0.0057
Lce1b	ILMN_2872965	ILMN_216467	2.18	0.0035
MGC107098	ILMN_2922284	ILMN_255921	2.18	0.0046
Sgk2	ILMN_2827081	ILMN_215694	2.17	0.0076
Tfpt	ILMN_2877706	ILMN_212710	2.17	0.0030
Serpina1a	ILMN_2777493	ILMN_230055	2.17	0.0067
Kctd6	ILMN_1247335	ILMN_219998	2.17	0.0066
Olfr1454	ILMN_2947960	ILMN_218191	2.16	0.0040
Hmx3	ILMN_2790983	ILMN_214402	2.16	0.0086
Rpl14	ILMN_1227142	ILMN_216057	2.16	0.0021
1810015C04Rik	ILMN_2745614	ILMN_222164	2.15	0.0084
Slc12a3	ILMN_1229114	ILMN_254166	2.15	0.0028
Nkiras1	ILMN_2656189	ILMN_214595	2.14	0.0058
Dhdds	ILMN_2691067	ILMN_213516	2.13	0.0073
Atp1b4	ILMN_2768612	ILMN_223752	2.13	0.0009
Bcl11a	ILMN_2712044	ILMN_228019	2.13	0.0066

Ifi27	ILMN_2762944	ILMN_239930	2.13	0.0089
Slc11a1	ILMN_2692797	ILMN_254692	2.13	0.0033
Nkx2-5	ILMN_2727695	ILMN_220848	2.12	0.0012
LOC229571	ILMN_2894763	ILMN_229890	2.12	0.0001
Mfn1	ILMN_2950837	ILMN_220222	2.12	0.0098
Abca1	ILMN_1226851	ILMN_215646	2.11	0.0035
Zfp111	ILMN_2836698	ILMN_187476	2.11	0.0042
Lefty2	ILMN_2713164	ILMN_219748	2.10	0.0066
Ebi2	ILMN_1220101	ILMN_223731	2.10	0.0047
Hgf	ILMN_2729117	ILMN_220957	2.10	0.0053
Prdx5	ILMN_2637233	ILMN_224724	2.08	0.0087
Isl2	ILMN_1243489	ILMN_221871	2.06	0.0042
BC025816	ILMN_2697238	ILMN_218555	2.05	0.0060
Zc3h3	ILMN_2507227	ILMN_185104	2.04	0.0030
Olfr137	ILMN_1241505	ILMN_218146	2.03	0.0063
Akap3	ILMN_2614990	ILMN_211355	2.03	0.0090
Phxr5	ILMN_2712756	ILMN_327634	1.99	0.0063
Csnk2a1	ILMN_1223808	ILMN_215755	1.99	0.0069
Dnali1	ILMN_2872896	ILMN_253245	1.96	0.0035
Bcl2l1	ILMN_2742557	ILMN_214423	1.96	0.0079
Adarb1	ILMN_3036678	ILMN_247455	1.96	0.0076
Rnf167	ILMN_1214800	ILMN_217477	1.93	0.0088
Sema6d	ILMN_1258953	ILMN_217215	1.92	0.0089
Pknox2	ILMN_2663139	ILMN_254436	1.92	0.0012
Saa3	ILMN_2772632	ILMN_254030	1.92	0.0093
Megf11	ILMN_2618651	ILMN_232739	1.88	0.0093
Chst3	ILMN_2864172	ILMN_215951	1.85	0.0087
Ccdc18	ILMN_1257698	ILMN_241253	1.79	0.0081
LOC100046264	ILMN_2727142	ILMN_314590	-1.82	0.0095
Htr4	ILMN_2743188	ILMN_259080	-1.83	0.0045
Atxn3	ILMN_2813284	ILMN_211348	-1.83	0.0061
Slc7a15	ILMN_3163103	ILMN_215929	-1.84	0.0098
Cdca7	ILMN_1221568	ILMN_216604	-1.86	0.0064
Prkca	ILMN_1217890	ILMN_223295	-1.87	0.0092
Sept6	ILMN_2992517	ILMN_212389	-1.89	0.0048
Klhl31	ILMN_3161736	ILMN_216835	-1.91	0.0029
Upf3b	ILMN_2494625	ILMN_193163	-1.94	0.0048
Rab11b	ILMN_2678592	ILMN_220447	-1.94	0.0037
2210038L17Rik	ILMN_2809656	ILMN_258785	-1.96	0.0040
Pbx1	ILMN_1256907	ILMN_246200	-1.96	0.0080
LOC100044927	ILMN_2512023	ILMN_316485	-2.00	0.0067
LOC100048589	ILMN_1227874	ILMN_310316	-2.01	0.0039
Olfr25	ILMN_1239971	ILMN_244357	-2.02	0.0041
Chtf18	ILMN_1232175	ILMN_222121	-2.03	0.0093
E2f6	ILMN_2723931	ILMN_220573	-2.03	0.0068
Nxph2	ILMN_1251624	ILMN_216723	-2.03	0.0076
Ilk	ILMN_2662428	ILMN_215687	-2.04	0.0048
5630401D24Rik	ILMN_2740461	ILMN_221329	-2.06	0.0017
Cyp11b2	ILMN_2748181	ILMN_222335	-2.06	0.0080
Wscd1	ILMN_2610442	ILMN_210909	-2.06	0.0073

Tub	ILMN_1220143	ILMN_190171	-2.06	0.0088
Palm	ILMN_2815626	ILMN_210997	-2.06	0.0091
2010107G12Rik	ILMN_2975295	ILMN_245127	-2.06	0.0063
Olfr575	ILMN_2791541	ILMN_219706	-2.08	0.0024
BC049730	ILMN_2907915	ILMN_258440	-2.09	0.0099
Pofut2	ILMN_2615547	ILMN_245334	-2.10	0.0091
Vps13c	ILMN_2479134	ILMN_191445	-2.11	0.0079
Efna5	ILMN_2662536	ILMN_235749	-2.11	0.0073
Kcnk2	ILMN_2651369	ILMN_211329	-2.12	0.0042
Dmpk	ILMN_1229944	ILMN_252780	-2.12	0.0042
Stat1	ILMN_2655721	ILMN_254778	-2.12	0.0048
Treml2	ILMN_2980379	ILMN_254312	-2.13	0.0056
4831426I19Rik	ILMN_3156782	ILMN_217788	-2.14	0.0077
Avp	ILMN_2710928	ILMN_219592	-2.14	0.0016
Frag1	ILMN_2751492	ILMN_214081	-2.14	0.0032
Zkscan1	ILMN_2744259	ILMN_248708	-2.14	0.0048
Nuak1	ILMN_2928720	ILMN_234029	-2.15	0.0073
Ppt2	ILMN_2616827	ILMN_211539	-2.16	0.0028
Rerg	ILMN_1230648	ILMN_212208	-2.16	0.0066
Crem	ILMN_2747264	ILMN_233527	-2.17	0.0083
Tspan11	ILMN_2843163	ILMN_216944	-2.17	0.0041
Mmel1	ILMN_1253943	ILMN_218296	-2.18	0.0049
Lrdd	ILMN_2992009	ILMN_220270	-2.18	0.0012
Hist3h2a	ILMN_2629289	ILMN_241136	-2.18	0.0056
Prox1	ILMN_2598766	ILMN_209740	-2.19	0.0034
Pex12	ILMN_1250747	ILMN_212145	-2.20	0.0008
Speer5-ps1	ILMN_2748533	ILMN_222365	-2.20	0.0026
Ube2l3	ILMN_2475727	ILMN_251120	-2.20	0.0067
Camkk1	ILMN_1242310	ILMN_216860	-2.21	0.0045
Ppil2	ILMN_2771686	ILMN_256781	-2.21	0.0009
Tob1	ILMN_1250011	ILMN_191002	-2.21	0.0045
Sall1	ILMN_2853800	ILMN_214029	-2.22	0.0054
Asph	ILMN_2610810	ILMN_209310	-2.22	0.0069
Tgfb1i1	ILMN_1227722	ILMN_216486	-2.22	0.0089
Gorasp2	ILMN_1238654	ILMN_210814	-2.23	0.0036
9630028B13Rik	ILMN_2857095	ILMN_216918	-2.23	0.0024
Kif22	ILMN_2762326	ILMN_223310	-2.23	0.0073
Cml5	ILMN_2659237	ILMN_215418	-2.24	0.0017
Atp11c	ILMN_3152506	ILMN_238244	-2.24	0.0029
Lrsam1	ILMN_2761459	ILMN_212097	-2.24	0.0038
Il15	ILMN_1219869	ILMN_225654	-2.24	0.0072
Lrch4	ILMN_2597210	ILMN_209585	-2.25	0.0011
D19Ertd737e	ILMN_1249292	ILMN_214140	-2.25	0.0026
4930453N24Rik	ILMN_2888766	ILMN_248901	-2.25	0.0089
5830405N20Rik	ILMN_2690214	ILMN_217992	-2.26	0.0027
Hsf4	ILMN_2746797	ILMN_222242	-2.26	0.0068
Klra2	ILMN_1251941	ILMN_222820	-2.26	0.0049
Dct	ILMN_1251894	ILMN_219109	-2.27	0.0054
Nkx2-3	ILMN_2697760	ILMN_218592	-2.27	0.0069
Cpox	ILMN_2739182	ILMN_215021	-2.27	0.0015

Clec5a	ILMN_3045984	ILMN_259534	-2.27	0.0048
Rnf139	ILMN_2604560	ILMN_210338	-2.27	0.0009
Phex	ILMN_1243830	ILMN_217699	-2.28	0.0066
LOC100046080	ILMN_1217480	ILMN_309891	-2.28	0.0011
Rilp	ILMN_1218228	ILMN_226905	-2.28	0.0081
LOC381852	ILMN_2830750	ILMN_252182	-2.28	0.0031
Kcnj6	ILMN_3038066	ILMN_240501	-2.29	0.0013
Gstt3	ILMN_2665715	ILMN_215961	-2.29	0.0044
Efcab4b	ILMN_1240788	ILMN_231789	-2.29	0.0010
Gad1	ILMN_2621743	ILMN_212011	-2.30	0.0002
Ppan	ILMN_2778530	ILMN_249668	-2.30	0.0015
Acsl3	ILMN_3061300	ILMN_250023	-2.30	0.0061
Mafk	ILMN_2683831	ILMN_217490	-2.30	0.0053
Chchd2	ILMN_2833786	ILMN_221127	-2.30	0.0010
Yif1a	ILMN_2959787	ILMN_185962	-2.31	0.0009
Tnfaip1	ILMN_2429800	ILMN_185763	-2.31	0.0033
Arfgap1	ILMN_2908547	ILMN_211960	-2.32	0.0022
Sms	ILMN_1232323	ILMN_184683	-2.32	0.0003
Lmnb1	ILMN_2720229	ILMN_220293	-2.33	0.0026
Rasef	ILMN_2950343	ILMN_235059	-2.34	0.0009
Olfr873	ILMN_3160657	ILMN_238978	-2.34	0.0072
Tacr1	ILMN_2726850	ILMN_208724	-2.34	0.0024
Cenpk	ILMN_2668436	ILMN_216205	-2.35	0.0046
P2ry10	ILMN_2954781	ILMN_213782	-2.35	0.0014
Svil	ILMN_1224247	ILMN_215439	-2.35	0.0018
Psg16	ILMN_2611851	ILMN_249548	-2.35	0.0028
Kcnj6	ILMN_3162725	ILMN_252608	-2.35	0.0003
Galnt10	ILMN_2878781	ILMN_212503	-2.36	0.0053
Gnal	ILMN_3036452	ILMN_254487	-2.36	0.0059
Sae1	ILMN_2513895	ILMN_185054	-2.36	0.0009
Shroom3	ILMN_3162511	ILMN_240281	-2.36	0.0021
Myo7b	ILMN_2626106	ILMN_212410	-2.36	0.0006
Hist3h2ba	ILMN_2652022	ILMN_214796	-2.37	0.0008
Derl1	ILMN_2896416	ILMN_212566	-2.37	0.0028
Pdcd1	ILMN_1260060	ILMN_213210	-2.37	0.0021
MGC117608	ILMN_2999032	ILMN_234681	-2.37	0.0018
Ank3	ILMN_2622307	ILMN_212062	-2.38	0.0030
Olfr1040	ILMN_1237824	ILMN_214655	-2.38	0.0046
Plb1	ILMN_3036004	ILMN_258284	-2.38	0.0037
Cer1	ILMN_2884751	ILMN_259043	-2.38	0.0065
Nfe2l3	ILMN_2787209	ILMN_223567	-2.39	0.0041
Nphp4	ILMN_2641152	ILMN_213822	-2.40	0.0029
Otop2	ILMN_2700317	ILMN_218795	-2.41	0.0019
Cmklr1	ILMN_2774056	ILMN_233212	-2.41	0.0028
Trpv6	ILMN_3001186	ILMN_195673	-2.41	0.0029
Clk2	ILMN_2885016	ILMN_208789	-2.41	0.0030
Tlr3	ILMN_2697002	ILMN_218534	-2.42	0.0030
Hs3st6	ILMN_3160653	ILMN_233041	-2.42	0.0012
Ifna6	ILMN_1242716	ILMN_196765	-2.43	0.0074
Rnf8	ILMN_1214110	ILMN_213016	-2.43	0.0004

Mgst3	ILMN_1238479	ILMN_210134	-2.43	0.0077
Ifne1	ILMN_1255754	ILMN_223661	-2.43	0.0033
E130309D02Rik	ILMN_1251585	ILMN_219074	-2.43	0.0008
Nbn	ILMN_1240392	ILMN_215143	-2.44	0.0012
Ifngr2	ILMN_1258300	ILMN_209117	-2.44	0.0072
Armcx6	ILMN_3012253	ILMN_249108	-2.45	0.0006
Ifrg15	ILMN_2767764	ILMN_223695	-2.45	0.0034
Il1r1	ILMN_1231125	ILMN_217023	-2.46	0.0066
AB112350	ILMN_2902492	ILMN_211687	-2.47	0.0064
Srp9	ILMN_1260348	ILMN_213903	-2.47	0.0099
Muc5ac	ILMN_2884000	ILMN_231564	-2.47	0.0075
Stag3	ILMN_2993843	ILMN_212542	-2.48	0.0087
Ninj2	ILMN_2626283	ILMN_212424	-2.48	0.0007
Fhl4	ILMN_2793982	ILMN_217150	-2.49	0.0028
Ripk3	ILMN_2750356	ILMN_222495	-2.49	0.0013
Pik3c2a	ILMN_1252098	ILMN_220040	-2.49	0.0087
Rabggta	ILMN_1245469	ILMN_216278	-2.49	0.0007
EG244595	ILMN_2784942	ILMN_228041	-2.50	0.0080
Pomt1	ILMN_2662545	ILMN_215697	-2.50	0.0030
Spdef	ILMN_1258162	ILMN_222088	-2.50	0.0073
Dbndd1	ILMN_1227672	ILMN_214620	-2.50	0.0008
4930526D03Rik	ILMN_2858266	ILMN_239831	-2.50	0.0024
Khdrbs1	ILMN_1256052	ILMN_220721	-2.50	0.0051
Ccng1	ILMN_2500276	ILMN_232357	-2.50	0.0096
2010002N04Rik	ILMN_2706014	ILMN_219235	-2.51	0.0054
Ntn4	ILMN_2959863	ILMN_250922	-2.51	0.0047
Bcap29	ILMN_2866752	ILMN_214217	-2.51	0.0037
Lsm3	ILMN_2837865	ILMN_223070	-2.51	0.0040
LOC100040799	ILMN_3162394	ILMN_314797	-2.51	0.0004
Nasp	ILMN_1251930	ILMN_211691	-2.52	0.0098
Coro2b	ILMN_1216552	ILMN_209081	-2.52	0.0019
Rgp1	ILMN_2710589	ILMN_244100	-2.52	0.0071
BC005537	ILMN_1227295	ILMN_209456	-2.52	0.0098
Apol7b	ILMN_2887215	ILMN_258754	-2.52	0.0027
Twistnb	ILMN_1231779	ILMN_189979	-2.52	0.0001
Fbxw11	ILMN_2708319	ILMN_219399	-2.52	0.0098
Mybpc3	ILMN_2689136	ILMN_217905	-2.52	0.0081
Defb5	ILMN_1232541	ILMN_222434	-2.52	0.0012
Gpr109a	ILMN_2847115	ILMN_221500	-2.53	0.0041
Trip12	ILMN_2510364	ILMN_187207	-2.53	0.0070
Slc4a1ap	ILMN_2969435	ILMN_214013	-2.53	0.0087
LOC554292	ILMN_2941575	ILMN_238123	-2.53	0.0010
Pira11	ILMN_3073899	ILMN_252909	-2.53	0.0033
Anpep	ILMN_2589651	ILMN_208800	-2.54	0.0081
Tgm3	ILMN_2777363	ILMN_252846	-2.54	0.0036
Acbd4	ILMN_3150296	ILMN_217938	-2.54	0.0040
Col18a1	ILMN_2937542	ILMN_221410	-2.55	0.0014
Matn4	ILMN_2729289	ILMN_212683	-2.55	0.0042
Mobkl2c	ILMN_2661168	ILMN_215572	-2.56	0.0010
Olfr351	ILMN_2665824	ILMN_215969	-2.57	0.0029

3110050N22Rik	ILMN_1221011	ILMN_221927	-2.57	0.0032
Stat1	ILMN_2593196	ILMN_254778	-2.57	0.0054
Creb3l4	ILMN_1213549	ILMN_223420	-2.58	0.0037
Zfp192	ILMN_2463828	ILMN_189725	-2.58	0.0033
1810007E14Rik	ILMN_2671528	ILMN_216478	-2.58	0.0074
Gm597	ILMN_2973504	ILMN_252624	-2.58	0.0001
Glt25d2	ILMN_2889899	ILMN_223309	-2.58	0.0015
Phf6	ILMN_1237295	ILMN_211977	-2.58	0.0006
Adam2	ILMN_2739679	ILMN_221736	-2.58	0.0002
Ube2e1	ILMN_1231810	ILMN_191348	-2.59	0.0030
B2m	ILMN_2739999	ILMN_209318	-2.59	0.0046
H2-Q5	ILMN_2685581	ILMN_196752	-2.59	0.0033
Relt	ILMN_2422834	ILMN_184937	-2.59	0.0017
Cdyl2	ILMN_2728967	ILMN_220944	-2.59	0.0012
Sertad4	ILMN_1229745	ILMN_220872	-2.60	0.0020
E030010A14Rik	ILMN_2599686	ILMN_209836	-2.60	0.0015
Tmem49	ILMN_2861787	ILMN_209017	-2.60	0.0017
Agrp	ILMN_2703427	ILMN_245093	-2.61	0.0001
Armc6	ILMN_2873469	ILMN_230074	-2.61	0.0053
Pcdh10	ILMN_2663752	ILMN_215801	-2.61	0.0037
Dmkn	ILMN_3032088	ILMN_219849	-2.61	0.0005
Frs2	ILMN_2993711	ILMN_260176	-2.61	0.0019
Zfp42	ILMN_2961604	ILMN_189202	-2.62	0.0043
Etf1	ILMN_2995755	ILMN_222612	-2.62	0.0084
Cyp3a13	ILMN_2639214	ILMN_213639	-2.62	0.0034
Capn3	ILMN_2867771	ILMN_218422	-2.63	0.0086
Cul4b	ILMN_2656909	ILMN_215220	-2.63	0.0089
Fdxr	ILMN_1214585	ILMN_216640	-2.63	0.0026
Panx3	ILMN_2721052	ILMN_220361	-2.63	0.0009
Bbc3	ILMN_2690621	ILMN_212340	-2.63	0.0002
Col1a1	ILMN_2687872	ILMN_217812	-2.63	0.0044
Etv4	ILMN_2778111	ILMN_260771	-2.63	0.0009
2610021K21Rik	ILMN_2660136	ILMN_228893	-2.63	0.0025
2210010L05Rik	ILMN_2612238	ILMN_242384	-2.64	0.0023
Rab17	ILMN_2752490	ILMN_215232	-2.64	0.0061
Thyn1	ILMN_2987062	ILMN_211080	-2.64	0.0006
Hist1h3c	ILMN_1229755	ILMN_220453	-2.64	0.0042
Mak10	ILMN_2828599	ILMN_211089	-2.65	0.0080
Oas1g	ILMN_1253808	ILMN_212179	-2.65	0.0031
Mapk8	ILMN_2456617	ILMN_188893	-2.65	0.0029
St3gal3	ILMN_2879848	ILMN_211800	-2.65	0.0066
Vmo1	ILMN_2936105	ILMN_253414	-2.65	0.0065
Lca5	ILMN_3024416	ILMN_228057	-2.65	0.0053
Gabra3	ILMN_2595601	ILMN_209420	-2.65	0.0002
6430548M08Rik	ILMN_2599214	ILMN_188148	-2.66	0.0085
4933421E11Rik	ILMN_1221212	ILMN_233237	-2.66	0.0034
Samd14	ILMN_1235581	ILMN_251191	-2.66	0.0029
Rbm4b	ILMN_1220793	ILMN_233191	-2.66	0.0050
9330155M09Rik	ILMN_2765053	ILMN_223520	-2.67	0.0004
Cr2	ILMN_2696049	ILMN_208998	-2.67	0.0028

Ruvbl1	ILMN_1228999	ILMN_216028	-2.67	0.0000
Bai1	ILMN_2680188	ILMN_217198	-2.68	0.0075
BC024814	ILMN_2592909	ILMN_209139	-2.68	0.0056
Lyar	ILMN_2651126	ILMN_214718	-2.68	0.0029
ORF63	ILMN_1260025	ILMN_222699	-2.68	0.0001
9530002B09Rik	ILMN_1230372	ILMN_217820	-2.68	0.0046
Tcl1	ILMN_2985612	ILMN_213546	-2.68	0.0029
Ppap2b	ILMN_1247753	ILMN_212849	-2.68	0.0025
LOC100045268	ILMN_1248969	ILMN_310712	-2.69	0.0017
Gtpbp1	ILMN_2510196	ILMN_194850	-2.69	0.0019
Upf1	ILMN_2740755	ILMN_239726	-2.69	0.0026
Arg2	ILMN_2669164	ILMN_216267	-2.69	0.0060
scl33870.2_144	ILMN_2459676	ILMN_188833	-2.70	0.0025
EG368203	ILMN_2669886	ILMN_216335	-2.70	0.0030
Ier2	ILMN_2642705	ILMN_213956	-2.70	0.0049
Ptges	ILMN_2786442	ILMN_211451	-2.70	0.0051
D4Ertd22e	ILMN_3129206	ILMN_241928	-2.71	0.0030
Adamts20	ILMN_2598519	ILMN_209718	-2.71	0.0006
Gpr124	ILMN_1214180	ILMN_260422	-2.71	0.0003
Srrm2	ILMN_3028894	ILMN_217084	-2.72	0.0011
H2-DMa	ILMN_2866856	ILMN_224639	-2.72	0.0098
Rest	ILMN_2615160	ILMN_211371	-2.72	0.0012
Olfr1097	ILMN_2791018	ILMN_216528	-2.72	0.0028
Snf1lk	ILMN_1239463	ILMN_210221	-2.72	0.0003
Olfr433	ILMN_2862009	ILMN_225742	-2.72	0.0001
Trim6	ILMN_2940417	ILMN_227487	-2.72	0.0016
Col2a1	ILMN_2759142	ILMN_237764	-2.72	0.0004
Gpr133	ILMN_3123855	ILMN_244372	-2.73	0.0002
Dhx57	ILMN_2617925	ILMN_210546	-2.73	0.0014
Ga17	ILMN_1244685	ILMN_187273	-2.73	0.0042
Slc4a3	ILMN_2593621	ILMN_209211	-2.73	0.0038
Tap2	ILMN_2686721	ILMN_217715	-2.74	0.0019
Hoxd12	ILMN_2951852	ILMN_221822	-2.74	0.0010
Tbpl1	ILMN_2605872	ILMN_210463	-2.74	0.0028
Gabrb3	ILMN_3120335	ILMN_240761	-2.74	0.0031
V1rd17	ILMN_2417982	ILMN_184357	-2.74	0.0002
B230396O12Rik	ILMN_2806479	ILMN_245278	-2.74	0.0051
Clic4	ILMN_2761109	ILMN_259580	-2.74	0.0041
Ankrd1	ILMN_2625451	ILMN_212352	-2.75	0.0000
Mas1	ILMN_1253860	ILMN_215167	-2.77	0.0030
Dazl	ILMN_2634147	ILMN_213146	-2.77	0.0044
4933417A18Rik	ILMN_2873321	ILMN_223588	-2.77	0.0002
Alox12	ILMN_2827535	ILMN_211240	-2.78	0.0028
Plekha4	ILMN_1243826	ILMN_216745	-2.78	0.0028
Slc7a4	ILMN_2639360	ILMN_229227	-2.78	0.0036
2610200G18Rik	ILMN_2605041	ILMN_210379	-2.78	0.0002
Rab6ip1	ILMN_3033866	ILMN_213254	-2.78	0.0053
Pax3	ILMN_2634195	ILMN_213151	-2.79	0.0040
1700039E15Rik	ILMN_2958222	ILMN_259506	-2.79	0.0036
Klra15	ILMN_1233185	ILMN_196755	-2.79	0.0006

Fxyd5	ILMN_1235493	ILMN_221977	-2.79	0.0056
Ppap2b	ILMN_2630993	ILMN_212849	-2.79	0.0033
ENSMUSG00000068790	ILMN_3070389	ILMN_234056	-2.80	0.0046
LOC382106	ILMN_3131601	ILMN_246426	-2.80	0.0027
G6pc3	ILMN_1218602	ILMN_208870	-2.80	0.0007
1110004E09Rik	ILMN_1257567	ILMN_212399	-2.80	0.0059
Sts	ILMN_2650049	ILMN_214626	-2.81	0.0008
Rexo1	ILMN_2592554	ILMN_241596	-2.81	0.0009
2900024C23Rik	ILMN_2842802	ILMN_216218	-2.81	0.0011
Spsb4	ILMN_1243507	ILMN_212461	-2.81	0.0029
Dbi	ILMN_1215469	ILMN_223553	-2.81	0.0001
4921528G01Rik	ILMN_1247585	ILMN_192792	-2.81	0.0003
Lox	ILMN_2997494	ILMN_219336	-2.82	0.0060
Slc16a8	ILMN_2926057	ILMN_209899	-2.82	0.0001
2610208M17Rik	ILMN_3104094	ILMN_207453	-2.82	0.0064
Ank2	ILMN_2718030	ILMN_236030	-2.82	0.0043
A030010B05Rik	ILMN_1252682	ILMN_185939	-2.83	0.0002
Olfr1357	ILMN_2784096	ILMN_251498	-2.83	0.0012
4930563P21Rik	ILMN_1255963	ILMN_240965	-2.83	0.0002
Trpd52l3	ILMN_2989947	ILMN_233680	-2.83	0.0010
Mbd4	ILMN_1213981	ILMN_219494	-2.83	0.0009
Rbx1	ILMN_1230583	ILMN_210570	-2.84	0.0076
Map2k5	ILMN_2639375	ILMN_213658	-2.84	0.0037
Zswim4	ILMN_2457408	ILMN_188989	-2.84	0.0006
A830018L16Rik	ILMN_2616075	ILMN_211454	-2.84	0.0015
Klk1b1	ILMN_1256119	ILMN_196746	-2.84	0.0014
Clec4a3	ILMN_3161652	ILMN_210538	-2.85	0.0081
Ucn	ILMN_2834808	ILMN_196227	-2.85	0.0098
Dmrta1	ILMN_2712159	ILMN_219683	-2.85	0.0001
Dnm1l	ILMN_3111421	ILMN_256682	-2.85	0.0042
Senp5	ILMN_2978857	ILMN_212645	-2.85	0.0000
Prr15	ILMN_2649456	ILMN_214576	-2.85	0.0018
Epha7	ILMN_2826671	ILMN_210861	-2.86	0.0008
Olfr883	ILMN_2688153	ILMN_217834	-2.86	0.0002
Rtp1	ILMN_2855737	ILMN_230544	-2.86	0.0042
Nme4	ILMN_1244161	ILMN_210085	-2.86	0.0039
Nlk	ILMN_2683613	ILMN_217474	-2.86	0.0002
Calca	ILMN_3028637	ILMN_246924	-2.87	0.0021
Cc2d1a	ILMN_2645613	ILMN_214216	-2.87	0.0053
Slc15a1	ILMN_2943040	ILMN_222458	-2.88	0.0056
l7Rn6	ILMN_3162224	ILMN_210943	-2.88	0.0038
Auts2	ILMN_3052726	ILMN_211916	-2.88	0.0034
Cilp2	ILMN_2963720	ILMN_244613	-2.88	0.0002
Aatk	ILMN_1231439	ILMN_221321	-2.88	0.0024
Zfp157	ILMN_1235580	ILMN_237439	-2.88	0.0009
2310057J18Rik	ILMN_1220128	ILMN_210967	-2.88	0.0010
Zfp36l3	ILMN_1250130	ILMN_259745	-2.88	0.0000
Sypl	ILMN_2758720	ILMN_227266	-2.89	0.0039
Exdl2	ILMN_2927225	ILMN_208913	-2.89	0.0011
Nf2	ILMN_2611840	ILMN_252759	-2.89	0.0000

Zfat	ILMN_2985411	ILMN_201573	-2.89	0.0000
Srd5a1	ILMN_2692927	ILMN_218206	-2.89	0.0036
Cdkl2	ILMN_2657790	ILMN_215298	-2.90	0.0009
Man1a	ILMN_2715319	ILMN_219928	-2.90	0.0021
Rhox4d	ILMN_2846026	ILMN_243531	-2.90	0.0093
Ccdc96	ILMN_2886872	ILMN_222153	-2.91	0.0000
EG232599	ILMN_2605183	ILMN_210393	-2.91	0.0013
Mpl	ILMN_1226499	ILMN_213487	-2.92	0.0002
Slc16a7	ILMN_2645399	ILMN_214197	-2.92	0.0008
Gpr81	ILMN_2699134	ILMN_218703	-2.93	0.0011
Mbp	ILMN_3081854	ILMN_250782	-2.93	0.0037
E130303B06Rik	ILMN_2706129	ILMN_219244	-2.93	0.0005
Elf2	ILMN_2748336	ILMN_212428	-2.93	0.0010
Gm50	ILMN_3131846	ILMN_257862	-2.93	0.0057
Gbp3	ILMN_1244513	ILMN_216816	-2.93	0.0079
Eml5	ILMN_3114898	ILMN_259591	-2.94	0.0086
Bglap1	ILMN_2610166	ILMN_247195	-2.95	0.0006
Ctdp1	ILMN_2613659	ILMN_211213	-2.95	0.0001
Mybbp1a	ILMN_1216204	ILMN_228300	-2.96	0.0002
Serpinb3a	ILMN_2687408	ILMN_238870	-2.96	0.0037
Ebpl	ILMN_2725035	ILMN_211415	-2.96	0.0035
Pdzk1ip1	ILMN_2618935	ILMN_211728	-2.97	0.0048
4632404H12Rik	ILMN_2629897	ILMN_209340	-2.97	0.0015
Rreb1	ILMN_3122124	ILMN_249028	-2.97	0.0003
Cd248	ILMN_2896843	ILMN_216345	-2.97	0.0006
Slc43a3	ILMN_2687437	ILMN_214763	-2.97	0.0023
Grin2c	ILMN_2757484	ILMN_222972	-2.98	0.0056
Shf	ILMN_3163419	ILMN_245068	-3.00	0.0000
Col24a1	ILMN_2857666	ILMN_261313	-3.00	0.0005
Invs	ILMN_2664073	ILMN_212167	-3.00	0.0002
Csf2rb2	ILMN_2750047	ILMN_222474	-3.01	0.0003
Gpr137b	ILMN_3042112	ILMN_227353	-3.01	0.0002
Slc6a2	ILMN_2669736	ILMN_216320	-3.01	0.0021
Ctrl	ILMN_2673260	ILMN_216616	-3.01	0.0003
Ddah1	ILMN_1256676	ILMN_195643	-3.01	0.0009
Gpnmb	ILMN_1236420	ILMN_211323	-3.01	0.0000
Olfr727	ILMN_2741180	ILMN_221847	-3.02	0.0080
Chrna2	ILMN_2681916	ILMN_217328	-3.02	0.0005
Rnf138	ILMN_1220310	ILMN_217489	-3.02	0.0002
Slc1a2	ILMN_3047516	ILMN_225538	-3.02	0.0008
Gcnt2	ILMN_1227951	ILMN_210789	-3.02	0.0005
Prom2	ILMN_1246392	ILMN_214759	-3.02	0.0059
Psme4	ILMN_2947187	ILMN_217690	-3.03	0.0003
Atg3	ILMN_2595188	ILMN_209368	-3.03	0.0008
Olfr536	ILMN_1231381	ILMN_209923	-3.03	0.0097
Bag1	ILMN_2712018	ILMN_219670	-3.03	0.0000
Ldlrad3	ILMN_1217129	ILMN_213656	-3.03	0.0012
Zfp386	ILMN_3067404	ILMN_242298	-3.04	0.0001
Slain1	ILMN_2751572	ILMN_222565	-3.04	0.0036
Abcd1	ILMN_1245447	ILMN_209991	-3.04	0.0004

Trp53inp2	ILMN_2457585	ILMN_189011	-3.04	0.0024
Olfr131	ILMN_2997843	ILMN_211670	-3.04	0.0003
Leo1	ILMN_2903351	ILMN_249636	-3.04	0.0022
6430527G18Rik	ILMN_3109491	ILMN_230817	-3.05	0.0000
Il1rn	ILMN_3151270	ILMN_218418	-3.05	0.0001
Sphk2	ILMN_2718791	ILMN_218883	-3.06	0.0029
4933433P14Rik	ILMN_1237665	ILMN_212198	-3.06	0.0000
2610042L04Rik	ILMN_3059346	ILMN_226632	-3.06	0.0001
Katnal1	ILMN_2971577	ILMN_221323	-3.07	0.0024
Pof1b	ILMN_1242912	ILMN_221065	-3.07	0.0008
E130319B15Rik	ILMN_2590042	ILMN_225010	-3.07	0.0018
Olfr1364	ILMN_2612727	ILMN_211121	-3.07	0.0030
Ccl25	ILMN_2661722	ILMN_215623	-3.08	0.0000
Gnl3	ILMN_2673441	ILMN_210042	-3.09	0.0013
AU019823	ILMN_2875915	ILMN_239543	-3.09	0.0010
Acsm5	ILMN_1220520	ILMN_215322	-3.10	0.0040
Elk4	ILMN_1231726	ILMN_209814	-3.10	0.0015
Map1lc3a	ILMN_2832682	ILMN_211286	-3.11	0.0008
Dact1	ILMN_1230235	ILMN_242104	-3.11	0.0017
Clstn1	ILMN_2441501	ILMN_187140	-3.12	0.0020
Col24a1	ILMN_1231275	ILMN_261313	-3.12	0.0007
Pif1	ILMN_2714678	ILMN_219875	-3.12	0.0087
Stoml1	ILMN_1222000	ILMN_218804	-3.12	0.0000
Trps1	ILMN_2466527	ILMN_190025	-3.12	0.0025
Rspo3	ILMN_3162785	ILMN_221501	-3.12	0.0013
Nhsl1	ILMN_2744492	ILMN_222092	-3.12	0.0002
Gm884	ILMN_2838901	ILMN_252306	-3.13	0.0009
Htt	ILMN_2672091	ILMN_216523	-3.13	0.0081
Dhrs9	ILMN_2631251	ILMN_191346	-3.13	0.0000
Deadc1	ILMN_2705097	ILMN_219163	-3.13	0.0034
Nola1	ILMN_1226280	ILMN_222966	-3.13	0.0058
Fbxo7	ILMN_2612683	ILMN_211116	-3.14	0.0000
Ube2d2	ILMN_1215117	ILMN_189646	-3.15	0.0029
Eln	ILMN_2697304	ILMN_218560	-3.15	0.0000
Setd4	ILMN_2670361	ILMN_213178	-3.16	0.0014
Casr	ILMN_2588631	ILMN_208691	-3.17	0.0003
Slc9a10	ILMN_2867343	ILMN_232752	-3.18	0.0000
Slc37a3	ILMN_2722497	ILMN_219771	-3.19	0.0019
Lyzl4	ILMN_1232761	ILMN_219817	-3.19	0.0002
Adamts2	ILMN_1226259	ILMN_210818	-3.19	0.0065
Gpam	ILMN_2655954	ILMN_238446	-3.19	0.0012
BC005764	ILMN_2709001	ILMN_219445	-3.20	0.0003
Zfp238	ILMN_3049032	ILMN_245276	-3.20	0.0019
Robo4	ILMN_1219026	ILMN_186816	-3.20	0.0000
Traf3ip1	ILMN_2990070	ILMN_240687	-3.22	0.0063
BC021395	ILMN_2776876	ILMN_239185	-3.22	0.0058
Tnfsf13b	ILMN_1226099	ILMN_184661	-3.22	0.0001
Ccdc98	ILMN_2908022	ILMN_223050	-3.23	0.0001
Nedd9	ILMN_2654186	ILMN_211969	-3.23	0.0002
Prnp	ILMN_2619316	ILMN_211763	-3.23	0.0007

Lypla3	ILMN_1257060	ILMN_217194	-3.23	0.0045
Rnf208	ILMN_2636349	ILMN_213366	-3.23	0.0011
Hmx2	ILMN_3161049	ILMN_231208	-3.23	0.0002
Otof	ILMN_2674951	ILMN_214752	-3.23	0.0041
Cd44	ILMN_3114585	ILMN_245439	-3.24	0.0000
Cuzd1	ILMN_2592718	ILMN_209118	-3.24	0.0000
Aldh18a1	ILMN_3123473	ILMN_253613	-3.25	0.0021
Ptp4a2	ILMN_2725835	ILMN_210989	-3.25	0.0031
Zfp36l1	ILMN_1247853	ILMN_185448	-3.25	0.0001
Wnk1	ILMN_1241638	ILMN_208893	-3.25	0.0044
Slc7a10	ILMN_2705508	ILMN_219193	-3.25	0.0015
Zfp715	ILMN_2895084	ILMN_208684	-3.25	0.0008
Ppp2r5e	ILMN_2623244	ILMN_249253	-3.26	0.0021
Slc37a2	ILMN_2751120	ILMN_222539	-3.27	0.0008
Ercc8	ILMN_2719001	ILMN_209905	-3.27	0.0007
Gcnt1	ILMN_1256299	ILMN_214513	-3.27	0.0007
Olfr975	ILMN_2625468	ILMN_212353	-3.27	0.0050
Gtse1	ILMN_2908070	ILMN_215955	-3.28	0.0000
Foxh1	ILMN_2659741	ILMN_215454	-3.28	0.0024
Edg6	ILMN_2839479	ILMN_210265	-3.29	0.0001
2810416G20Rik	ILMN_2988818	ILMN_230931	-3.29	0.0013
Il12a	ILMN_2706462	ILMN_242787	-3.30	0.0001
Gm815	ILMN_2975865	ILMN_248037	-3.30	0.0002
Disc1	ILMN_3118664	ILMN_230984	-3.30	0.0000
EG633640	ILMN_2977390	ILMN_238251	-3.30	0.0024
Maml3	ILMN_2950067	ILMN_237161	-3.32	0.0000
Def6	ILMN_2595842	ILMN_209446	-3.33	0.0000
Als2cr4	ILMN_3135801	ILMN_246204	-3.33	0.0001
Lcn8	ILMN_2733045	ILMN_221254	-3.33	0.0012
Ash2l	ILMN_2485900	ILMN_192198	-3.33	0.0001
Sox9	ILMN_3007428	ILMN_261259	-3.34	0.0001
Dgkz	ILMN_2915060	ILMN_210741	-3.34	0.0014
Psmd2	ILMN_2682947	ILMN_217415	-3.34	0.0000
Pdzd3	ILMN_1258691	ILMN_216198	-3.34	0.0012
Wdr8	ILMN_2432829	ILMN_186126	-3.34	0.0013
LOC100048638	ILMN_1249743	ILMN_313088	-3.35	0.0000
LOC625360	ILMN_2845658	ILMN_257506	-3.35	0.0002
Rasa2	ILMN_2715328	ILMN_237560	-3.35	0.0026
Nrip3	ILMN_2875404	ILMN_217092	-3.35	0.0014
Il8rb	ILMN_2737157	ILMN_221556	-3.35	0.0012
Krr1	ILMN_1218250	ILMN_230837	-3.35	0.0009
Cab39l	ILMN_1217943	ILMN_213138	-3.36	0.0000
Hectd2	ILMN_1252222	ILMN_211710	-3.36	0.0002
Reck	ILMN_2812614	ILMN_231246	-3.36	0.0000
6330403K07Rik	ILMN_2594139	ILMN_209264	-3.36	0.0004
OTTMUSG00000000421	ILMN_2824954	ILMN_234919	-3.36	0.0002
Olfr232	ILMN_2830349	ILMN_227449	-3.37	0.0004
Bcorl1	ILMN_2755424	ILMN_222829	-3.37	0.0001
Olfr876	ILMN_2640485	ILMN_257982	-3.37	0.0000
Olfr1189	ILMN_1242368	ILMN_214482	-3.38	0.0000

Smcr7	ILMN_2995370	ILMN_236162	-3.38	0.0033
LOC100046207	ILMN_2748897	ILMN_315638	-3.39	0.0001
Exosc2	ILMN_2683326	ILMN_196609	-3.39	0.0004
Psmc5	ILMN_1251877	ILMN_214862	-3.40	0.0013
D5Ertd135e	ILMN_2950270	ILMN_224382	-3.40	0.0000
Saa2	ILMN_2758571	ILMN_223044	-3.40	0.0018
B4galnt4	ILMN_2789862	ILMN_233892	-3.40	0.0006
Pex10	ILMN_2941122	ILMN_244327	-3.40	0.0017
Zfyve19	ILMN_2826947	ILMN_188987	-3.40	0.0068
Duox2	ILMN_2622260	ILMN_230561	-3.41	0.0080
Olfr913	ILMN_1232331	ILMN_255917	-3.41	0.0004
Fgfr1	ILMN_2727068	ILMN_216804	-3.41	0.0003
Ctdsp1	ILMN_1213307	ILMN_212383	-3.42	0.0005
4930504E06Rik	ILMN_2630827	ILMN_212829	-3.42	0.0028
Hspa1a	ILMN_2829594	ILMN_257431	-3.42	0.0003
Ipo7	ILMN_2619968	ILMN_238304	-3.42	0.0001
Ank1	ILMN_2609011	ILMN_208704	-3.42	0.0086
Npffr2	ILMN_3145557	ILMN_220560	-3.42	0.0001
Kdelr3	ILMN_2675697	ILMN_240560	-3.42	0.0002
AA408296	ILMN_2631230	ILMN_211104	-3.43	0.0002
Zpld1	ILMN_2735680	ILMN_221441	-3.43	0.0032
Rpl23	ILMN_1238227	ILMN_214598	-3.44	0.0014
Pik3r3	ILMN_1226157	ILMN_212250	-3.44	0.0003
Dsp	ILMN_2654997	ILMN_255543	-3.44	0.0002
Myog	ILMN_2863674	ILMN_212019	-3.44	0.0008
Cdkn1a	ILMN_2846775	ILMN_209664	-3.45	0.0001
Pcdhga7	ILMN_1252556	ILMN_210424	-3.45	0.0015
Mark3	ILMN_3134078	ILMN_245400	-3.46	0.0020
A630077B13Rik	ILMN_1249864	ILMN_220279	-3.46	0.0000
Agpat5	ILMN_1250939	ILMN_212222	-3.47	0.0002
Kcnk1	ILMN_3009501	ILMN_237297	-3.47	0.0000
Mapk7	ILMN_1232294	ILMN_217900	-3.47	0.0000
Stxbp1	ILMN_2644632	ILMN_214135	-3.49	0.0000
Olfr194	ILMN_3161142	ILMN_257433	-3.50	0.0031
Rorc	ILMN_2760272	ILMN_223173	-3.50	0.0053
Gpr82	ILMN_1238020	ILMN_221259	-3.51	0.0095
Cdipt	ILMN_1224421	ILMN_210695	-3.52	0.0040
Mtvr2	ILMN_3108203	ILMN_252009	-3.52	0.0000
Afmid	ILMN_2600443	ILMN_209564	-3.52	0.0000
Gramd1c	ILMN_2616382	ILMN_211488	-3.53	0.0000
Pcgf6	ILMN_1251165	ILMN_222533	-3.54	0.0014
Siah1b	ILMN_2903734	ILMN_211639	-3.54	0.0001
Hexb	ILMN_2829330	ILMN_221504	-3.54	0.0000
Nap1l1	ILMN_3004970	ILMN_214137	-3.54	0.0000
Fgd4	ILMN_2610084	ILMN_210876	-3.55	0.0006
Tmeff2	ILMN_2935998	ILMN_194817	-3.56	0.0000
Dolpp1	ILMN_2966370	ILMN_220141	-3.56	0.0000
Gpx6	ILMN_2792028	ILMN_233571	-3.57	0.0000
Six2	ILMN_2742627	ILMN_221958	-3.57	0.0000
Mtap1s	ILMN_2657432	ILMN_215270	-3.57	0.0001

1700123L14Rik	ILMN_2925702	ILMN_228979	-3.57	0.0001
Pcdha6	ILMN_3155884	ILMN_208968	-3.57	0.0000
1200002N14Rik	ILMN_1219820	ILMN_213609	-3.58	0.0000
Spib	ILMN_2933168	ILMN_258029	-3.58	0.0017
Cdk2	ILMN_1233064	ILMN_256716	-3.58	0.0000
Zmiz1	ILMN_1224736	ILMN_258289	-3.59	0.0001
Bnc2	ILMN_2721466	ILMN_220395	-3.59	0.0000
Cxx1a	ILMN_2952779	ILMN_226958	-3.59	0.0008
Gabrp	ILMN_1221640	ILMN_212083	-3.60	0.0001
Msh3	ILMN_3068452	ILMN_232099	-3.60	0.0000
Obscn	ILMN_2693176	ILMN_226087	-3.60	0.0004
Rab11fip2	ILMN_2933151	ILMN_238924	-3.61	0.0030
Olfr103	ILMN_1244967	ILMN_219546	-3.61	0.0000
Stx2	ILMN_2657575	ILMN_215282	-3.62	0.0029
Olfr1366	ILMN_2629280	ILMN_212694	-3.62	0.0002
Vti1b	ILMN_2503190	ILMN_194080	-3.62	0.0003
Aym1	ILMN_3162325	ILMN_235462	-3.62	0.0030
Sf3b4	ILMN_3051392	ILMN_211099	-3.63	0.0029
Mogat2	ILMN_2854920	ILMN_211610	-3.63	0.0014
Ikzf1	ILMN_3127790	ILMN_229247	-3.63	0.0000
Osbpl1a	ILMN_2711676	ILMN_215509	-3.64	0.0000
AB182283	ILMN_2891085	ILMN_240594	-3.65	0.0003
Prlr	ILMN_2617005	ILMN_210332	-3.67	0.0002
Appbp1	ILMN_2955356	ILMN_212180	-3.68	0.0000
Tbc1d10b	ILMN_3008497	ILMN_237726	-3.68	0.0002
Krt18	ILMN_2711267	ILMN_219615	-3.68	0.0015
Mdga2	ILMN_2722424	ILMN_214652	-3.68	0.0030
Cplx2	ILMN_1238436	ILMN_219945	-3.69	0.0002
Zfp90	ILMN_2487482	ILMN_192371	-3.71	0.0000
Cnot2	ILMN_3162796	ILMN_242279	-3.71	0.0019
Es1	ILMN_2704749	ILMN_218872	-3.72	0.0016
1700123D08Rik	ILMN_2863712	ILMN_242440	-3.72	0.0017
Orai2	ILMN_2624293	ILMN_212237	-3.73	0.0000
Rsbn1	ILMN_2589576	ILMN_208791	-3.73	0.0001
5330421F07Rik	ILMN_2592597	ILMN_209106	-3.74	0.0044
Fcrl1	ILMN_2608151	ILMN_210682	-3.74	0.0000
Rnase6	ILMN_2759309	ILMN_223096	-3.74	0.0014
Mansc1	ILMN_2848273	ILMN_212652	-3.74	0.0002
Dclre1b	ILMN_2669813	ILMN_224631	-3.74	0.0005
Pstpip2	ILMN_2722513	ILMN_232180	-3.74	0.0005
D12Ertd551e	ILMN_1240131	ILMN_211409	-3.75	0.0000
Olfr1330	ILMN_2864972	ILMN_217835	-3.75	0.0000
Cnih	ILMN_1237640	ILMN_228365	-3.75	0.0000
Syt10	ILMN_2724250	ILMN_220601	-3.76	0.0014
Arl4d	ILMN_2593709	ILMN_209218	-3.76	0.0007
Dhrs9	ILMN_2602089	ILMN_261046	-3.76	0.0001
Asb6	ILMN_1254737	ILMN_213066	-3.76	0.0001
Igsf1	ILMN_2759192	ILMN_223086	-3.76	0.0000
Slfn3	ILMN_2908122	ILMN_222964	-3.77	0.0053
Siglec1	ILMN_2762966	ILMN_223354	-3.77	0.0002

Chchd3	ILMN_1243188	ILMN_216695	-3.80	0.0000
Sh3pxd2a	ILMN_1227918	ILMN_211991	-3.80	0.0000
Acpp	ILMN_2693019	ILMN_218213	-3.81	0.0000
Elf5	ILMN_2736380	ILMN_216298	-3.81	0.0000
4930432O21Rik	ILMN_3006178	ILMN_241163	-3.81	0.0000
Tcl1b3	ILMN_2921240	ILMN_231961	-3.82	0.0056
V1rh6	ILMN_2867574	ILMN_236849	-3.82	0.0000
Trpm3	ILMN_2427909	ILMN_255895	-3.83	0.0009
Scmh1	ILMN_1230766	ILMN_247166	-3.84	0.0000
Lman1l	ILMN_1212980	ILMN_214284	-3.85	0.0000
Psip1	ILMN_2617478	ILMN_211417	-3.85	0.0000
Hoxa9	ILMN_2772762	ILMN_210756	-3.85	0.0001
Copg	ILMN_1233848	ILMN_236440	-3.85	0.0007
Cacng5	ILMN_1226821	ILMN_217419	-3.85	0.0012
Nexn	ILMN_2669215	ILMN_211900	-3.86	0.0000
Slc25a3	ILMN_2751046	ILMN_222536	-3.86	0.0000
Olfr42	ILMN_2592893	ILMN_209137	-3.86	0.0000
AI661453	ILMN_2830520	ILMN_214565	-3.87	0.0002
Upp1	ILMN_2959293	ILMN_187510	-3.87	0.0009
Hrasls5	ILMN_2999683	ILMN_218409	-3.87	0.0000
Dab2ip	ILMN_2615559	ILMN_233597	-3.88	0.0000
Grk5	ILMN_2637571	ILMN_212032	-3.88	0.0000
Gjb1	ILMN_2948476	ILMN_219510	-3.90	0.0006
Hmgn3	ILMN_1247704	ILMN_214141	-3.90	0.0003
Psg19	ILMN_1227375	ILMN_213508	-3.90	0.0000
Zfp78	ILMN_1260400	ILMN_232784	-3.90	0.0000
2810002I04Rik	ILMN_2711181	ILMN_219611	-3.92	0.0000
Olfr518	ILMN_2673414	ILMN_224849	-3.92	0.0008
Syvn1	ILMN_2628258	ILMN_212597	-3.92	0.0070
Cntn4	ILMN_2710524	ILMN_216888	-3.94	0.0000
Lyzs	ILMN_2939681	ILMN_261574	-3.95	0.0036
Gtse1	ILMN_1226369	ILMN_215955	-3.95	0.0056
Zmat5	ILMN_2690772	ILMN_218036	-3.95	0.0000
Hemgn	ILMN_1239447	ILMN_212528	-3.95	0.0046
Ceacam16	ILMN_1225476	ILMN_247696	-3.96	0.0000
Capn6	ILMN_2695143	ILMN_218388	-3.96	0.0000
LOC100047670	ILMN_1254554	ILMN_310142	-3.96	0.0010
Zfp423	ILMN_2469366	ILMN_190347	-3.96	0.0086
Ern2	ILMN_1214511	ILMN_209279	-3.97	0.0012
Hecw2	ILMN_3098718	ILMN_257854	-3.97	0.0000
Prdm14	ILMN_3037312	ILMN_246565	-3.97	0.0079
EG218444	ILMN_2682702	ILMN_217392	-3.98	0.0000
Atmin	ILMN_2701824	ILMN_218909	-3.99	0.0062
Stra13	ILMN_2836023	ILMN_215353	-3.99	0.0001
Pipox	ILMN_2916202	ILMN_217942	-3.99	0.0000
Es22	ILMN_2627528	ILMN_212535	-4.01	0.0000
1110038F14Rik	ILMN_2629375	ILMN_212705	-4.01	0.0000
Creld2	ILMN_2983948	ILMN_222963	-4.02	0.0000
Fa2h	ILMN_2746783	ILMN_222241	-4.03	0.0000
Tcfap2b	ILMN_2760450	ILMN_217594	-4.04	0.0000

Ccdc66	ILMN_2699509	ILMN_218730	-4.04	0.0000
Ptprs	ILMN_2737758	ILMN_212655	-4.04	0.0001
Ap2b1	ILMN_1252951	ILMN_211225	-4.04	0.0000
Hic2	ILMN_2677332	ILMN_254142	-4.04	0.0000
Atg2a	ILMN_2963432	ILMN_258683	-4.04	0.0000
Gabrb3	ILMN_3044905	ILMN_240761	-4.05	0.0000
Atp2a3	ILMN_2688236	ILMN_213534	-4.06	0.0000
1110013L07Rik	ILMN_1217406	ILMN_227473	-4.07	0.0000
LOC100046775	ILMN_1225654	ILMN_313813	-4.08	0.0001
2610109H07Rik	ILMN_3089931	ILMN_236248	-4.08	0.0000
Ascl3	ILMN_2691996	ILMN_218130	-4.09	0.0001
Paox	ILMN_1220022	ILMN_212962	-4.09	0.0001
Dpp9	ILMN_2964344	ILMN_220414	-4.09	0.0000
Esrrg	ILMN_1245243	ILMN_231171	-4.10	0.0006
Zfp259	ILMN_1241970	ILMN_185497	-4.12	0.0000
Prokr1	ILMN_2612018	ILMN_208651	-4.12	0.0002
Mfn2	ILMN_2641278	ILMN_213835	-4.12	0.0000
F630003A18Rik	ILMN_2831116	ILMN_221558	-4.12	0.0011
Rbm12	ILMN_2626300	ILMN_212426	-4.13	0.0000
Cdyl	ILMN_2680574	ILMN_245690	-4.13	0.0036
Slit2	ILMN_1253797	ILMN_259247	-4.14	0.0074
Oaf	ILMN_2895584	ILMN_222394	-4.15	0.0000
Mfng	ILMN_2687661	ILMN_212859	-4.16	0.0000
Cdkl2	ILMN_2657791	ILMN_215298	-4.16	0.0000
Bglap2	ILMN_2944508	ILMN_226483	-4.18	0.0000
Itga5	ILMN_2849449	ILMN_216359	-4.20	0.0000
Fabp5	ILMN_1235908	ILMN_216304	-4.20	0.0000
Olfm1	ILMN_3091574	ILMN_228694	-4.21	0.0000
Ccdc59	ILMN_2877436	ILMN_212729	-4.21	0.0009
Ptgis	ILMN_2724942	ILMN_220660	-4.22	0.0000
Dync1h1	ILMN_2689274	ILMN_217915	-4.22	0.0000
Ppp1r3f	ILMN_1255926	ILMN_253828	-4.23	0.0002
A130022J15Rik	ILMN_2650183	ILMN_214637	-4.23	0.0000
Arfip2	ILMN_1233495	ILMN_211782	-4.24	0.0017
Asgr1	ILMN_2608703	ILMN_210744	-4.25	0.0000
Clcn1	ILMN_2802672	ILMN_218275	-4.25	0.0001
Olfr1413	ILMN_2757778	ILMN_222991	-4.27	0.0000
Zfp169	ILMN_1244864	ILMN_222842	-4.27	0.0000
Wdr53	ILMN_2706964	ILMN_224672	-4.29	0.0000
Zmat3	ILMN_2418426	ILMN_184413	-4.30	0.0001
Jak2	ILMN_1245579	ILMN_214833	-4.31	0.0000
Gmcl1	ILMN_3009951	ILMN_214119	-4.31	0.0001
Apex2	ILMN_1240188	ILMN_193546	-4.32	0.0000
Tcfap2b	ILMN_3151298	ILMN_238889	-4.32	0.0002
Cno	ILMN_2591411	ILMN_208978	-4.33	0.0000
Paf1	ILMN_2744968	ILMN_220830	-4.34	0.0008
Muc13	ILMN_2717678	ILMN_217318	-4.34	0.0019
Cldn4	ILMN_1223949	ILMN_209388	-4.35	0.0000
Bcl2l10	ILMN_1215546	ILMN_218595	-4.36	0.0031
Rps6ka4	ILMN_2740764	ILMN_221814	-4.36	0.0001

AI597479	ILMN_2970241	ILMN_215743	-4.37	0.0000
Gps1	ILMN_1254788	ILMN_209986	-4.38	0.0000
Ccdc68	ILMN_2929572	ILMN_223560	-4.41	0.0005
Abcc6	ILMN_2934941	ILMN_209934	-4.42	0.0010
Atp2a3	ILMN_2900462	ILMN_213534	-4.42	0.0000
P2ry2	ILMN_2872106	ILMN_217971	-4.43	0.0000
Olfr113	ILMN_2759037	ILMN_223076	-4.44	0.0000
BC011426	ILMN_1214025	ILMN_220716	-4.45	0.0000
Nfam1	ILMN_1248347	ILMN_218492	-4.46	0.0000
Kcnab1	ILMN_1216469	ILMN_211650	-4.48	0.0000
Preb	ILMN_2683621	ILMN_234820	-4.49	0.0000
ENSMUSG00000073257	ILMN_3083227	ILMN_229047	-4.49	0.0035
Chrna9	ILMN_2717580	ILMN_220096	-4.50	0.0000
Klra16	ILMN_3005175	ILMN_196756	-4.50	0.0000
Tapbpl	ILMN_2606498	ILMN_196775	-4.50	0.0000
Bglap-rs1	ILMN_1233122	ILMN_236469	-4.51	0.0035
Olfr606	ILMN_2741953	ILMN_233001	-4.53	0.0000
Stac	ILMN_1245844	ILMN_220646	-4.54	0.0000
Arglu1	ILMN_2594128	ILMN_256792	-4.55	0.0000
Sync	ILMN_2982123	ILMN_217966	-4.55	0.0000
Wnk2	ILMN_1214402	ILMN_225853	-4.55	0.0000
Madcam1	ILMN_2865448	ILMN_220787	-4.56	0.0000
Foxi2	ILMN_2894125	ILMN_219115	-4.56	0.0000
Arrdc3	ILMN_2666279	ILMN_216006	-4.57	0.0000
D17H6S56E-3	ILMN_2889044	ILMN_223316	-4.58	0.0005
Hist1h2ao	ILMN_2836654	ILMN_196731	-4.62	0.0000
Npal2	ILMN_2723369	ILMN_235586	-4.65	0.0046
Mup4	ILMN_2592166	ILMN_209061	-4.66	0.0000
Rgs1	ILMN_2897891	ILMN_226813	-4.67	0.0000
Usmg5	ILMN_1219002	ILMN_194875	-4.67	0.0000
Cish	ILMN_2718330	ILMN_220158	-4.69	0.0000
Arc	ILMN_2597827	ILMN_209647	-4.69	0.0009
Dcx	ILMN_2931623	ILMN_214635	-4.71	0.0014
Ank2	ILMN_3158444	ILMN_235890	-4.72	0.0000
Olfr1491	ILMN_1256579	ILMN_223138	-4.74	0.0000
BC049635	ILMN_3125842	ILMN_211809	-4.75	0.0004
2310008H04Rik	ILMN_2964816	ILMN_244230	-4.75	0.0000
Ptpdc1	ILMN_2805624	ILMN_244829	-4.75	0.0000
Tceb1	ILMN_2995698	ILMN_208836	-4.76	0.0000
Olfr628	ILMN_2593666	ILMN_209214	-4.79	0.0000
Bid	ILMN_2757287	ILMN_208644	-4.79	0.0000
4932414N04Rik	ILMN_2888872	ILMN_240286	-4.80	0.0000
Vps33b	ILMN_2864844	ILMN_184886	-4.82	0.0002
Anks6	ILMN_2612904	ILMN_240901	-4.82	0.0013
Slc44a2	ILMN_1232195	ILMN_209839	-4.82	0.0000
Ttc3	ILMN_2841721	ILMN_187235	-4.82	0.0000
Tns4	ILMN_1236029	ILMN_216838	-4.83	0.0000
5330439J01Rik	ILMN_2772728	ILMN_224379	-4.84	0.0000
9130005N14Rik	ILMN_1217235	ILMN_211732	-4.85	0.0000
Ulk2	ILMN_1244059	ILMN_186492	-4.86	0.0000

Dnase1	ILMN_2734251	ILMN_214587	-4.87	0.0000
Cdc5l	ILMN_2754425	ILMN_216571	-4.88	0.0000
Zmym2	ILMN_1245540	ILMN_185418	-4.90	0.0000
Snx13	ILMN_2877227	ILMN_261707	-4.91	0.0000
Iars	ILMN_2810045	ILMN_212043	-4.92	0.0000
Phtf1	ILMN_2939522	ILMN_231517	-4.93	0.0002
Dlat	ILMN_2757993	ILMN_223004	-4.94	0.0000
Sidt1	ILMN_1248211	ILMN_256339	-4.95	0.0000
Rcan2	ILMN_3033007	ILMN_220888	-4.97	0.0000
Pcnt	ILMN_2721221	ILMN_220374	-4.97	0.0000
Shh	ILMN_2802979	ILMN_260687	-4.99	0.0000
Prrx1	ILMN_3057018	ILMN_252720	-5.01	0.0000
Cachd1	ILMN_1241872	ILMN_218385	-5.02	0.0004
EG574403	ILMN_3162060	ILMN_256035	-5.03	0.0019
Tgif1	ILMN_1253854	ILMN_211263	-5.05	0.0000
Car15	ILMN_2871660	ILMN_217620	-5.10	0.0000
Dmrtc1b	ILMN_2929990	ILMN_234785	-5.11	0.0000
5830417C01Rik	ILMN_2741737	ILMN_212594	-5.12	0.0000
Phf20l1	ILMN_1254531	ILMN_253113	-5.12	0.0001
Ccrk	ILMN_2627072	ILMN_212415	-5.15	0.0000
Pparbp	ILMN_2658227	ILMN_215333	-5.16	0.0000
Calm3	ILMN_1215681	ILMN_219075	-5.16	0.0000
Svop	ILMN_2765454	ILMN_227659	-5.19	0.0004
Cdkl1	ILMN_2852737	ILMN_210720	-5.20	0.0000
Serpinb8	ILMN_2829250	ILMN_214122	-5.21	0.0000
Rab22a	ILMN_1214708	ILMN_225250	-5.21	0.0000
Tbx3	ILMN_3139514	ILMN_212973	-5.26	0.0000
Dpf3	ILMN_2853983	ILMN_211654	-5.27	0.0000
Erh	ILMN_2919786	ILMN_212781	-5.29	0.0000
Kcna10	ILMN_1240294	ILMN_261328	-5.33	0.0000
Med7	ILMN_1225666	ILMN_223180	-5.34	0.0002
Sertad4	ILMN_2918656	ILMN_220872	-5.38	0.0000
LOC100046039	ILMN_2733514	ILMN_319133	-5.38	0.0000
Ythdf2	ILMN_2868374	ILMN_239002	-5.41	0.0000
Ifi202b	ILMN_3014753	ILMN_210704	-5.48	0.0000
Copg	ILMN_3074952	ILMN_236440	-5.49	0.0000
Cand2	ILMN_2619869	ILMN_211826	-5.50	0.0000
Tmem44	ILMN_2661495	ILMN_259693	-5.53	0.0000
Herc2	ILMN_2717727	ILMN_220107	-5.61	0.0000
Abcb4	ILMN_2648742	ILMN_214510	-5.61	0.0000
4121402D02Rik	ILMN_2746421	ILMN_210984	-5.64	0.0002
Snapin	ILMN_2963906	ILMN_209347	-5.69	0.0000
Mic2l1	ILMN_2663576	ILMN_215787	-5.73	0.0000
Pcdh7	ILMN_2881857	ILMN_226390	-5.82	0.0000
Alg3	ILMN_2744459	ILMN_211821	-5.82	0.0000
Kifap3	ILMN_2654532	ILMN_215014	-5.83	0.0000
Chst7	ILMN_1216374	ILMN_211189	-5.85	0.0000
Ngfb	ILMN_2660233	ILMN_215493	-5.90	0.0000
Abpg	ILMN_2696136	ILMN_218461	-5.97	0.0000
Olfml2a	ILMN_1232604	ILMN_234057	-5.97	0.0000

Car6	ILMN_1257323	ILMN_258206	-6.05	0.0000
Klk1b5	ILMN_1224893	ILMN_196768	-6.07	0.0000
OTTMUSG00000007485	ILMN_2881950	ILMN_241785	-6.07	0.0000
Grhl1	ILMN_2727118	ILMN_214278	-6.07	0.0000
Mup5	ILMN_3079257	ILMN_214274	-6.28	0.0000
Spt1	ILMN_2845370	ILMN_217293	-6.29	0.0000
Sema6c	ILMN_2876749	ILMN_234745	-6.36	0.0000
Klk1b5	ILMN_2731191	ILMN_196768	-6.46	0.0000
Klk1b21	ILMN_2651099	ILMN_196711	-6.48	0.0000
Vps53	ILMN_1258453	ILMN_213797	-6.65	0.0000
Clec4b1	ILMN_2959372	ILMN_196321	-6.66	0.0002
Klk1b27	ILMN_1252131	ILMN_196774	-6.69	0.0000
Ngfb	ILMN_2937596	ILMN_215493	-6.70	0.0000
Klk1b11	ILMN_2632912	ILMN_196710	-6.74	0.0000
Rapsn	ILMN_2967654	ILMN_211548	-6.75	0.0000
Klk1b4	ILMN_1238736	ILMN_199361	-6.77	0.0000
Muc10	ILMN_2623234	ILMN_212146	-6.79	0.0000
Vps36	ILMN_1240149	ILMN_252294	-6.79	0.0000
Klk1b21	ILMN_2732087	ILMN_196711	-6.81	0.0000
Klk1b8	ILMN_1216962	ILMN_212623	-6.92	0.0000
Muc10	ILMN_1247393	ILMN_212146	-6.96	0.0000
Pip	ILMN_2621766	ILMN_212014	-7.02	0.0000
Klk1b9	ILMN_2784773	ILMN_196709	-7.05	0.0000
H1fx	ILMN_1247646	ILMN_221878	-7.08	0.0000
Snip1	ILMN_2974480	ILMN_219094	-7.38	0.0000
Klk1b16	ILMN_1259613	ILMN_196767	-7.40	0.0000
Klk1b9	ILMN_2723594	ILMN_196709	-7.55	0.0000
2610019A05Rik	ILMN_2731592	ILMN_221150	-7.64	0.0000
Swap70	ILMN_2752989	ILMN_220815	-7.70	0.0000
Klk1b27	ILMN_3009447	ILMN_196774	-7.74	0.0000
LOC100044256	ILMN_2703052	ILMN_313374	-7.82	0.0000
Abpa	ILMN_1249191	ILMN_216701	-7.83	0.0000
Lman1l	ILMN_3127595	ILMN_214284	-7.84	0.0000
Abpb	ILMN_2915893	ILMN_250100	-7.87	0.0000
Narfl	ILMN_1232240	ILMN_215503	-7.97	0.0000
Smgc	ILMN_2775962	ILMN_244638	-7.99	0.0000
Klk1b24	ILMN_2622463	ILMN_196770	-8.02	0.0000
Klk1b11	ILMN_2979432	ILMN_196710	-8.07	0.0000
Egfbp2	ILMN_2791241	ILMN_196757	-8.12	0.0000
Abpb	ILMN_2771109	ILMN_315214	-8.35	0.0000
Klk1b26	ILMN_1217308	ILMN_196712	-8.36	0.0000
Smgc	ILMN_2620853	ILMN_210125	-8.59	0.0000
Rev3l	ILMN_1236221	ILMN_219245	-8.59	0.0000
Klk1b22	ILMN_2946653	ILMN_239601	-8.91	0.0000
Smgc	ILMN_2602496	ILMN_210125	-9.17	0.0000
Klk1b4	ILMN_2697256	ILMN_199361	-9.20	0.0000
Dst	ILMN_1251692	ILMN_211736	-9.57	0.0000
Abpg	ILMN_2599449	ILMN_209813	-9.78	0.0000
Klk1	ILMN_2760199	ILMN_196747	-10.49	0.0000
Fkbp11	ILMN_1224635	ILMN_210599	-14.00	0.0000
