# Supplementary material for: Deconvolution of expression microarray data reveals 131I-induced responses otherwise undetected in thyroid tissue
Source: PLoS One. 2018 Jul 12;13(7):e0197911. doi: 10.1371/journal.pone.0197911 (PMC6042689; doi:10.1371/journal.pone.0197911)
Supplement: S4 Table — (PDF) [file pone.0197911.s006.pdf]

Supplemental Table 4. List of significantly regulated transcripts in deconvolved data for C-cells

The log2-ratio threshold was set to 0.58 with a p-value threshold of 0.01.
Multiple test correction for FDR was performed using the Benjamini Hochberg method in NEXUS 3.0 (BioDiscovery; El Segundo, CA, USA).
The pool size for intensity based pooling was set to 200.

Gene Symbol	Probe	Transcript	Log_2 ratio	adjusted p-value
Myoz2	ILMN_1234857	ILMN_223119	14.27	0.0000
Tnnt3	ILMN_2469018	ILMN_190311	11.50	0.0000
Atp2a1	ILMN_2666864	ILMN_216061	10.55	0.0000
Tnnc2	ILMN_2882658	ILMN_188936	10.34	0.0000
Dmwd	ILMN_2711355	ILMN_214760	10.10	0.0000
Tpm2	ILMN_2482209	ILMN_191790	9.73	0.0000
LOC100046608	ILMN_2706514	ILMN_311207	9.71	0.0000
Bzw2	ILMN_2644587	ILMN_214132	9.65	0.0000
Pvalb	ILMN_1218223	ILMN_213948	9.54	0.0000
Vgll2	ILMN_2909248	ILMN_185749	9.28	0.0000
Rai2	ILMN_1223520	ILMN_214074	9.18	0.0000
Mybpc2	ILMN_2676012	ILMN_242030	9.04	0.0000
Myom1	ILMN_2626442	ILMN_241505	8.96	0.0000
Mlf1	ILMN_3112219	ILMN_246637	8.96	0.0000
Nrap	ILMN_3061287	ILMN_247151	8.91	0.0000
Myh2	ILMN_1219423	ILMN_219104	8.82	0.0000
Eef1a2	ILMN_2666990	ILMN_214177	8.70	0.0000
Pgam2	ILMN_2588815	ILMN_208713	8.67	0.0000
Ptp4a3	ILMN_2655260	ILMN_213418	8.64	0.0000
Myh4	ILMN_1241214	ILMN_229231	8.61	0.0000
Myh1	ILMN_2865744	ILMN_239496	8.43	0.0000
H19	ILMN_2906728	ILMN_219881	8.34	0.0000
Gpc1	ILMN_2635784	ILMN_244157	8.32	0.0000
Zadh1	ILMN_2476885	ILMN_191185	8.17	0.0000
Ckmt2	ILMN_2698052	ILMN_218610	8.10	0.0000
Nrap	ILMN_1235070	ILMN_217102	8.00	0.0000
Eef1a2	ILMN_2971559	ILMN_214177	7.99	0.0000
Gpr63	ILMN_2750800	ILMN_222518	7.99	0.0000
5730593F17Rik	ILMN_1224162	ILMN_223419	7.96	0.0000
Actn2	ILMN_2797061	ILMN_223487	7.89	0.0000
Arpp21	ILMN_1241866	ILMN_217469	7.89	0.0000
Dbx1	ILMN_2963413	ILMN_240000	7.84	0.0029
Yipf7	ILMN_1220086	ILMN_216898	7.75	0.0000
Cbl	ILMN_2649011	ILMN_214537	7.75	0.0000
Tmem120b	ILMN_2909283	ILMN_251445	7.68	0.0002
Tmem157	ILMN_2742739	ILMN_221964	7.60	0.0000
Mpv17	ILMN_3003242	ILMN_199373	7.59	0.0000
Sfxn1	ILMN_2675569	ILMN_216803	7.58	0.0000
Psmd4	ILMN_1228630	ILMN_218193	7.52	0.0000
V1re7	ILMN_1255339	ILMN_187541	7.50	0.0000
Scn4b	ILMN_2813547	ILMN_255776	7.48	0.0000
Pdlim5	ILMN_3137570	ILMN_235674	7.47	0.0000

2310039E09Rik	ILMN_2897214	ILMN_211197	7.41	0.0000
Ntn2l	ILMN_1220826	ILMN_210119	7.35	0.0000
Actn3	ILMN_3008110	ILMN_219398	7.34	0.0000
Csrp3	ILMN_2789650	ILMN_217987	7.34	0.0000
Kcnj12	ILMN_2695900	ILMN_218442	7.20	0.0000
Ppp1r14c	ILMN_2632509	ILMN_261317	7.16	0.0000
Csrp3	ILMN_2789651	ILMN_217987	7.14	0.0002
Olfr1120	ILMN_2654348	ILMN_214999	7.13	0.0003
Scgb3a2	ILMN_2835481	ILMN_222722	7.08	0.0000
Atad1	ILMN_2639230	ILMN_213641	7.07	0.0000
Tkt	ILMN_2780323	ILMN_210657	7.04	0.0000
Actn3	ILMN_2747543	ILMN_219398	7.03	0.0000
Csrp3	ILMN_2742068	ILMN_217987	6.97	0.0000
Gclm	ILMN_1256354	ILMN_219340	6.95	0.0000
Txlnb	ILMN_2886610	ILMN_211914	6.93	0.0008
Olfr173	ILMN_2986948	ILMN_243070	6.91	0.0003
Cd248	ILMN_2896843	ILMN_216345	6.88	0.0000
Tmod4	ILMN_2438793	ILMN_228148	6.81	0.0008
Svep1	ILMN_2691951	ILMN_218126	6.80	0.0000
Ryr1	ILMN_2730425	ILMN_226638	6.71	0.0001
Pknox2	ILMN_3135037	ILMN_254436	6.70	0.0000
Gclc	ILMN_2608016	ILMN_210671	6.70	0.0014
Alpk3	ILMN_3001417	ILMN_209698	6.67	0.0000
Ryr1	ILMN_2733073	ILMN_226638	6.65	0.0000
Lynx1	ILMN_1256369	ILMN_218793	6.60	0.0000
LOC100045343	ILMN_2674281	ILMN_331554	6.58	0.0014
Smpx	ILMN_2785679	ILMN_208970	6.58	0.0040
Nrap	ILMN_3138732	ILMN_247151	6.57	0.0005
Actn3	ILMN_2708303	ILMN_219398	6.55	0.0001
Ryr1	ILMN_2842357	ILMN_226638	6.53	0.0007
Dtna	ILMN_1221805	ILMN_223037	6.50	0.0000
2310038H17Rik	ILMN_2705570	ILMN_219198	6.50	0.0000
Abra	ILMN_2732401	ILMN_256787	6.46	0.0007
Gm216	ILMN_3038912	ILMN_239455	6.44	0.0000
Ruvbl1	ILMN_1228999	ILMN_216028	6.41	0.0000
Trim72	ILMN_2900484	ILMN_252980	6.40	0.0000
Neurl	ILMN_2625940	ILMN_212397	6.39	0.0002
9930111J21Rik	ILMN_2685088	ILMN_217224	6.38	0.0000
Olfr725	ILMN_2814107	ILMN_201572	6.37	0.0046
Kirrel2	ILMN_2629246	ILMN_210693	6.36	0.0001
Ndufs4	ILMN_1234521	ILMN_212301	6.35	0.0000
Myot	ILMN_2444217	ILMN_250811	6.32	0.0000
Tshz3	ILMN_1253593	ILMN_194436	6.30	0.0008
Hspa1l	ILMN_2654682	ILMN_215026	6.28	0.0000
Ywhae	ILMN_1220100	ILMN_195181	6.27	0.0002
Lysmd2	ILMN_1239808	ILMN_222488	6.27	0.0000
Mef2c	ILMN_1214950	ILMN_235198	6.23	0.0000
Ovca2	ILMN_2724815	ILMN_220648	6.22	0.0000
Fbxo32	ILMN_2873444	ILMN_219973	6.16	0.0000
LOC100046163	ILMN_2672325	ILMN_319247	6.15	0.0000

Col3a1	ILMN_2683958	ILMN_211697	6.14	0.0000
Olfr765	ILMN_2911183	ILMN_201568	6.10	0.0000
Ampd1	ILMN_2971142	ILMN_259430	6.06	0.0000
Sgca	ILMN_2938154	ILMN_211934	6.06	0.0000
Mobkl1a	ILMN_2895536	ILMN_229357	6.02	0.0002
Cbfb	ILMN_2671644	ILMN_216489	6.00	0.0001
Schip1	ILMN_1244514	ILMN_216237	5.98	0.0000
Pitx2	ILMN_3118071	ILMN_237162	5.97	0.0087
Tmod1	ILMN_2977404	ILMN_189256	5.96	0.0059
A2bp1	ILMN_1238309	ILMN_216899	5.88	0.0005
Mtrr	ILMN_2742268	ILMN_220536	5.83	0.0000
Gbl	ILMN_2906473	ILMN_213816	5.81	0.0000
Cfl2	ILMN_1241871	ILMN_217503	5.76	0.0000
Acbd3	ILMN_1235783	ILMN_211268	5.75	0.0000
Tm7sf2	ILMN_1241333	ILMN_226159	5.74	0.0007
Slc8a2	ILMN_2715429	ILMN_219936	5.73	0.0005
Pfkm	ILMN_3004553	ILMN_208909	5.72	0.0001
Pfkp	ILMN_2673233	ILMN_226308	5.71	0.0000
2310002L09Rik	ILMN_1214119	ILMN_215549	5.70	0.0000
2310005N03Rik	ILMN_2693258	ILMN_218232	5.69	0.0038
LOC100046690	ILMN_1236869	ILMN_312512	5.69	0.0000
BC061212	ILMN_3098894	ILMN_238862	5.65	0.0019
2010107G23Rik	ILMN_2803564	ILMN_218820	5.65	0.0000
Gpd2	ILMN_1215797	ILMN_214592	5.62	0.0001
Nrp1	ILMN_2669912	ILMN_214296	5.60	0.0036
S3-12	ILMN_2588249	ILMN_208656	5.59	0.0000
1810027O10Rik	ILMN_2875089	ILMN_261137	5.58	0.0000
Npcd	ILMN_3147433	ILMN_247952	5.58	0.0018
Mlf1	ILMN_2658425	ILMN_215347	5.58	0.0000
Slc25a3	ILMN_2751046	ILMN_222536	5.57	0.0004
Dysfip1	ILMN_2613038	ILMN_238591	5.55	0.0000
Opn1mw	ILMN_2706948	ILMN_219296	5.53	0.0000
Wfdc1	ILMN_2466164	ILMN_189984	5.53	0.0001
Loxl1	ILMN_1255871	ILMN_245559	5.51	0.0000
Olfr168	ILMN_1241782	ILMN_214436	5.47	0.0061
Slc41a3	ILMN_2647533	ILMN_250318	5.47	0.0000
Dlk1	ILMN_1244618	ILMN_257636	5.46	0.0003
Tpm2	ILMN_2787172	ILMN_191790	5.45	0.0000
Pdha1	ILMN_2679851	ILMN_217171	5.44	0.0000
Dmn	ILMN_2636266	ILMN_250370	5.43	0.0012
Tmem38a	ILMN_1245673	ILMN_212903	5.43	0.0087
Heatr3	ILMN_3007728	ILMN_208905	5.43	0.0001
Igsf1	ILMN_2660505	ILMN_215516	5.43	0.0000
Cpsf6	ILMN_3042324	ILMN_246878	5.42	0.0005
Mybbp1a	ILMN_1255110	ILMN_228300	5.41	0.0021
Fsd2	ILMN_1242790	ILMN_211390	5.41	0.0000
Zfp617	ILMN_2503418	ILMN_190309	5.38	0.0000
Ndufa13	ILMN_2649937	ILMN_233195	5.37	0.0000
Chek1	ILMN_3009652	ILMN_212847	5.36	0.0000
Myom1	ILMN_2815138	ILMN_241505	5.34	0.0000

Cxcr5	ILMN_2990502	ILMN_214380	5.33	0.0003
Sdc2	ILMN_2619983	ILMN_211837	5.30	0.0000
Olfr1289	ILMN_2866545	ILMN_222169	5.28	0.0015
Trim63	ILMN_3161060	ILMN_238506	5.28	0.0000
Usp13	ILMN_2422325	ILMN_237434	5.27	0.0021
2010305A19Rik	ILMN_2869524	ILMN_251057	5.27	0.0000
Mex3a	ILMN_3072957	ILMN_249418	5.24	0.0000
Phka1	ILMN_1230564	ILMN_219720	5.23	0.0092
Myl2	ILMN_1260428	ILMN_234915	5.20	0.0000
Lce1i	ILMN_1220831	ILMN_214313	5.19	0.0021
Rdh20	ILMN_2598510	ILMN_209717	5.15	0.0003
Ndufab1	ILMN_2732281	ILMN_236862	5.15	0.0074
St3gal2	ILMN_1256849	ILMN_223112	5.14	0.0000
Smad3	ILMN_2938893	ILMN_227724	5.13	0.0002
Pxn	ILMN_1225102	ILMN_232736	5.13	0.0000
Fgf3	ILMN_1231254	ILMN_221278	5.13	0.0024
4931440B09Rik	ILMN_2650180	ILMN_231392	5.11	0.0007
Fdps	ILMN_1225730	ILMN_260757	5.09	0.0024
LOC100038984	ILMN_2606189	ILMN_311308	5.08	0.0024
D12Ertd551e	ILMN_1240131	ILMN_211409	4.99	0.0010
Letm1	ILMN_1248887	ILMN_211496	4.97	0.0000
Zscan2	ILMN_2832322	ILMN_191669	4.96	0.0000
Ppp2r5d	ILMN_2596907	ILMN_209554	4.95	0.0000
Mospd1	ILMN_3006931	ILMN_220553	4.95	0.0000
Dppa1	ILMN_2656079	ILMN_215149	4.93	0.0016
Ss18l1	ILMN_2991263	ILMN_220742	4.90	0.0004
Chrna1	ILMN_1245221	ILMN_209616	4.90	0.0000
Pfkm	ILMN_1252214	ILMN_208909	4.89	0.0003
Lrrc57	ILMN_2898944	ILMN_215482	4.88	0.0000
Zscan12	ILMN_1246450	ILMN_187306	4.87	0.0000
LOC545056	ILMN_2944843	ILMN_241992	4.86	0.0000
Nr4a3	ILMN_1257794	ILMN_216068	4.86	0.0025
Tppp3	ILMN_2655929	ILMN_215136	4.86	0.0001
Dclre1b	ILMN_2638404	ILMN_224631	4.83	0.0026
Igfbp5	ILMN_2964324	ILMN_238078	4.82	0.0037
Fbxl14	ILMN_2972232	ILMN_227130	4.82	0.0000
Cacna2d1	ILMN_2752030	ILMN_222592	4.78	0.0000
Tpra40	ILMN_2440066	ILMN_186972	4.77	0.0012
Sphk1	ILMN_2732199	ILMN_221190	4.77	0.0022
Dedd	ILMN_2857679	ILMN_214171	4.74	0.0043
Mustn1	ILMN_2658461	ILMN_215350	4.74	0.0000
Scoc	ILMN_1216231	ILMN_251319	4.74	0.0013
Rab28	ILMN_2990864	ILMN_254780	4.73	0.0001
Gbp5	ILMN_1244866	ILMN_233457	4.72	0.0001
Gpr152	ILMN_1240344	ILMN_222973	4.70	0.0008
Pfn2	ILMN_1260064	ILMN_210891	4.68	0.0013
Frat2	ILMN_2824002	ILMN_213946	4.68	0.0004
Itgb1bp2	ILMN_2853166	ILMN_214673	4.67	0.0010
Mb	ILMN_1234662	ILMN_210416	4.65	0.0000
Pkia	ILMN_2674122	ILMN_216692	4.64	0.0000

Mfn1	ILMN_2719296	ILMN_220222	4.64	0.0010
2900010J23Rik	ILMN_2733835	ILMN_221314	4.63	0.0003
D630042F21Rik	ILMN_3118298	ILMN_255520	4.63	0.0000
Pdlim3	ILMN_2885990	ILMN_213954	4.62	0.0000
Ddx19b	ILMN_2688135	ILMN_222048	4.62	0.0000
Akr1c14	ILMN_2921215	ILMN_215131	4.61	0.0007
Trpc5	ILMN_2433320	ILMN_186181	4.59	0.0020
Fhl1	ILMN_3117381	ILMN_245416	4.59	0.0000
Hspb3	ILMN_2748205	ILMN_222338	4.58	0.0032
Tcf1	ILMN_2960005	ILMN_259806	4.58	0.0000
Tmem198	ILMN_3162551	ILMN_218012	4.58	0.0000
Pld2	ILMN_2683094	ILMN_217427	4.54	0.0000
Col11a1	ILMN_1217703	ILMN_210481	4.52	0.0000
Mmp27	ILMN_2982829	ILMN_250290	4.52	0.0021
Olfr141	ILMN_2611205	ILMN_210975	4.49	0.0002
C330027C09Rik	ILMN_2709155	ILMN_214430	4.48	0.0001
Cmya1	ILMN_1231765	ILMN_228097	4.48	0.0000
Dusp13	ILMN_2719386	ILMN_220225	4.47	0.0003
Gsdma2	ILMN_2612219	ILMN_211073	4.47	0.0000
Spib	ILMN_2933168	ILMN_258029	4.45	0.0014
BC016579	ILMN_2681156	ILMN_260155	4.45	0.0000
Dhrs7	ILMN_2747820	ILMN_213586	4.45	0.0001
Stk35	ILMN_3155190	ILMN_220535	4.43	0.0087
A930009M04Rik	ILMN_2734855	ILMN_189339	4.42	0.0000
Cacng1	ILMN_2609549	ILMN_210820	4.41	0.0000
Pde4dip	ILMN_3064283	ILMN_228537	4.40	0.0000
Rpgr	ILMN_2703093	ILMN_219012	4.39	0.0000
Rbj	ILMN_2618831	ILMN_211715	4.39	0.0002
Fhl1	ILMN_2713285	ILMN_240873	4.39	0.0023
Psmd11	ILMN_1214057	ILMN_213316	4.37	0.0000
Rab11a	ILMN_1230413	ILMN_247934	4.36	0.0008
Olfr961	ILMN_2990768	ILMN_217807	4.36	0.0086
Olfr1512	ILMN_2611621	ILMN_211017	4.36	0.0005
EG317677	ILMN_2639513	ILMN_213670	4.35	0.0005
C530044N13Rik	ILMN_1255438	ILMN_242932	4.34	0.0002
As3mt	ILMN_2707198	ILMN_216722	4.33	0.0100
2900026A02Rik	ILMN_1251935	ILMN_243658	4.32	0.0000
Clps	ILMN_1246265	ILMN_220484	4.31	0.0000
Adssl1	ILMN_2711688	ILMN_210075	4.31	0.0005
Eln	ILMN_2697304	ILMN_218560	4.29	0.0000
Tex12	ILMN_2719637	ILMN_220245	4.29	0.0041
Kif1b	ILMN_3034877	ILMN_207470	4.28	0.0001
Lrrn1	ILMN_2759736	ILMN_223132	4.25	0.0005
Hoxc5	ILMN_1230286	ILMN_216543	4.25	0.0000
Dedd	ILMN_2645074	ILMN_214171	4.24	0.0057
Hspb7	ILMN_2677662	ILMN_216975	4.23	0.0002
Asb2	ILMN_2765759	ILMN_223558	4.22	0.0002
Ldlrad2	ILMN_2922672	ILMN_256100	4.22	0.0058
Klhl31	ILMN_1247118	ILMN_216835	4.22	0.0000
Ppp1r3c	ILMN_2667091	ILMN_216078	4.22	0.0021

Mb	ILMN_2954987	ILMN_210416	4.21	0.0039
Serpinh1	ILMN_2822850	ILMN_260096	4.20	0.0000
Cxcl12	ILMN_3078973	ILMN_251788	4.19	0.0005
Tnfaip8l1	ILMN_2780552	ILMN_220689	4.18	0.0015
Adcyap1r1	ILMN_2717037	ILMN_188920	4.18	0.0001
1110057K04Rik	ILMN_2756686	ILMN_213087	4.17	0.0015
Casc4	ILMN_2614203	ILMN_211277	4.17	0.0044
Pdcd6	ILMN_2681832	ILMN_217320	4.17	0.0001
Ttc16	ILMN_2471317	ILMN_190569	4.17	0.0074
Lsr	ILMN_2716185	ILMN_218740	4.17	0.0070
Ptprs	ILMN_2688930	ILMN_212655	4.15	0.0030
Il7r	ILMN_2975500	ILMN_215453	4.15	0.0000
Prrx1	ILMN_3057018	ILMN_252720	4.15	0.0000
BC050196	ILMN_1227083	ILMN_221397	4.14	0.0001
Zfp112	ILMN_1221408	ILMN_185070	4.13	0.0052
Sh3rf2	ILMN_2942551	ILMN_213769	4.13	0.0007
Aqp11	ILMN_1225901	ILMN_216379	4.12	0.0017
Pkd2l1	ILMN_2731331	ILMN_221125	4.11	0.0044
LOC433801	ILMN_3023451	ILMN_260633	4.11	0.0021
4921504E06Rik	ILMN_2691910	ILMN_226880	4.09	0.0000
Spred2	ILMN_2590010	ILMN_208837	4.08	0.0023
2900073G15Rik	ILMN_2682046	ILMN_217335	4.08	0.0006
Tmem92	ILMN_2866742	ILMN_257054	4.07	0.0025
Rcbtb2	ILMN_2812084	ILMN_222802	4.06	0.0048
Glyat	ILMN_2697749	ILMN_232014	4.03	0.0005
Plxnd1	ILMN_3162354	ILMN_260028	4.00	0.0000
Trpm3	ILMN_3025252	ILMN_239137	3.97	0.0008
Rpap1	ILMN_2602151	ILMN_210094	3.97	0.0000
AU021092	ILMN_2825905	ILMN_234071	3.97	0.0001
Cwf19l2	ILMN_2860135	ILMN_213168	3.97	0.0044
Ptp4a2	ILMN_2647282	ILMN_210989	3.95	0.0001
Asb10	ILMN_2919777	ILMN_210145	3.95	0.0017
Stx4a	ILMN_2822053	ILMN_233733	3.93	0.0012
Npy5r	ILMN_3002189	ILMN_237214	3.93	0.0013
Fbxo31	ILMN_1252057	ILMN_217743	3.93	0.0003
Iars	ILMN_1224044	ILMN_212043	3.93	0.0002
EG546150	ILMN_2995474	ILMN_256320	3.92	0.0003
Usp6nl	ILMN_1254995	ILMN_184522	3.92	0.0020
Wars2	ILMN_2888110	ILMN_187788	3.91	0.0095
Adamts19	ILMN_1227352	ILMN_222462	3.90	0.0031
Tpi1	ILMN_2932359	ILMN_186788	3.89	0.0004
Zfp93	ILMN_1232697	ILMN_190304	3.88	0.0001
Tm4sf4	ILMN_2479370	ILMN_191474	3.88	0.0001
Dgke	ILMN_2658108	ILMN_248261	3.87	0.0027
Fbxo32	ILMN_2752994	ILMN_219973	3.87	0.0001
Timm9	ILMN_1217794	ILMN_249971	3.86	0.0000
Atp1b4	ILMN_2768612	ILMN_223752	3.85	0.0019
Tpi1	ILMN_1230137	ILMN_186788	3.85	0.0000
Gm106	ILMN_2802928	ILMN_250562	3.84	0.0004
Pltp	ILMN_2589401	ILMN_208776	3.84	0.0005

Smpx	ILMN_2591308	ILMN_208970	3.82	0.0002
Prps1	ILMN_1230518	ILMN_210269	3.81	0.0024
Gtf2a1	ILMN_1244651	ILMN_211195	3.81	0.0011
Ankrd23	ILMN_2741117	ILMN_221842	3.81	0.0059
Spg7	ILMN_2742420	ILMN_201532	3.81	0.0008
Klhl7	ILMN_2660864	ILMN_215548	3.76	0.0015
Acbd4	ILMN_3071515	ILMN_217938	3.75	0.0002
Ubfd1	ILMN_2777104	ILMN_256119	3.74	0.0025
LOC100044222	ILMN_1223207	ILMN_314340	3.73	0.0004
Fgf12	ILMN_1221164	ILMN_220239	3.73	0.0003
Pex11a	ILMN_1249264	ILMN_251549	3.73	0.0048
Olfr1234	ILMN_1229384	ILMN_214550	3.72	0.0000
Tubb3	ILMN_1252657	ILMN_190058	3.72	0.0010
Hoxa1	ILMN_2603334	ILMN_210214	3.72	0.0002
Gdf9	ILMN_1225090	ILMN_214936	3.72	0.0074
Josd2	ILMN_2667614	ILMN_216123	3.69	0.0044
Rmnd5b	ILMN_2918663	ILMN_217949	3.69	0.0050
Dhcr24	ILMN_2747031	ILMN_222259	3.69	0.0033
Ccnl2	ILMN_2601488	ILMN_254859	3.68	0.0011
Gbl	ILMN_2906471	ILMN_213816	3.67	0.0008
Tmem160	ILMN_2914295	ILMN_225101	3.66	0.0003
Capza1	ILMN_2921526	ILMN_223703	3.65	0.0014
Ube2a	ILMN_2476804	ILMN_191175	3.64	0.0002
Tnnt1	ILMN_2460136	ILMN_189308	3.64	0.0012
Snta1	ILMN_2734142	ILMN_221335	3.64	0.0090
Slc2a5	ILMN_1218769	ILMN_221068	3.64	0.0003
Sqrdl	ILMN_1247947	ILMN_223305	3.64	0.0000
Mtrf1	ILMN_1230693	ILMN_199371	3.64	0.0004
Ube2f	ILMN_2614522	ILMN_251929	3.61	0.0021
A930008G19Rik	ILMN_2710429	ILMN_219556	3.61	0.0068
Uros	ILMN_2452383	ILMN_188403	3.61	0.0057
2900010M23Rik	ILMN_2877367	ILMN_212351	3.61	0.0008
E4f1	ILMN_2650893	ILMN_214697	3.60	0.0011
Prkcdbp	ILMN_2956942	ILMN_214335	3.60	0.0002
Crip3	ILMN_3104529	ILMN_234559	3.60	0.0001
Cyp2c67	ILMN_3074610	ILMN_231858	3.59	0.0002
Ncdn	ILMN_2864497	ILMN_215866	3.59	0.0004
Slc44a2	ILMN_2599719	ILMN_209839	3.59	0.0068
Hoxa13	ILMN_1237317	ILMN_213519	3.57	0.0003
Erg	ILMN_1260248	ILMN_213964	3.56	0.0001
Srpk3	ILMN_2684515	ILMN_217542	3.56	0.0014
Mlxip	ILMN_3139380	ILMN_226707	3.56	0.0065
Nox4	ILMN_2698499	ILMN_218644	3.55	0.0001
Prrx1	ILMN_2678094	ILMN_252720	3.54	0.0076
Kti12	ILMN_2599412	ILMN_209809	3.53	0.0002
Mbip	ILMN_1217143	ILMN_216427	3.53	0.0013
Api5	ILMN_2705276	ILMN_219743	3.52	0.0081
CN716893	ILMN_3039183	ILMN_231406	3.50	0.0027
Gipc1	ILMN_2594419	ILMN_209294	3.50	0.0003
Hmox1	ILMN_2788073	ILMN_220840	3.50	0.0067

Olfr494	ILMN_2782683	ILMN_199408	3.50	0.0055
BC057627	ILMN_2977903	ILMN_260182	3.49	0.0009
Wars2	ILMN_1213053	ILMN_187788	3.48	0.0010
Dtna	ILMN_2724339	ILMN_212398	3.47	0.0006
Adam3	ILMN_2772470	ILMN_240204	3.47	0.0002
Trip12	ILMN_2504698	ILMN_187207	3.46	0.0053
Csnk1a1	ILMN_1254710	ILMN_223144	3.46	0.0053
Mare	ILMN_2717209	ILMN_248390	3.46	0.0023
Ntrk3	ILMN_2632337	ILMN_209768	3.43	0.0020
Cep27	ILMN_2630468	ILMN_212801	3.41	0.0010
Trak1	ILMN_2937122	ILMN_252788	3.41	0.0021
Sprr2f	ILMN_2695944	ILMN_218446	3.40	0.0086
Pogz	ILMN_2857204	ILMN_229041	3.39	0.0010
Nod2	ILMN_2702208	ILMN_249891	3.39	0.0058
Jam2	ILMN_2592881	ILMN_208960	3.38	0.0069
Pqlc2	ILMN_2725548	ILMN_229604	3.38	0.0016
Ergic1	ILMN_2745433	ILMN_222155	3.38	0.0002
Ndrg2	ILMN_3001650	ILMN_244033	3.36	0.0002
Cdyl	ILMN_2647931	ILMN_245690	3.32	0.0029
Acy1l2	ILMN_3160507	ILMN_262070	3.31	0.0027
Ddx52	ILMN_2725504	ILMN_220701	3.27	0.0088
P2ry2	ILMN_2872106	ILMN_217971	3.25	0.0019
Ipo5	ILMN_2764995	ILMN_223513	3.25	0.0083
Clip4	ILMN_2606470	ILMN_249105	3.24	0.0057
Fsd2	ILMN_1215238	ILMN_211390	3.23	0.0038
Fbxo42	ILMN_2781835	ILMN_215247	3.22	0.0072
Narfl	ILMN_1232240	ILMN_215503	3.22	0.0009
Kbtbd2	ILMN_2824593	ILMN_215005	3.21	0.0076
Atp10d	ILMN_1235196	ILMN_210812	3.20	0.0011
Cyp3a44	ILMN_1247769	ILMN_219298	3.19	0.0072
Rab20	ILMN_2764968	ILMN_223510	3.19	0.0007
Baz2a	ILMN_2652385	ILMN_245530	3.18	0.0090
Fbxo40	ILMN_3049851	ILMN_259819	3.16	0.0005
Ift57	ILMN_2646089	ILMN_238013	3.16	0.0080
Sgk3	ILMN_2665364	ILMN_213787	3.10	0.0004
Minpp1	ILMN_2703702	ILMN_210498	3.05	0.0058
Ttc35	ILMN_2814431	ILMN_223548	3.04	0.0023
Lrtm2	ILMN_2735391	ILMN_221423	3.01	0.0014
Ppp2r5d	ILMN_2608394	ILMN_209554	2.98	0.0010
Pwwp2b	ILMN_2833781	ILMN_228600	2.92	0.0078
Olfr638	ILMN_2633907	ILMN_213123	2.91	0.0003
Olfr1093	ILMN_2695437	ILMN_218407	2.86	0.0038
Hnrpd	ILMN_2771260	ILMN_242829	2.83	0.0006
Chia	ILMN_2955940	ILMN_208632	2.83	0.0008
6530421E24Rik	ILMN_2764974	ILMN_223511	2.82	0.0019
Klhdc1	ILMN_2649635	ILMN_214591	2.82	0.0071
Dr1	ILMN_2728290	ILMN_220893	2.82	0.0000
Ntrk2	ILMN_3061460	ILMN_259664	2.80	0.0046
Abpe	ILMN_1214113	ILMN_249395	2.80	0.0063
1810030N24Rik	ILMN_1221983	ILMN_241295	2.77	0.0004

Avpr2	ILMN_2604189	ILMN_210296	2.70	0.0055
Tfpt	ILMN_1222401	ILMN_212710	2.70	0.0080
Spint4	ILMN_1240354	ILMN_223595	2.65	0.0024
Serbp1	ILMN_2931900	ILMN_215043	2.64	0.0094
Efna2	ILMN_2647949	ILMN_214429	2.64	0.0057
Sell	ILMN_3009860	ILMN_214504	2.53	0.0047
Tnfrsf1b	ILMN_2486186	ILMN_189490	2.52	0.0003
Tlr4	ILMN_2690004	ILMN_217521	2.48	0.0038
Bccip	ILMN_2759920	ILMN_220167	2.45	0.0049
Slc12a1	ILMN_2746614	ILMN_213108	2.44	0.0069
Olfr830	ILMN_2715382	ILMN_219932	2.37	0.0025
Olfr1245	ILMN_2608693	ILMN_210742	2.36	0.0094
Muc1	ILMN_1231229	ILMN_208888	2.32	0.0003
Slc39a4	ILMN_1226293	ILMN_223530	-2.28	0.0018
Msln	ILMN_1231379	ILMN_211608	-2.32	0.0074
Plch2	ILMN_2925433	ILMN_217607	-2.49	0.0088
Yod1	ILMN_2595026	ILMN_209354	-2.50	0.0091
Cd74	ILMN_3089584	ILMN_214824	-2.63	0.0045
Gsta2	ILMN_1248849	ILMN_196593	-2.67	0.0038
Ropn1	ILMN_1218144	ILMN_212523	-2.69	0.0012
Olfr161	ILMN_1238823	ILMN_213259	-2.69	0.0045
Mfsd1	ILMN_2718284	ILMN_220153	-2.72	0.0017
Dub1a	ILMN_1224991	ILMN_220220	-2.75	0.0087
Man1a2	ILMN_1241339	ILMN_236517	-2.78	0.0074
C130079G13Rik	ILMN_1232462	ILMN_211646	-2.78	0.0038
Papola	ILMN_2602011	ILMN_240279	-2.80	0.0048
Tcf20	ILMN_2627638	ILMN_201560	-2.81	0.0082
Sesn2	ILMN_1219984	ILMN_218966	-2.81	0.0057
Bcl11b	ILMN_2611022	ILMN_250917	-2.83	0.0081
Art4	ILMN_1219298	ILMN_216680	-2.89	0.0016
Brca2	ILMN_2634750	ILMN_213209	-2.90	0.0035
BC043301	ILMN_3114998	ILMN_250374	-2.90	0.0044
LOC100047009	ILMN_2610309	ILMN_323601	-2.92	0.0053
Fgf14	ILMN_1254854	ILMN_210013	-2.92	0.0088
Mkx	ILMN_1249634	ILMN_219001	-2.93	0.0030
2200001I15Rik	ILMN_2678637	ILMN_217056	-2.98	0.0006
Oas1g	ILMN_2628822	ILMN_212179	-3.00	0.0094
H2-M10.3	ILMN_1247647	ILMN_196776	-3.03	0.0014
Egr3	ILMN_2653543	ILMN_229325	-3.03	0.0088
Fnbp4	ILMN_1260410	ILMN_218544	-3.04	0.0068
Ap1g2	ILMN_1246903	ILMN_220004	-3.05	0.0004
Rtkn	ILMN_3143266	ILMN_233938	-3.05	0.0099
Ubox5	ILMN_2635377	ILMN_213265	-3.05	0.0095
LOC100048187	ILMN_2669015	ILMN_310968	-3.06	0.0023
Slc26a2	ILMN_1224606	ILMN_210168	-3.06	0.0072
Alg5	ILMN_1222503	ILMN_215506	-3.07	0.0023
AK129302	ILMN_3157232	ILMN_240572	-3.08	0.0036
Zfp551	ILMN_3160234	ILMN_245892	-3.08	0.0044
Tpd52	ILMN_3091288	ILMN_246767	-3.08	0.0038
Nfrkb	ILMN_2606777	ILMN_210554	-3.08	0.0043

A630033E08Rik	ILMN_1243037	ILMN_223451	-3.09	0.0084
Mrpl43	ILMN_2598222	ILMN_209684	-3.11	0.0002
Olfr1437	ILMN_2844418	ILMN_227331	-3.11	0.0004
Nudt22	ILMN_1227723	ILMN_218008	-3.12	0.0056
Olfr1309	ILMN_2961541	ILMN_211051	-3.13	0.0041
H3f3b	ILMN_2648292	ILMN_214469	-3.16	0.0038
Rhox9	ILMN_1234077	ILMN_222918	-3.17	0.0004
Snapc3	ILMN_2707634	ILMN_249101	-3.17	0.0023
9130404D08Rik	ILMN_2648607	ILMN_214498	-3.18	0.0042
1700001C19Rik	ILMN_2955312	ILMN_251589	-3.19	0.0019
Casp8	ILMN_3097868	ILMN_236307	-3.20	0.0063
Glce	ILMN_2750543	ILMN_186253	-3.21	0.0000
C130026I21Rik	ILMN_3162925	ILMN_317869	-3.22	0.0006
Sez6l	ILMN_2805839	ILMN_241595	-3.22	0.0004
Pbx2	ILMN_2916489	ILMN_209856	-3.24	0.0027
Ghsr	ILMN_1221883	ILMN_208739	-3.26	0.0002
Prdm6	ILMN_2923625	ILMN_225376	-3.26	0.0030
Slc10a4	ILMN_1251845	ILMN_212856	-3.26	0.0001
Olfr965	ILMN_3161298	ILMN_246582	-3.27	0.0031
Adck5	ILMN_2918317	ILMN_212693	-3.27	0.0034
Cd6	ILMN_3117602	ILMN_209410	-3.28	0.0014
Rpl39	ILMN_2711551	ILMN_219633	-3.29	0.0000
Cuta	ILMN_3041584	ILMN_216302	-3.30	0.0091
Slc10a7	ILMN_3162636	ILMN_234105	-3.30	0.0030
Tac4	ILMN_1237288	ILMN_214963	-3.31	0.0009
Rxfp1	ILMN_2685751	ILMN_240430	-3.31	0.0011
Hbs1l	ILMN_1229378	ILMN_210537	-3.32	0.0011
Polr3h	ILMN_2939022	ILMN_209245	-3.32	0.0064
Diap3	ILMN_2606436	ILMN_238102	-3.33	0.0043
Actn1	ILMN_2844996	ILMN_242046	-3.35	0.0085
D730039F16Rik	ILMN_2912410	ILMN_260134	-3.35	0.0058
Taar2	ILMN_2871830	ILMN_253121	-3.35	0.0003
Rcor1	ILMN_1215969	ILMN_255740	-3.36	0.0005
OTTMUSG00000010136	ILMN_2861524	ILMN_227730	-3.36	0.0008
Cd160	ILMN_2707181	ILMN_219318	-3.38	0.0079
Dapp1	ILMN_2595822	ILMN_209445	-3.41	0.0087
Clk2	ILMN_1238520	ILMN_208789	-3.41	0.0005
Lman1l	ILMN_3051282	ILMN_214284	-3.42	0.0097
Trim23	ILMN_2879221	ILMN_186436	-3.43	0.0087
Fez1	ILMN_1213056	ILMN_214033	-3.44	0.0085
Gm711	ILMN_2641920	ILMN_213893	-3.44	0.0007
Rai1	ILMN_3147230	ILMN_222328	-3.45	0.0067
Igsf4a	ILMN_2505841	ILMN_194372	-3.45	0.0010
Nkain4	ILMN_1242188	ILMN_213011	-3.46	0.0019
Rhbdl1	ILMN_2710802	ILMN_219581	-3.47	0.0019
Fert2	ILMN_2631205	ILMN_245343	-3.47	0.0002
Mup4	ILMN_2592166	ILMN_209061	-3.48	0.0026
Gps1	ILMN_2632714	ILMN_209986	-3.48	0.0062
Numa1	ILMN_2735625	ILMN_221438	-3.49	0.0023
Pgc	ILMN_2606088	ILMN_210484	-3.50	0.0010

Gnl3	ILMN_2601639	ILMN_250637	-3.50	0.0039
Rab25	ILMN_2781721	ILMN_241897	-3.50	0.0004
Lefty2	ILMN_2713164	ILMN_219748	-3.50	0.0043
Pcsk4	ILMN_2860850	ILMN_212743	-3.51	0.0020
Plxnb1	ILMN_1246710	ILMN_211279	-3.53	0.0008
Wfdc6b	ILMN_3062983	ILMN_233050	-3.54	0.0021
Egfl7	ILMN_1215919	ILMN_212285	-3.58	0.0035
Asns	ILMN_2643513	ILMN_213406	-3.59	0.0092
Wdr4	ILMN_2499970	ILMN_190541	-3.62	0.0003
Acot7	ILMN_2660182	ILMN_210493	-3.62	0.0005
9130014G24Rik	ILMN_2931500	ILMN_220828	-3.62	0.0004
Adamts2	ILMN_1226259	ILMN_210818	-3.63	0.0013
Hpcal1	ILMN_2738750	ILMN_221667	-3.64	0.0022
Marveld3	ILMN_2632585	ILMN_213009	-3.64	0.0033
Elf5	ILMN_2938440	ILMN_216298	-3.65	0.0021
Rbbp5	ILMN_2766338	ILMN_230998	-3.66	0.0024
Sfi1	ILMN_1256699	ILMN_220674	-3.67	0.0008
D630044L22Rik	ILMN_2968887	ILMN_242493	-3.67	0.0000
Mrc1	ILMN_1239430	ILMN_210803	-3.67	0.0005
Wwox	ILMN_2480178	ILMN_191565	-3.68	0.0063
Zfp335	ILMN_1243052	ILMN_221830	-3.68	0.0000
Stard10	ILMN_2625601	ILMN_212367	-3.68	0.0026
Ascl3	ILMN_2691996	ILMN_218130	-3.69	0.0003
Zfp498	ILMN_2960521	ILMN_241514	-3.69	0.0000
Lamb3	ILMN_2605512	ILMN_210428	-3.70	0.0002
Pscdbp	ILMN_1251748	ILMN_249986	-3.71	0.0002
Atp6v1e2	ILMN_2755322	ILMN_222823	-3.73	0.0000
Krt6a	ILMN_2738369	ILMN_210419	-3.73	0.0024
V1rc10	ILMN_3024850	ILMN_185781	-3.74	0.0007
Gpx2	ILMN_2674483	ILMN_216720	-3.76	0.0055
Riok3	ILMN_2763548	ILMN_210509	-3.76	0.0001
Gnai2	ILMN_2908855	ILMN_256698	-3.76	0.0007
Oas2	ILMN_2670150	ILMN_216357	-3.77	0.0061
Cd6	ILMN_3042812	ILMN_209410	-3.77	0.0014
Arl6ip6	ILMN_2833965	ILMN_221811	-3.77	0.0097
Chic2	ILMN_1253410	ILMN_223679	-3.78	0.0039
Rqcd1	ILMN_1223145	ILMN_223408	-3.80	0.0002
4930432K21Rik	ILMN_2672745	ILMN_249799	-3.80	0.0025
Rab17	ILMN_2752489	ILMN_215232	-3.81	0.0013
Morn3	ILMN_1223151	ILMN_260560	-3.81	0.0038
Tcf19	ILMN_2624827	ILMN_212294	-3.82	0.0007
Zfp653	ILMN_2755621	ILMN_222843	-3.82	0.0002
LOC100047468	ILMN_1257150	ILMN_328548	-3.85	0.0002
2300002D11Rik	ILMN_3119914	ILMN_260547	-3.85	0.0007
Gm1008	ILMN_3021415	ILMN_238024	-3.85	0.0024
Limk2	ILMN_2636169	ILMN_229871	-3.86	0.0025
Abo	ILMN_2824783	ILMN_216411	-3.86	0.0007
F730014I05Rik	ILMN_2626864	ILMN_212475	-3.89	0.0038
Camkk2	ILMN_1256263	ILMN_217391	-3.91	0.0001
Zfp791	ILMN_2990485	ILMN_234512	-3.92	0.0009

2500002L14Rik	ILMN_2606015	ILMN_210478	-3.92	0.0000
Fkbp11	ILMN_1224635	ILMN_210599	-3.94	0.0001
Cdc42ep4	ILMN_2728431	ILMN_220907	-3.94	0.0002
Dll3	ILMN_2643777	ILMN_214055	-3.95	0.0042
Exosc7	ILMN_2715289	ILMN_243792	-3.96	0.0002
1190003J15Rik	ILMN_2734924	ILMN_238108	-3.97	0.0097
Bsdc1	ILMN_1234100	ILMN_222959	-3.97	0.0004
Arhgef3	ILMN_2645208	ILMN_214179	-3.98	0.0017
Snph	ILMN_1234340	ILMN_208769	-3.98	0.0007
BC037034	ILMN_1257019	ILMN_222839	-3.99	0.0008
Tagln	ILMN_1243652	ILMN_231234	-3.99	0.0007
Blvra	ILMN_1257284	ILMN_215490	-4.00	0.0005
Zic2	ILMN_2470518	ILMN_190478	-4.01	0.0002
Zfp187	ILMN_3145975	ILMN_247449	-4.03	0.0037
2610020H08Rik	ILMN_3143621	ILMN_250105	-4.03	0.0000
Gimap5	ILMN_2643096	ILMN_213996	-4.04	0.0001
Spag16	ILMN_3115326	ILMN_229838	-4.05	0.0000
LOC100044177	ILMN_1232495	ILMN_330013	-4.05	0.0000
Hnrpa3	ILMN_2666458	ILMN_209535	-4.06	0.0000
LOC100045304	ILMN_1237729	ILMN_321302	-4.07	0.0000
Calcr	ILMN_3120532	ILMN_226309	-4.08	0.0002
EG224180	ILMN_2596575	ILMN_322467	-4.08	0.0001
Slfnl1	ILMN_1245376	ILMN_219554	-4.09	0.0075
Cdx4	ILMN_1241829	ILMN_221708	-4.09	0.0037
H2-DMb2	ILMN_2979639	ILMN_244938	-4.10	0.0007
Sv2a	ILMN_2702887	ILMN_218994	-4.10	0.0000
Adam28	ILMN_2664739	ILMN_215881	-4.11	0.0000
Mta3	ILMN_1259783	ILMN_219551	-4.11	0.0001
2210016L21Rik	ILMN_1251123	ILMN_212114	-4.14	0.0001
Htr5b	ILMN_2595319	ILMN_209384	-4.16	0.0002
Olfr1275	ILMN_3161942	ILMN_240876	-4.17	0.0068
Tmem128	ILMN_2674032	ILMN_216686	-4.18	0.0008
Csprs	ILMN_2661289	ILMN_215584	-4.19	0.0000
LOC100043986	ILMN_1228783	ILMN_329081	-4.20	0.0031
Aox1	ILMN_1238033	ILMN_213723	-4.20	0.0000
Slc17a6	ILMN_2922321	ILMN_227493	-4.21	0.0002
Arhgap25	ILMN_3155245	ILMN_211295	-4.23	0.0001
Cyp7b1	ILMN_2601215	ILMN_209999	-4.24	0.0021
Lig1	ILMN_2657356	ILMN_209408	-4.26	0.0005
Ccdc50	ILMN_2737381	ILMN_221575	-4.26	0.0002
Col9a3	ILMN_2597314	ILMN_209595	-4.28	0.0019
Rpa1	ILMN_2750801	ILMN_222519	-4.30	0.0009
Spdef	ILMN_1258162	ILMN_222088	-4.30	0.0005
Gnb1l	ILMN_3059059	ILMN_210607	-4.31	0.0000
Zc3hav1l	ILMN_1215954	ILMN_214101	-4.33	0.0081
Slc25a23	ILMN_2675672	ILMN_259482	-4.35	0.0000
Rnase11	ILMN_2877153	ILMN_252274	-4.36	0.0021
BC050092	ILMN_2694022	ILMN_218292	-4.36	0.0011
Lgals9	ILMN_2752873	ILMN_220025	-4.38	0.0000
Slpi	ILMN_1256817	ILMN_219640	-4.41	0.0000

Pcdhga2	ILMN_2746009	ILMN_222189	-4.42	0.0003
1200014M14Rik	ILMN_2613750	ILMN_211223	-4.43	0.0043
Arid4b	ILMN_3133267	ILMN_222307	-4.45	0.0000
Olfr972	ILMN_2603196	ILMN_210200	-4.48	0.0000
Mlph	ILMN_3162347	ILMN_212716	-4.48	0.0002
Rtp3	ILMN_2864416	ILMN_256934	-4.48	0.0001
Olfr56	ILMN_1255631	ILMN_213568	-4.49	0.0003
Bace2	ILMN_2738449	ILMN_211339	-4.50	0.0013
Cmtm7	ILMN_2648409	ILMN_214480	-4.50	0.0022
Krt7	ILMN_2961152	ILMN_223017	-4.51	0.0003
Cxcl15	ILMN_2635462	ILMN_213274	-4.51	0.0000
Mrpl32	ILMN_2695342	ILMN_229757	-4.53	0.0000
Sf1	ILMN_2449335	ILMN_188043	-4.53	0.0003
Zfp128	ILMN_2828139	ILMN_226457	-4.54	0.0000
Mat2b	ILMN_2588505	ILMN_208679	-4.54	0.0057
Adh1	ILMN_2850077	ILMN_214816	-4.55	0.0000
Epha1	ILMN_2972748	ILMN_213132	-4.55	0.0000
Fem1a	ILMN_2695936	ILMN_259010	-4.58	0.0000
Olfr360	ILMN_1224535	ILMN_221921	-4.60	0.0010
Cdc20	ILMN_2612206	ILMN_211070	-4.60	0.0012
Eftud2	ILMN_2604097	ILMN_210286	-4.60	0.0022
Tns4	ILMN_1236029	ILMN_216838	-4.61	0.0001
Tgfbr1	ILMN_2708965	ILMN_210148	-4.61	0.0000
Usp6nl	ILMN_3079038	ILMN_256050	-4.61	0.0001
Kcnk5	ILMN_2982781	ILMN_218669	-4.62	0.0044
Nfat5	ILMN_2732123	ILMN_221186	-4.62	0.0000
Tmprss2	ILMN_1223880	ILMN_189973	-4.62	0.0027
Tmigd1	ILMN_1220817	ILMN_216690	-4.63	0.0005
Dnajc11	ILMN_1237476	ILMN_237725	-4.63	0.0000
Gpr89	ILMN_2672544	ILMN_216563	-4.69	0.0002
Rcn2	ILMN_2775778	ILMN_245956	-4.69	0.0000
2610528O22Rik	ILMN_2710773	ILMN_184416	-4.69	0.0000
Kcnk1	ILMN_3009501	ILMN_237297	-4.73	0.0036
Gcnt1	ILMN_2672297	ILMN_214513	-4.73	0.0000
Abpg	ILMN_2696136	ILMN_218461	-4.75	0.0000
Angel2	ILMN_3160697	ILMN_235616	-4.76	0.0000
Cd9	ILMN_2725414	ILMN_220691	-4.78	0.0000
Gm813	ILMN_2529961	ILMN_240351	-4.79	0.0000
Il17re	ILMN_3162860	ILMN_258357	-4.79	0.0002
Bet1	ILMN_2833495	ILMN_218670	-4.79	0.0021
Fpgs	ILMN_2870443	ILMN_221380	-4.80	0.0074
Rpl17	ILMN_2677935	ILMN_244238	-4.82	0.0095
Slit2	ILMN_1253797	ILMN_259247	-4.83	0.0008
Tram1	ILMN_2487934	ILMN_192424	-4.83	0.0019
Tcfap2c	ILMN_2783225	ILMN_223577	-4.84	0.0003
Egfr	ILMN_3128725	ILMN_207468	-4.86	0.0095
Hnrpdl	ILMN_2690061	ILMN_217979	-4.91	0.0004
Lman1l	ILMN_3127595	ILMN_214284	-4.92	0.0000
EG668668	ILMN_2890533	ILMN_247763	-4.92	0.0037
4933407N01Rik	ILMN_2604205	ILMN_210297	-4.93	0.0000

Med22	ILMN_3100456	ILMN_252613	-4.93	0.0013
Rabgap1	ILMN_2685079	ILMN_217584	-4.94	0.0030
Fignl1	ILMN_2663604	ILMN_215790	-4.94	0.0048
Npal2	ILMN_2723369	ILMN_235586	-4.95	0.0000
Tcfap2b	ILMN_1223731	ILMN_238889	-4.95	0.0000
Klra4	ILMN_3104704	ILMN_196771	-4.96	0.0016
Use1	ILMN_2667101	ILMN_212247	-4.98	0.0000
Rab27a	ILMN_2614966	ILMN_211354	-5.00	0.0067
Ildr1	ILMN_2857684	ILMN_219794	-5.01	0.0011
Elf5	ILMN_2736380	ILMN_216298	-5.02	0.0000
Pdia4	ILMN_1249670	ILMN_215714	-5.03	0.0000
Myrip	ILMN_2665441	ILMN_215939	-5.06	0.0000
Repin1	ILMN_3131197	ILMN_210453	-5.07	0.0002
Inpp1	ILMN_2901801	ILMN_217833	-5.08	0.0001
Lce1a2	ILMN_2731819	ILMN_221168	-5.09	0.0000
Taar8a	ILMN_1239691	ILMN_238027	-5.10	0.0036
Vamp8	ILMN_1219136	ILMN_190514	-5.10	0.0001
Suv420h1	ILMN_1250037	ILMN_232745	-5.10	0.0018
Mug4	ILMN_2774563	ILMN_317028	-5.11	0.0000
Pip5k1b	ILMN_2711562	ILMN_219634	-5.11	0.0000
Gjb2	ILMN_2999627	ILMN_210347	-5.13	0.0000
Gdf6	ILMN_2663449	ILMN_215775	-5.13	0.0017
Dok1	ILMN_2704141	ILMN_212187	-5.13	0.0023
A530032D15Rik	ILMN_2892518	ILMN_245493	-5.19	0.0000
Odc1	ILMN_3008406	ILMN_219842	-5.20	0.0001
Dyrk3	ILMN_2699522	ILMN_258582	-5.21	0.0000
Prom2	ILMN_1246392	ILMN_214759	-5.23	0.0003
Pabpc1	ILMN_1259482	ILMN_201529	-5.24	0.0004
Kcnn4	ILMN_2765032	ILMN_223516	-5.26	0.0030
Bglap1	ILMN_3101908	ILMN_210884	-5.26	0.0000
Pml	ILMN_2732576	ILMN_249462	-5.26	0.0000
Egf	ILMN_2684104	ILMN_217509	-5.30	0.0000
Pou3f4	ILMN_3162068	ILMN_249620	-5.30	0.0005
Med22	ILMN_3041056	ILMN_225085	-5.31	0.0000
Edem2	ILMN_2669280	ILMN_226722	-5.32	0.0018
Psmb2	ILMN_2949632	ILMN_216114	-5.32	0.0000
Lnx2	ILMN_2598990	ILMN_209763	-5.33	0.0021
Rnasen	ILMN_3144358	ILMN_224762	-5.34	0.0002
Ift20	ILMN_2847618	ILMN_209051	-5.34	0.0005
3110043J09Rik	ILMN_1228093	ILMN_252216	-5.34	0.0000
Sap30l	ILMN_3128784	ILMN_226600	-5.35	0.0019
Olfr1208	ILMN_2630221	ILMN_212778	-5.35	0.0006
Asb3	ILMN_1236376	ILMN_246247	-5.37	0.0062
Lce3f	ILMN_2610594	ILMN_240447	-5.38	0.0000
Rgs7	ILMN_1237219	ILMN_223755	-5.39	0.0002
LOC100046616	ILMN_1259982	ILMN_324507	-5.39	0.0006
Slc12a8	ILMN_2721789	ILMN_312656	-5.40	0.0000
Elf5	ILMN_2669510	ILMN_216298	-5.42	0.0021
Brwd2	ILMN_2476131	ILMN_191104	-5.43	0.0037
Cyp4f16	ILMN_2729452	ILMN_220247	-5.45	0.0000

1810037C20Rik	ILMN_2724415	ILMN_220614	-5.45	0.0002
Ucp3	ILMN_1233132	ILMN_188370	-5.45	0.0002
AI593442	ILMN_2733828	ILMN_215023	-5.47	0.0000
1700027D21Rik	ILMN_1255463	ILMN_222714	-5.53	0.0000
Mup5	ILMN_3158509	ILMN_214274	-5.54	0.0002
Cd44	ILMN_2754990	ILMN_245439	-5.56	0.0000
Oit1	ILMN_2732163	ILMN_221188	-5.57	0.0000
Slc2a2	ILMN_2750284	ILMN_210368	-5.59	0.0000
5031414D18Rik	ILMN_2615575	ILMN_211408	-5.60	0.0003
Efna4	ILMN_1217493	ILMN_217975	-5.62	0.0003
Elf4	ILMN_2672054	ILMN_216519	-5.64	0.0000
Sftpb	ILMN_2733762	ILMN_221309	-5.66	0.0000
Scly	ILMN_2628281	ILMN_212598	-5.68	0.0000
Abcb9	ILMN_2597248	ILMN_184826	-5.68	0.0001
Mycbp2	ILMN_1238676	ILMN_216306	-5.70	0.0029
Cbs	ILMN_1230318	ILMN_216850	-5.70	0.0004
Rhou	ILMN_1237773	ILMN_215476	-5.75	0.0006
Uhmk1	ILMN_2958626	ILMN_231443	-5.75	0.0004
Klk1b1	ILMN_1256119	ILMN_196746	-5.76	0.0071
Drg1	ILMN_1238919	ILMN_214214	-5.81	0.0000
Krt23	ILMN_2671165	ILMN_216445	-5.82	0.0002
Slc7a4	ILMN_2639360	ILMN_229227	-5.83	0.0002
Olfr204	ILMN_3160401	ILMN_249951	-5.84	0.0011
Col12a1	ILMN_2862538	ILMN_212667	-5.85	0.0000
Cdk8	ILMN_2726092	ILMN_211503	-5.85	0.0000
Mif	ILMN_2867835	ILMN_207449	-5.87	0.0000
Rabl5	ILMN_2637398	ILMN_213476	-5.91	0.0013
Foxf1a	ILMN_2667396	ILMN_216104	-5.92	0.0001
Ogt	ILMN_1247078	ILMN_211022	-5.93	0.0081
Slc6a6	ILMN_2641467	ILMN_213853	-5.94	0.0000
Fa2h	ILMN_2746783	ILMN_222241	-5.96	0.0072
D330045A20Rik	ILMN_2706677	ILMN_214422	-5.96	0.0000
Rfxdc1	ILMN_2734616	ILMN_221366	-5.98	0.0002
Zbp1	ILMN_1228653	ILMN_194818	-5.99	0.0000
Mrps11	ILMN_2786708	ILMN_215405	-6.06	0.0036
Stk22a	ILMN_1251399	ILMN_188051	-6.07	0.0062
Flvcr2	ILMN_1248190	ILMN_222509	-6.11	0.0004
Garnl4	ILMN_2940713	ILMN_226588	-6.13	0.0000
Phlda1	ILMN_2754985	ILMN_222798	-6.13	0.0000
9430023L20Rik	ILMN_1238679	ILMN_212911	-6.16	0.0000
Phlda2	ILMN_2425029	ILMN_249746	-6.17	0.0000
Bsnd	ILMN_2669493	ILMN_216297	-6.17	0.0000
Psmd9	ILMN_2594811	ILMN_248163	-6.22	0.0001
Nags	ILMN_2617735	ILMN_211619	-6.23	0.0000
Glra4	ILMN_2663121	ILMN_215745	-6.23	0.0000
Aspn	ILMN_1231476	ILMN_213249	-6.41	0.0000
Tac1	ILMN_1251000	ILMN_220916	-6.42	0.0000
Ccdc38	ILMN_2716995	ILMN_220050	-6.43	0.0000
Cdc2l1	ILMN_1241980	ILMN_213876	-6.43	0.0000
Prrc1	ILMN_2666406	ILMN_216021	-6.56	0.0000

Ercc2	ILMN_1233075	ILMN_212790	-6.62	0.0000
Abpa	ILMN_1249191	ILMN_216701	-6.63	0.0000
Prlr	ILMN_2617005	ILMN_210332	-6.65	0.0003
Acpp	ILMN_2693019	ILMN_218213	-6.70	0.0006
Mkrn1	ILMN_2630018	ILMN_212764	-6.72	0.0000
Erlin2	ILMN_2816315	ILMN_211648	-6.73	0.0000
4933424B01Rik	ILMN_1252267	ILMN_210623	-6.74	0.0000
Eif3h	ILMN_1215703	ILMN_219124	-6.77	0.0000
Smgc	ILMN_2602496	ILMN_210125	-6.79	0.0000
Capg	ILMN_3115281	ILMN_239751	-6.79	0.0000
EG623661	ILMN_2993944	ILMN_227099	-6.87	0.0005
Jmjd1a	ILMN_2707895	ILMN_248615	-6.87	0.0000
Midn	ILMN_1228026	ILMN_209610	-6.89	0.0000
Epn1	ILMN_1250501	ILMN_235298	-6.91	0.0000
Chmp7	ILMN_2843548	ILMN_208690	-6.93	0.0000
Klk1b27	ILMN_1252131	ILMN_196774	-6.99	0.0000
Sec14l1	ILMN_2953751	ILMN_238122	-7.05	0.0000
Edem2	ILMN_2684279	ILMN_226722	-7.05	0.0000
Bsx	ILMN_1228573	ILMN_247817	-7.09	0.0000
Wnt2b	ILMN_2512976	ILMN_195010	-7.09	0.0000
Smgc	ILMN_2775962	ILMN_244638	-7.11	0.0000
Ckmt1	ILMN_2773537	ILMN_235471	-7.15	0.0000
Slc31a1	ILMN_1254437	ILMN_221784	-7.16	0.0000
Rev1	ILMN_2928573	ILMN_213219	-7.22	0.0000
Klk1	ILMN_2760199	ILMN_196747	-7.28	0.0000
Trim23	ILMN_2455830	ILMN_186436	-7.36	0.0000
Rbm35a	ILMN_2947559	ILMN_210806	-7.42	0.0000
Gng7	ILMN_3030009	ILMN_223721	-7.52	0.0000
1500015O10Rik	ILMN_1249000	ILMN_222808	-7.56	0.0000
LOC100041187	ILMN_2589902	ILMN_312862	-7.71	0.0004
LOC100047815	ILMN_1246266	ILMN_310521	-7.76	0.0000
Rps12	ILMN_2956159	ILMN_201571	-7.79	0.0000
Mup5	ILMN_3079257	ILMN_214274	-7.80	0.0000
Muc13	ILMN_2681805	ILMN_217318	-7.82	0.0001
Muc10	ILMN_2623234	ILMN_212146	-7.85	0.0000
Olfr635	ILMN_2704500	ILMN_213553	-7.98	0.0059
Kal1	ILMN_1239717	ILMN_223014	-7.98	0.0000
2810048G17Rik	ILMN_2677612	ILMN_215430	-8.09	0.0000
Igf2bp3	ILMN_1218913	ILMN_218056	-8.11	0.0025
Ngfb	ILMN_2660233	ILMN_215493	-8.32	0.0000
Lama2	ILMN_2769479	ILMN_259802	-8.40	0.0000
Psp	ILMN_1245726	ILMN_216963	-8.41	0.0000
Klk1b4	ILMN_2697256	ILMN_199361	-8.50	0.0000
Klc4	ILMN_1251301	ILMN_238823	-8.54	0.0000
Grhl1	ILMN_1246419	ILMN_214278	-8.68	0.0000
Klk1b5	ILMN_2731191	ILMN_196768	-8.72	0.0000
Lpin2	ILMN_1220680	ILMN_192385	-8.75	0.0000
Tacr3	ILMN_1237995	ILMN_216524	-8.83	0.0000
Klk1b16	ILMN_1259613	ILMN_196767	-8.86	0.0000
Mist1	ILMN_2651388	ILMN_214740	-8.89	0.0000

Ngfb	ILMN_2937596	ILMN_215493	-8.95	0.0000
Wfdc2	ILMN_1236758	ILMN_216447	-8.96	0.0000
Klk1b21	ILMN_2732087	ILMN_196711	-8.96	0.0000
6330503K22Rik	ILMN_1228426	ILMN_210079	-8.97	0.0000
Klk1b24	ILMN_2622463	ILMN_196770	-9.02	0.0000
Klk1b27	ILMN_3009447	ILMN_196774	-9.15	0.0000
Klk1b11	ILMN_2979432	ILMN_196710	-9.23	0.0000
Stk39	ILMN_2751494	ILMN_222561	-9.27	0.0000
Cldn3	ILMN_2634167	ILMN_213147	-9.30	0.0000
Klk1b9	ILMN_2784773	ILMN_196709	-9.34	0.0000
Gm1661	ILMN_2955129	ILMN_241347	-9.68	0.0004
Klk1b9	ILMN_2723594	ILMN_196709	-9.72	0.0000
1700001J03Rik	ILMN_2666574	ILMN_226748	-9.94	0.0000
Rnu6	ILMN_2959272	ILMN_243060	-10.21	0.0000
Muc13	ILMN_2717678	ILMN_217318	-10.24	0.0000
Aqp5	ILMN_3115472	ILMN_217154	-10.68	0.0000
2310057J18Rik	ILMN_2940678	ILMN_210967	-10.69	0.0000
Casq2	ILMN_1247775	ILMN_222933	-10.94	0.0000
Inpp5e	ILMN_2820213	ILMN_214064	-11.50	0.0000
Gsg1	ILMN_1222441	ILMN_252058	-12.07	0.0000
Pigr	ILMN_1225605	ILMN_220227	-13.14	0.0000
Folr1	ILMN_2707541	ILMN_219347	-17.22	0.0000
