# Supplementary material for: Deconvolution of expression microarray data reveals 131I-induced responses otherwise undetected in thyroid tissue
Source: PLoS One. 2018 Jul 12;13(7):e0197911. doi: 10.1371/journal.pone.0197911 (PMC6042689; doi:10.1371/journal.pone.0197911)
Supplement: S5 Table — (PDF) [file pone.0197911.s007.pdf]

Supplemental Table 5. Significantly regulated kallikrein transcripts

Gene symbol	Probe ID	Thyroid tissue (conv.)	Follicular cells (deconv.)	C-cells (deconv.)
		log$_2$ ratio; fold change (adjusted p-value)		
Klk1	ILMN_2760199		-10 ; -1442 (0.0000)	-7.3 ; -156 (0.0000)
Klk1b1	ILMN_1256119		-2.8 ; -7.2 (0.0014)	-5.8 ; -54 (0.0071)
Klk1b11	ILMN_2632912	-5.4 ; -43 (0.0000)	-6.7 ; -107 (0.0000)	
Klk1b11	ILMN_2979432	-6.6 ; -100 (0.0000)	-8.1 ; -268 (0.0000)	-9.2 ; -602 (0.0000)
Klk1b16	ILMN_1259613	-5.7 ; -52 (0.0000)	-7.4 ; -169 (0.0000)	-8.9 ; -463 (0.0000)
Klk1b21	ILMN_2651099	-5.2 ; -38 (0.0000)	-6.5 ; -89 (0.0000)	
Klk1b21	ILMN_2732087		-6.8 ; -112 (0.0000)	-9.0 ; -499 (0.0000)
Klk1b22	ILMN_2946653	-6.6 ; -100 (0.0000)	-8.9 ; -482 (0.0000)	
Klk1b24	ILMN_2622463	-5.8 ; -54 (0.0000)	-8.0 ; -259 (0.0000)	-9.0 ; -520 (0.0000)
Klk1b26	ILMN_1217308	-6.8 ; -115 (0.0000)	-8.4 ; -329 (0.0000)	
Klk1b27	ILMN_1252131	-7.2 ; -150 (0.0000)	-6.7 ; -103 (0.0000)	-7.0 ; -127 (0.0000)
Klk1b27	ILMN_3009447	-6.2 ; -74 (0.0000)	-7.7 ; -214 (0.0000)	-9.1 ; -568 (0.0000)
Klk1b4	ILMN_1238736		-6.8 ; -109 (0.0000)	
Klk1b4	ILMN_2697256	-6.5 ; -91 (0.0000)	-9.2 ; -590 (0.0000)	-8.5 ; -362 (0.0000)
Klk1b5	ILMN_1224893	-6.0 ; -66 (0.0000)	-6.1 ; -67 (0.0000)	
Klk1b5	ILMN_2731191		-6.5 ; -88 (0.0000)	-8.7 ; -420 (0.0000)
Klk1b8	ILMN_1216962	-6.3 ; -79 (0.0000)	-6.9 ; -121 (0.0000)	
Klk1b9	ILMN_2723594	-5.8 ; -56 (0.0000)	-7.6 ; -187 (0.0000)	-9.7 ; -845 (0.0000)
Klk1b9	ILMN_2784773	-6.3 ; -76 (0.0000)	-7.1 ; -133 (0.0000)	-9.3 ; -648 (0.0000)

P-values given as 0.0000 designate values below 5.0E-05, i.e. values below the Nexus Expression limit.
