# Supplementary material for: Deconvolution of expression microarray data reveals 131I-induced responses otherwise undetected in thyroid tissue
Source: PLoS One. 2018 Jul 12;13(7):e0197911. doi: 10.1371/journal.pone.0197911 (PMC6042689; doi:10.1371/journal.pone.0197911)
Supplement: S6 Table — (PDF) [file pone.0197911.s008.pdf]

Supplemental Table 6. IR associated gene signature for higher and lower C-cell frequency estimates

Gene symbol (synonym)	Probe ID	Thyroid tissue (conv.)	Follicular cells (deconv.)		C-cells (deconv.)	
		log$_2$ ratio; fold change (adjusted p-value)				
			cF = 0.11	cF = 0.05	cF = 0.11	cF = 0.05
Ccnd1 †	ILMN_1221503	-0.92; -1.9 (0.0019)		2.7; 6.6 (0.0022)		
	ILMN_2601471	-0.91; -1.9 (0.0002)				
Ccng1	ILMN_2500276	0.81; 1.8 (0.0076)	-2.5; -5.7 (0.0096)			
Cdkn1a †	ILMN_2846775		-3.4; -11 (0.0001)			
Fos †	ILMN_2750515		5.0; 32 (0.0001)	3.8; 14 (0.0002)		
Gjb2	ILMN_1227148	-2.2; -4.6 (0.0000)				
	ILMN_2999627	-2.9; -7.5 (0.0000)			-5.1; -34 (0.0000)	
Naa35 (Mak10)	ILMN_2828599		-2.6; -6.1 (0.0080)			
Plcg2	ILMN_2601833	-1.1; -2.1 (0.0000)				
Trp53inp1	ILMN_2506012		5.0; 32 (0.0000)			
Trp53inp2	ILMN_2457585		-3.0; -8.0 (0.0024)			

†Note that *Ccnd1* , *Cdkn1a* and *Fos* are reported as both IR-associated and TH-responding in the literature.

Results of transcript regulation of respective signature genes in thyroid tissue adapted from Langen *et al.* (16).

Adjusted p-values given as 0.0000 designate values below 10-5, i.e. values below the Nexus Expression limit.

Conv., convoluted data; deconv., deconvolved data; cF, cell frequency (as used for deconvolution)
