# Supplementary material for: Deconvolution of expression microarray data reveals 131I-induced responses otherwise undetected in thyroid tissue
Source: PLoS One. 2018 Jul 12;13(7):e0197911. doi: 10.1371/journal.pone.0197911 (PMC6042689; doi:10.1371/journal.pone.0197911)
Supplement: S7 Table — (PDF) [file pone.0197911.s009.pdf]

Supplemental Table 7. TH-responding gene signature for higher and lower C-cell frequency estimates

Gene symbol	Probe ID	Thyroid tissue (conv.)	Follicular cells (deconv.)		C-cells (deconv.)	
		log$_2$ ratio; fold change (adjusted p-value)				
			**cF = 0.11**	**cF = 0.05**	**cF = 0.11**	**cF = 0.05**
Atp2a1	ILMN_2666864	5.3; 41 (0.0000)	7.8; 223 (0.0000)	7.5; 178 (0.0000)	11; 1552 (0.0000)	11; 1500 (0.0000)
Camkk1	ILMN_1242310		-2.2; -4.6 (0.0045)			
Camkk2	ILMN_1256263				-3.9; -15 (0.0001)	
Ccnd1	ILMN_1221503	-0.92; -1.9 (0.0019)		2.7; 6.6 (0.0022)		
	ILMN_2601471	-0.91; -1.9 (0.0002)				
Cd44	ILMN_3114585	-0.69; -1.6 (0.0001)	-3.2; -9.2 (0.0000)	-3.0; -8.0 (0.0000)		
	ILMN_2754990				-5.6; -49 (0.0000)	-7.4; -166 (0.0000)
Cdkn1a †	ILMN_2846775		-3.4; -11 (0.0001)			
Egf	ILMN_2684104	-4.9; -31 (0.0000)			-5.3; -39 (0.0000)	-5.3; -40 (0.0000)
Egfr	ILMN_3128725				-4.9; -30 (0.0095)	-5.2; -36 (0.0000)
Fos †	ILMN_2750515		5.0; 32 (0.0001)	3.8; 14 (0.0002)		
Hnrnph3 (Hnrph3)	ILMN_2958912		5.7; 52 (0.0000)			
Lmo2	ILMN_2767605	-1.2; -2.3 (0.0000)				
Mbp	ILMN_3081854		-2.9; -7.5 (0.0037)			
Pck1	ILMN_1213632	1.2; 2.3 (0.0001)		2.2; 4.7 (0.0095)		
Pfkp	ILMN_2673233				5.7; 52 (0.0000)	7.1; 133 (0.0000)
Pik3c2a	ILMN_1252098		-2.5; -5.7 (0.0087)			
Prkag2	ILMN_3161626	0.80; 1.7 (0.0002)				
Prkca	ILMN_1217890		-1.9; -3.7 (0.0092)			
Rcan2	ILMN_3033007		-5.0;-32 (0.0000)	-1.5; -2.7 (0.0003)		
Slc16a6	ILMN_1258950	-0.91; -1.9 (0.0005)				
Slc2a1	ILMN_1258159	-0.94; -1.9 (0.0007)		-3.2; -9.5 (0.0001)		
Sms	ILMN_1232323		-2.3; -4.9 (0.0003)			
Vldlr	ILMN_1218264	1.1; 2.2 (0.0000)				
	ILMN_2515601	1.1; 2.2 (0.0000)				
	ILMN_2796472	1.1; 2.2 (0.0000)				

†Note that *Ccnd1* , *Cdkn1a* and *Fos* are reported as both IR-associated and TH-responding in the literature.
Results of transcript regulation of respective signature genes in thyroid tissue adapted from Langen *et al.* (16).
Adjusted p-values given as 0.0000 designate values below 10-5, i.e. values below the Nexus Expression limit.
Conv., convoluted data; deconv., deconvolved data; cF, cell frequency (as used for deconvolution)
